# Burden of Cardiovascular Disease in Asian Countries, 1990-2021

**DOI:** 10.1016/j.jacasi.2025.10.023

**Published:** 2026-02-20

**Authors:** Le Li, Xi Peng, Lingmin Wu, Zhicheng Hu, Limin Liu, Minghao Zhao, Tao Zhang, Ligang Ding, Lihui Zheng, Yan Yao

**Affiliations:** Fuwai Hospital, National Center for Cardiovascular Diseases, Chinese Academy of Medical Sciences, Peking Union Medical College, Beijing, China

**Keywords:** cardiovascular disease, disability-adjusted life years, epidemiology, mortality, prevalence

## Abstract

**Background:**

Cardiovascular diseases (CVDs) account for a large and increasing health burden worldwide, as shown in the Global Burden of Disease Study 2021. However, there has been no comprehensive assessment of CVDs in Asia, which bears the largest population globally.

**Objectives:**

This study sought to evaluate the burden of CVD in Asia from 1990 to 2021.

**Methods:**

Mortality, prevalence, and disability-adjusted life years (DALYs) along with their age-standardized rates (ASRs) per 100,000 population from 1990 to 2021 were used to measure the CVDs burden. Further subanalyses were conducted based on age group and sex.

**Results:**

In 2021, CVDs caused an estimated 11.9 million deaths (95% uncertainty interval [UI]: 10.9-12.9 million deaths), 340.4 million prevalent cases (95% UI: 315.1-366.9 million prevalent cases), and 270.4 million DALYs (95% UI: 251.6-290.2 million DALYs). Although the ASR of prevalence slightly increased from 1990 to 2021 with a percentage change of 5.7% (95% UI: 3.9%-8.1%), the ASRs of death and DALYs were significantly decreased, with percentage changes of −28.2% (95% UI: −42.2% to −12.5%) and −37.8% (95% UI: −50.5% to −24.3%), respectively. The Asian burden of CVDs was higher in males and the elderly. The primary contributors to DALYs across Asia were ischemic heart disease, stroke, and hypertensive heart disease.

**Conclusions:**

Burden of CVDs in Asia remains substantial. Certain populations, including males and the elderly, experienced a heavier burden of CVDs.

Cardiovascular disease (CVD) have emerged as a leading cause of morbidity and mortality worldwide, posing a significant public health challenge across both developed and developing nations.^1^ In recent decades, Asia has experienced a marked increase in the burden of CVD, driven by multifactorial influences including longer life expectancy, aging populations, urbanization, and lifestyle shifts.^2^^,^^3^

The rapid economic and infrastructural development witnessed in many Asian countries offers a significant opportunity to enhance the detection and management of CVD. However, the escalating burden of CVD poses substantial socioeconomic and policy challenges, especially in low- and middle-income countries, where limited resources often impede efforts to implement comprehensive prevention strategies, ensure early detection, and maintain long-term management.^4^ A comprehensive and comparative assessment of the CVD burden across Asia is imperative to elucidate its temporal trends, regional variations, and underlying risk factors. Such insights are pivotal for informing evidence-based policy formulation, enhancing resource allocation strategies, and developing targeted interventions.

Previous studies estimated the CVD burden at the global level^5^ or in individual countries of Asia,^6^ or have focused on individual CVD in Asia such as ischemic stroke,^7^ rheumatic heart disease (RHD),^8^ and ischemic heart disease (IHD).^9^ However, only a limited number of investigations have offered a region-wide, up-to-date assessment of the full spectrum of CVD and their temporal trends across Asia. Moreover, the estimates provided by these studies were often based on outdated data, limiting their relevance to current epidemiological trends and the accuracy of the most recent estimates. This study aims to systematically investigate the burden of 12 CVDs in 49 Asian countries from 1990 to 2021, focusing on trends in prevalence, incidence, mortality, and disability-adjusted life years (DALYs). By offering a comprehensive analysis of the epidemiological landscape and temporal patterns of CVD across the region, this study seeks to provide valuable insights to support the development of country-specific strategies and policies aimed at addressing one of Asia's most pressing public health challenges.

## Methods

### Overview of the Global Burden of Disease Study

The Global Burden of Disease Study 2021 (GBD 2021) provides a thorough analysis of key health metrics, including incidence, mortality, prevalence, years of life lost, years lived with disability, and DALYs. This evaluation covers 371 diseases and injuries, 288 causes of death, and 88 risk factors across 204 countries and territories.^10^, ^11^, ^12^, ^13^ Data sources include population-based surveys, vital registration systems, administrative databases, and scientific literature. Estimates are updated annually, integrating new epidemiological data, refining methods, and incorporating additional risk factors. The general methodology of GBD 2021 is outlined elsewhere.^10^^,^^11^^,^^13^ The study follows the GATHER guidelines for accurate and transparent health estimates reporting.^14^

### Estimation of disease burden of CVD

In this study, CVDs contained 12 diseases, including RHD, IHD, stroke, hypertensive heart disease, nonrheumatic valvular heart disease, cardiomyopathy and myocarditis, pulmonary artery hypertension, atrial fibrillation and flutter, aortic aneurysm, lower extremity peripheral arterial disease, endocarditis, and other cardiovascular and circulatory diseases. The diagnoses of the 12 diseases were established based on the International Classification of Diseases–Ninth Revision or International Classification of Diseases–10th Revision. Detailed diagnostic criteria can be found in Supplemental Table 1. CODEm (Cause of Death Ensemble modelling) integrates location-specific covariates to generate smoothed mortality trends for 204 countries and territories, borrowing statistical strength across age groups, locations, and years. DisMod MR-2.1 model, a Bayesian meta-regression tool, adjusts for study-level differences in measurement methods and case definitions.^11^

### Attributable risk factors

The selection of risk factors was guided by rigorous criteria, including robust evidence supporting their association with CVD, the availability of exposure data, and their potential for modification.^12^ In GBD 2021, 12 key risk factors were identified as contributors to CVD-related mortality: air pollution, alcohol consumption, dietary risks, elevated low-density lipoprotein (LDL) cholesterol, high body mass index, increased fasting plasma glucose, elevated systolic blood pressure, impaired kidney function, lead exposure, physical inactivity, suboptimal temperature, and tobacco use. For each exposure-outcome pair, GBD 2021 reports population-attributable fraction stratified by age, sex, location, and year, together with the corresponding numbers of attributable deaths and DALYs. These estimates represent the reduction in CVD burden that could be achieved if population exposure were shifted to the theoretical minimum-risk distribution defined by GBD 2021. Detailed definitions of these risk factors and methodologies for assessing their contributions to CVD-related mortality have been extensively documented elsewhere.^15^

### Statistical analysis

To evaluate the burden of CVD, annual data on prevalence, incidence, deaths, and DALYs were analyzed, including their age-standardized rates (ASRs) and 95% uncertainty intervals (UIs). ASRs were instrumental in minimizing biases arising from variations in age structure and population size. All rates are expressed per 100,000 individuals. Subgroup analyses were performed, categorizing data by age groups (<5 years, 5-year intervals, and >95 years) and sex (male and female). The sociodemographic index (SDI), a composite measure reflecting national development status based on per-capita income, education levels, and fertility rates, was utilized for contextualizing findings. We employed a nonparametric LOESS (locally estimated scatterplot smoothing) approach to analyze and visualize the nonlinear relationship between the SDI and the age-standardized DALYs rate for CVD. This method does not require predefined artificial cutoff points for the SDI, allowing it to flexibly capture complex continuous trends directly from the data. The analysis was based on data from Asian countries sourced from GBD 2021. LOESS fitting and visualization were performed using the ggplot2 package in R software (R Foundation for Statistical Computing), specifically the geom_smooth() function with the parameters set to method = ‘loess’ and span = 1.0. In addition, the relationship between ASR and year is shown using a restricted cubic spline model, which avoids the loss of information due to categorization and reduces subjectivity as compared with the discrete treatment of continuous variables. Proportional contributions of each risk factor to the overall CVD DALYs were computed using the population-attributable fraction method, in accordance with the GBD 2021 framework. This approach incorporated risk exposure distributions, relative risks, and theoretical minimum-risk exposure levels, with adjustments for mediation effects and multirisk aggregation.^12^ Statistical analyses were conducted using R software version 4.1.0.

## Results

### Trends in CVD burden in Asia

**Figure 1:**
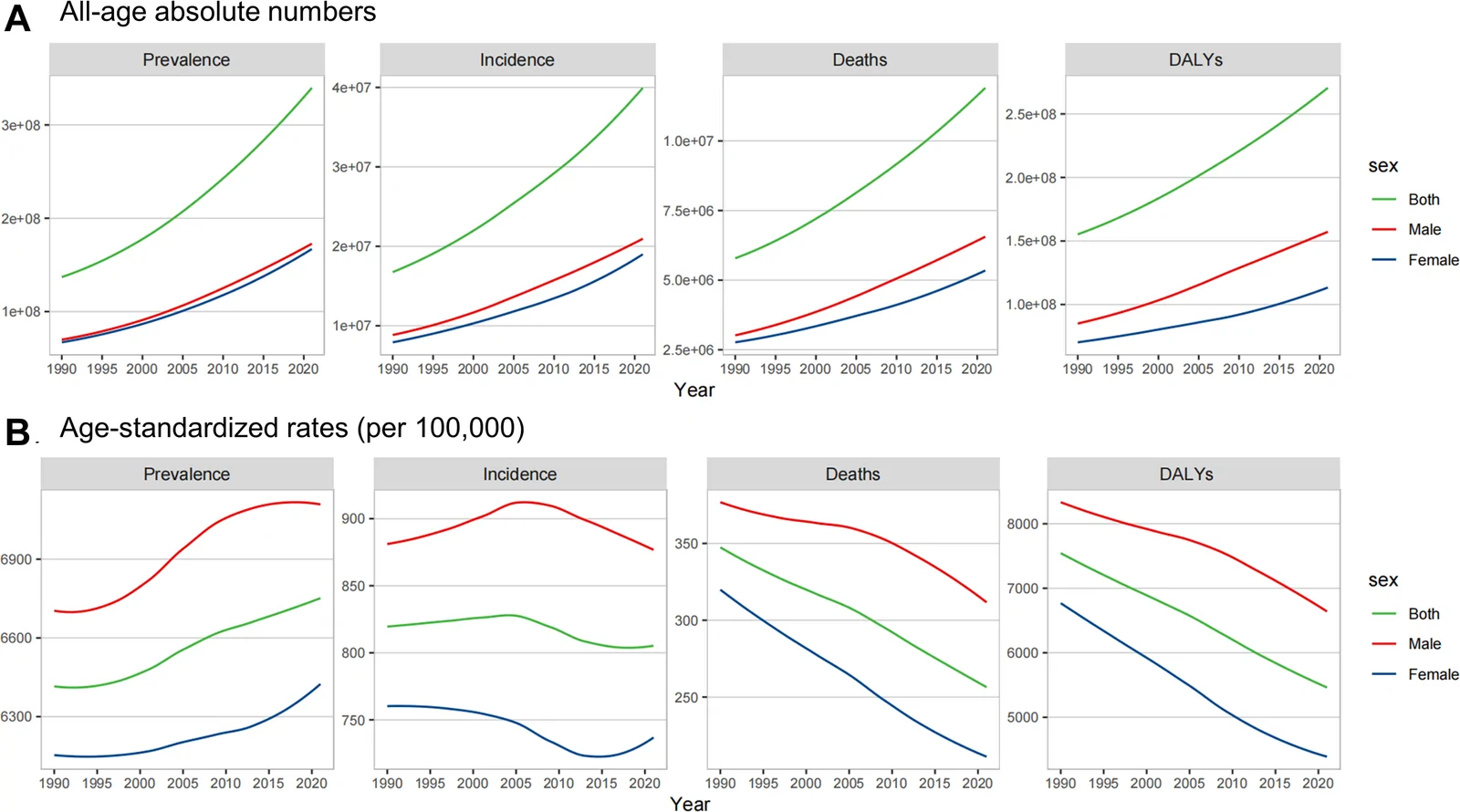
Temporal Patterns of Cardiovascular Disease Burden in Asia, 1990-2021 (A) All-age counts and (B) age-standardized rates (per 100,000). The absolute number of cases showed a consistent upward trend. For age-standardized rates, the prevalence continued to rise gradually, while the incidence exhibited a fluctuating trend. In contrast, both death and disability-adjusted life year (DALY) rates decreased significantly.

**Figure 2:**
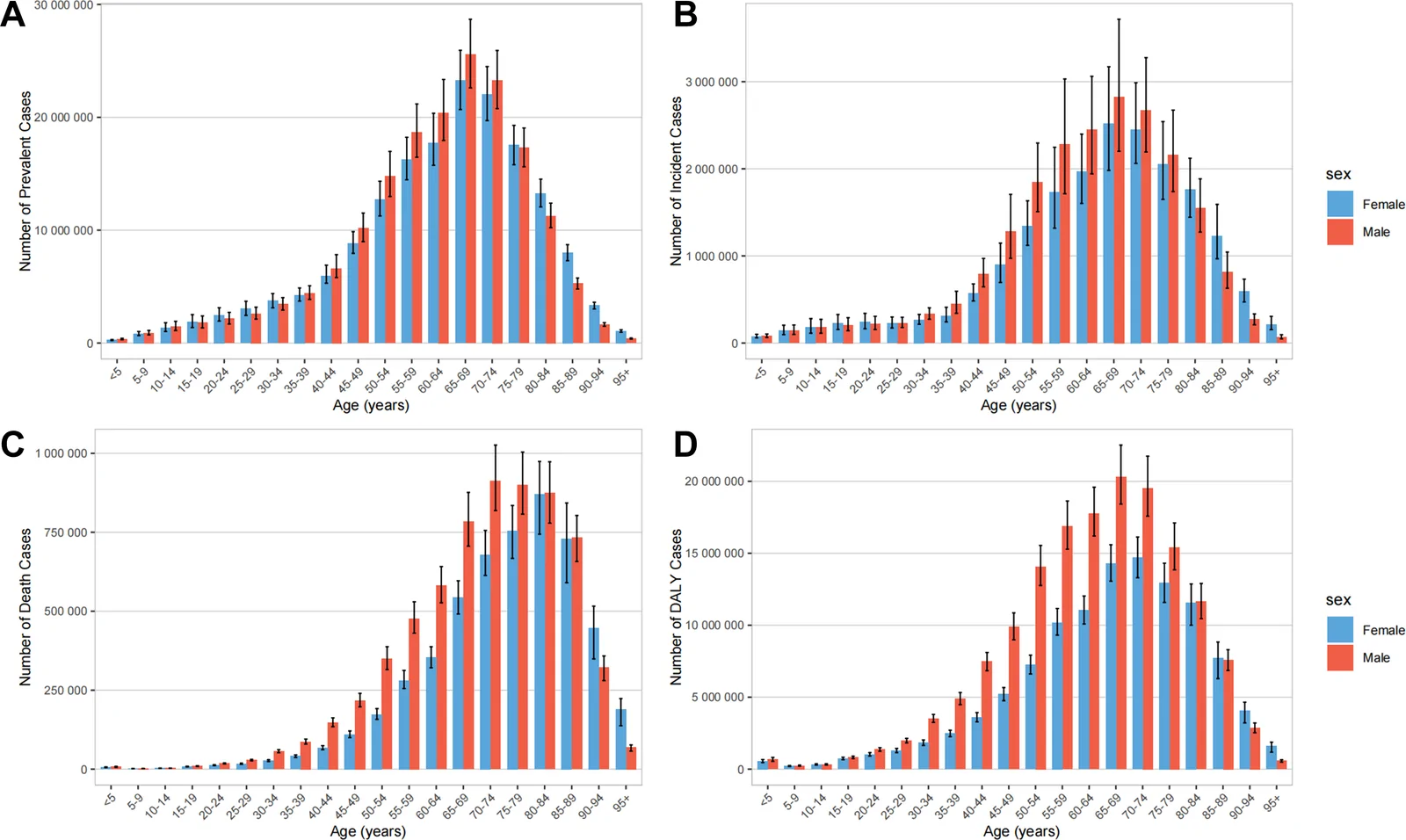
Burden of Cardiovascular Diseases in 2021 in Asia by Age (A) Prevalent cases; (B) Incident cases; (C) Death cases; (D) disability-adjusted life year (DALY) cases. The highest number of deaths occurred in the 70 to 84 years age group, while the peak burdens for prevalence, incidence, and DALYs were observed in the 60 to 74 years age group.

Between 1990 and 2021, the burden of CVD in Asia showed notable shifts in both absolute numbers and ASRs. The age-standardized mortality rate fell by 26.0% (95% UI: −32.6% to −18.4%), and the DALYs declined by 27.5% (95% UI: −33.4% to −20.8%), indicating real improvements in survival and disease severity after adjusting for age structure. The age-standardized prevalence of CVD increased slightly by 5.7% (95% UI: 3.9%-8.1%), from 6,416.1 per 100,000 (95% UI: 5,994.4-6,822.4) in 1990 to 6,782.6 per 100,000 (95% UI: 6,292.4-7,301.6) in 2021, indicating a slight rise in overall disease burden due to population growth and aging (Figure 1). In contrast, the age-standardized incidence rate of CVD showed a slight decrease by −1.7% (95% UI: −3.0% to −0.5%), from 820.3 per 100,000 (95% UI: 746.6-900.1) in 1990 to 806.1 per 100,000 (95% UI: 728.8-887.6) in 2021 (Supplemental Table 2). Demographic expansion, however, drove sharp growth in absolute counts: prevalent cases increased by 149.9% (from 136 million to 340 million; 95% UI: 143.7%-157.7%), deaths by 106.1% (95% UI: 86.3%-127.9%), and DALYs by 74.5% (95% UI: 59.8%-90.6%). The trends in the burden of CVD—reflected by ASRs of prevalence, incidence, deaths, and DALYs in Asia from 1990 to 2021—as analyzed using restricted cubic spline, are presented in Supplemental Figure 1. All *P* values for nonlinearity were <0.05. A supplemental analysis that separated the pre– and post–COVID-19 periods (Supplemental Table 3) confirmed that the pandemic further amplified the recent CVD burden. The burden peaked in adults 65 to 75 years of age in 2021, and across almost all age groups men were more affected than women, a pattern that reversed only beyond 80 years of age (Figure 2).

**Figure 3:**
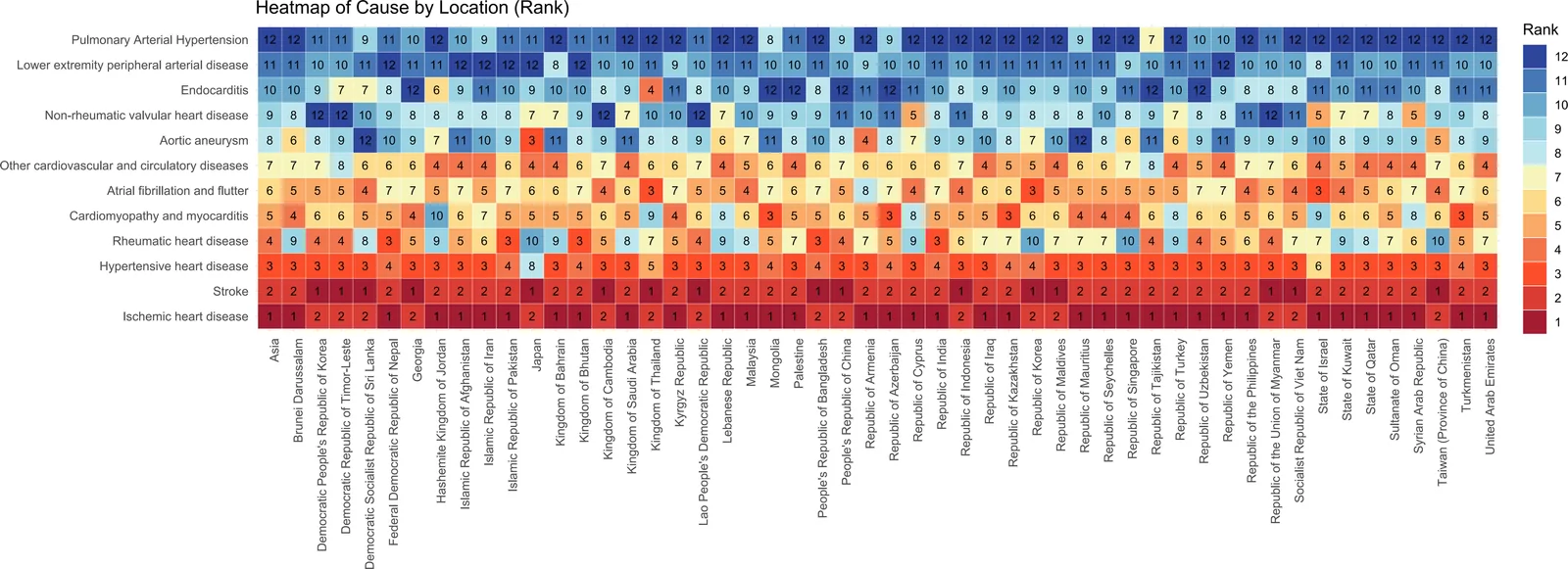
Ranking the Burden of Cardiovascular DALYs in Asia, 2021 Ischemic heart disease, stroke, and hypertensive heart disease were the 3 leading causes of disability-adjusted life years (DALYs) in the vast majority of Asian countries.

For the age-standardized DALYs, IHD, stroke, and hypertensive heart disease were the most dominant CVDs in the most Asian countries in 2021 (Figure 3). The burden of CVD in Asia varied significantly across countries, with considerable changes observed from 1990 to 2021 (Supplemental Table 4). Central Asia had the highest age-standardized prevalence (8,144.0 per 100,000) (95% UI: 7,697.7-8,542.4), showing a modest increase of 5.1% (95% UI: 3.5%-6.6%) over the past 3 decades. Among individual countries, Uzbekistan saw the largest increase in prevalence (18.4%; 95% UI: 15.7%-20.9%), while Kyrgyzstan experienced a notable decline (−7.5%; 95% UI: −9.7% to −5.4%). In East Asia, the overall prevalence was lower (6601.7 per 100,000; 95% UI: 6,123.7-7,076.6), with a 9.2% (95% UI: 15.7%-20.9%) increase from 1990 to 2021. Japan, however, showed a significant decrease in prevalence (−9.5%; 95% UI: −12.0% to −6.6%). Regarding incidence, Uzbekistan had the highest rate (1,721.9 per 100,000; 95% UI: 1,633.5-1,826.6), with a marked rise of 65.2% (95% UI: 56.6%-75.2%) from 1990. Central Asia (1280.2 per 100,000; 95% UI: 1,202.2-1,376.2) and Southeast Asia (5,832.5 per 100,000; (95% UI: 5,460.9-6,152.7) also exhibited relatively high incidence rates, with Southeast Asia showing a slight decline (−3.5%; 95% UI: −4.7% to −2.2%). Japan reported the lowest incidence (423.2 per 100,000; 95% UI: 395.9-454.3). In terms of DALYs, Central Asia, particularly Uzbekistan (9,428.7 per 100,000; 95% UI: 8,285.0-10,587.0) and Turkmenistan (11,751.3 per 100,000; 95% UI: 9,600.2-14,451.2), reported the highest burden, with significant increases in DALYs over the past 30 years. The burden of CVDs and its subtypes across Asian countries is further illustrated in Supplemental Figures 2 to 17. Moreover, the age-standardized prevalence, incidence, death, and DALY rates of CVD by sex in 2021 for all Asian countries included in the GBD study are illustrated in Supplemental Figures 18 to 21.

### Disease-specific burden

#### Ischemic heart disease

Over the past 3 decades, mortality and DALYs attributable to IHD fell on an age-standardized basis by 3.1% (95% UI: −11.1% to 5.4%) and 5.5% (95% UI: −13.0% to 2.7%), respectively; nevertheless, the absolute numbers of deaths and DALYs surged by 131.3% (95% UI: 112.3%-152.7%) and 192.8% (95% UI: 180.9%-206.4%). IHD also exhibited an increase in age-standardized prevalence of 11.8% (95% UI: 7.3%-17.3%), while the absolute number of prevalent cases climbed by 192.8% (95% UI: 180.9%-206.4%), from 554.6 million (95% UI: 483.1-631.6 million) in 1990 to 1,624.1 million (95% UI: 1,398.9-1,913.5 million) in 2021. During these 30 years, both prevalence and incidence followed a continuous upward trajectory, although the pace appears to have slowed (Supplemental Figure 22). The age-standardized incidence rate rose only modestly, by 4.4% (95% UI: 3.0%-5.9%), yet the absolute number of incident cases more than doubled. The highest age-standardized prevalence was recorded in the United Arab Emirates at 7,608.9 per 100,000 (95% UI: 6,995.2-8,286.0), whereas the Syrian Arab Republic bore the greatest DALYs burden at 6,688.8 per 100,000 (95% UI: 5,230.4-8,518.9) (Supplemental Table 5, Supplemental Figure 23). Across age and sex strata, the burden remained consistently higher in males than females, rising sharply from the 30 to 34 years age group and peaking at 65 to 69 years of age (Supplemental Figure 24).

#### Stroke

Age-standardized stroke mortality fell by 37.4% (95% UI: −44% to −29.2%) and DALYs by 38.2% (95% UI: −44.4% to −30.8%), yet absolute stroke deaths and DALYs still climbed by 75.6% (95% UI: 56.5%-98.6%) and 52.4% (95% UI: 36.7%-70.9%), respectively. Age-standardized prevalence fell by 2.4% (95% UI: −4% to −0.9%), and incidence by 16.6% (95% UI: −19.6% to −13.4%). Although prevalence has steadily declined across the 30-year span, incidence appears to have trended upward during the past 5 years (Supplemental Figure 25). Absolute prevalence and incidence counts nevertheless rose by 118.8% (95% UI: 114.0%-124.1%) and 110.8% (95% UI: 101.9%-119.6%). The highest age-standardized prevalence was recorded in the United Arab Emirates at 7,608.9 per 100,000 (95% UI: 6,995.2-8,286.0), whereas Indonesia bore the greatest DALYs burden at 4,587.1 per 100,000 (95% UI: 3,889.4 -5,248.2) (Supplemental Table 6, Supplemental Figure 26). Overall, stroke burden remained higher in men than in women, peaking between 65 and 75 years of age (Supplemental Figure 27).

#### Atrial fibrillation and flutter

Age-standardized mortality from atrial fibrillation and flutter rose by 5.3% (95% UI: −14.5% to 27.6%), and the accompanying DALYs edged up by 3.4% (95% UI: −8.5% to 15.4%); nevertheless, absolute deaths and DALYs surged by 289.6% (95% UI: 215.9%-377.3%) and 210.7% (95% UI: 179.1%-247.7%), respectively (Supplemental Figure 28). Age-standardized prevalence increased by 4.2% (95% UI: 2.4%-6.0%), while the number of prevalent cases grew by 194.6% (95% UI: 188%-202%), climbing from 87.4 million (95% UI: 69.3-114 million) in 1990 to 257.4 million (95% UI: 204.3-335.9 million) in 2021. Incidence was largely stable, with only a 0.9% rise in age-standardized terms, yet absolute incident cases expanded by 173.5% (95% UI: 164.7%-182.2%). Israel recorded both the highest age-standardized prevalence—1,155.5 per 100,000 (95% UI: 958.3-1,312.4)—and the highest DALYs, at 148.8 per 100,000 (95% UI: 117.3-184.4) (Supplemental Table 7, Supplemental Figure 29). DALYs were higher in men up to 75 years of age, after which women bore the greater burden; overall burden peaked between 70 and 85 years of age (Supplemental Figure 30).

#### Hypertensive heart disease

Age-standardized mortality and DALYs from hypertensive heart disease fell by 40.5% (95% UI: −51.5% to −19.8%) and 41.7% (95% UI: −51.2% to −21.3%), respectively, yet the absolute numbers of deaths and DALYs still rose by 76.2% (95% UI: 44.6%-139.7%) and 54.4% (95% UI: 28.5%-111.3%). Age-standardized prevalence declined by only 2.7% (95% UI: −9.9% to 4.4%), whereas absolute prevalent cases surged by 172.8% (95% UI: 150.8%-192.8%). After falling steadily from 1990 to 2010, hypertensive heart disease prevalence accelerated sharply during 2010 to 2021 (Supplemental Figure 31), suggesting that population aging is driving a heavier disease burden despite improvements in rates. The highest age-standardized prevalence was observed in Jordan, at 341.9 per 100,000 (95% UI: 268.8-427.8), while Afghanistan exhibited the greatest DALYs burden at 1,501.8 per 100,000 (95% UI: 790.5-2,264.0) (Supplemental Table 8, Supplemental Figure 32). By age and sex, males carry a higher burden than females at 60 to 64 years of age, but from 65 to 69 years of age onward, the burden in females surpasses that in males (Supplemental Figure 33).

#### Rheumatic heart disease

Between 1990 and 2021, RHD in Asia experienced a pronounced epidemiological transition. The age-standardized mortality rate fell by 59.8% (95% UI: −68.2% to −50.2%), yet population growth meant that deaths declined only slightly—from about 0.34 million (95% UI: 0.28-0.39) to 0.32 million (95% UI: 0.27-0.39), a 5.4% (95% UI: −23.7% to 17.3%) reduction. Similarly, the age-standardized DALYs dropped by 56.7% (95% UI: −64.0% to −48.1%), while absolute DALYs dropped by 16.5% (95% UI: −29.8% to −1.0%). Age-standardized prevalence remained essentially unchanged (-0.1%) (95% UI: −2.1% to 1.7%), while prevalent cases grew by 49.9% (95% UI: 43.2%-56.1%). Incidence followed the same pattern: a stable ASR (42.5-42.7 per 100,000) accompanied by a 23.2% (95% UI: 19.4%-27.4%) rise in incident cases. Notably, prevalence and incidence declined between 1990 and 2000 but have trended upward ever since, underscoring the ongoing need for effective control strategies (Supplemental Table 9, Supplemental Figure 34). The findings suggest improvements in outcomes but a growing absolute burden, likely driven by an increasing incidence in certain regions. The highest age-standardized prevalence was observed in Armenia, with 1,114.3 per 100,000 (95% UI: 879.7-1,368.2), and the highest DALYs was in Pakistan at 585.0 per 100,000 (95% UI: 443.3-816.9) (Supplemental Figure 35). From the perspective of age and sex distribution, the burden is consistently higher in females than in males, with the peak occurring in the 55 to 59 years age group (Supplemental Figure 36).

#### Nonrheumatic valvular heart disease

For nonrheumatic valvular heart disease, age-standardized mortality declined by 11.3% (95% UI: −21.7% to −2.5%) and the DALYs dropped by 16.7% (95% UI: −25.0% to −8.3%); however, absolute DALYs still rose by 111.1% (95% UI: 83.6%-140.6%). Meanwhile, age-standardized prevalence increased by 11.4% (95% UI: 8.6%-14.3%), with absolute cases up 206.6% (95% UI: 197.5%-216.3%). Incidence showed a modest 8.1% rise in age standardized terms (95% UI: 5.2%-11.0%), and the absolute number of incident cases surged by 172.4% (95% UI: 164.5%-180.8%); both prevalence and incidence have climbed steadily over time (Supplemental Figure 37). Georgia recorded the highest age-standardized prevalence, at 1206.0 per 100,000 (95% UI: 1,048.2-1,383.9), while Cyprus had the greatest DALYs at 124.5 per 100,000 (95% UI: 99.9-153.8) (Supplemental Table 10, Supplemental Figure 38). The burden rises with age in both sexes but increases more sharply among females (Supplemental Figure 39).

#### Cardiomyopathy and myocarditis

For cardiomyopathy and myocarditis, age-standardized mortality declined by 17.7% (95% UI: −31.0% to 1.4%) and the age standardized DALYs fell by 16.7% (95% UI: −30.8% to 2.5%); however, absolute DALYs still rose by 51.3% (95% UI: 19.9%-88.4%). Age-standardized prevalence increased by 27.1% (95% UI: 19.9%-34.7%), with absolute cases up 117.3% (95% UI: 102%-134%). Incidence dropped modestly by 3.5% (95% UI: −4.5% to −2.3%), yet absolute incident cases grew by 107.3% (95% UI: 68.8%-159.5%) (Supplemental Figure 40). Kazakhstan recorded both the highest age-standardized prevalence—146.5 per 100,000 (95% UI: 116.3-175.2)—and the greatest DALYs at 1,202.9 per 100,000 (95% UI: 979.7-1,442.3) (Supplemental Table 11, Supplemental Figure 41). Across ages, the burden remains consistently higher in males, with a bimodal distribution: a first peak in children younger than 5 years of age and a second peak at 55 to 59 years of age (Supplemental Figure 42).

#### Pulmonary arterial hypertension

For pulmonary arterial hypertension, age-standardized mortality decreased by 21.8% (95% UI: −43.1% to 4.7%) and the age-standardized DALYs fell by 34.7% (95% UI: −49.7% to −17.4%); absolute DALYs, however, were essentially unchanged, rising only 0.7% (95% UI: −27.1% to 32%). Age-standardized prevalence changed little, edging up 1.9% (95% UI: 1.1%-2.7%), while prevalent cases nearly doubled, increasing 97.5% (95% UI: 85.3%-109.7%) from 0.5 million (95% UI: 0.4-0.6 million) in 1990 to 1.0 million (95% UI: 0.8-1.3 million) in 2021. Incidence remained stable, with a 2.0% rise in age-standardized terms (95% UI: 1.3%-2.6%), yet absolute incident cases climbed 96.9% (95% UI: 85.7%-107.6%) (Supplemental Figure 43). Israel recorded the highest age-standardized prevalence at 4.4 per 100,000 (95% UI: 3.6-5.3), whereas Mongolia had the greatest DALYs at 43.9 per 100,000 (95% UI: 25.6-56.5) (Supplemental Table 12, Supplemental Figure 44). The burden is heavily concentrated in children under 5 years of age, with comparable levels across older age groups (Supplemental Figure 45).

#### Aortic aneurysm

Aortic aneurysm showed a significant increase in both age-standardized DALYs and deaths, with 30.6% (95% UI: 9.2%-53.1%) and 39.5% (95% UI: 20.3%-59.6%) increases in the respective measures (Supplemental Figure 46). The absolute number of deaths increased by 286.9% (95% UI: 228%-349.3%), and DALYs increased by 220.7% (95% UI: 163.9%-282.9%). The highest DALYs was in Armenia, at 192.8 per 100,000 (95% UI: 159.7-228.2) (Supplemental Table 13, Supplemental Figure 47). Overall, the burden of aortic aneurysm was higher in men compared with women, with DALYs peaking in the 70 to 74 years age group (Supplemental Figure 48).

#### Lower extremity peripheral arterial disease

For lower extremity peripheral arterial disease, mortality remained essentially unchanged, while the age standardized DALYs fell by 6.2% (95% UI: −12.5% to 1.6%). Age-standardized prevalence decreased by 1.0% (95% UI: −1.9% to −0.2%), yet absolute prevalent cases grew by 165.8% (95% UI: 162.2%-170.1%). Incidence showed a similar pattern, declining 2.4% in age-standardized terms (95% UI: −3.2% to −1.5%) but rising 150.5% in absolute numbers (95% UI: 146.6%-154.6%) (Supplemental Figure 49). Cyprus recorded the highest age-standardized prevalence, at 2,052.4 per 100,000 (95% UI: 1,768.4-2,380.4), while Armenia had the greatest DALYs, at 43.2 per 100,000 (95% UI: 35.5-52.0) (Supplemental Table 14, Supplemental Figure 50). Across sexes, the burden was consistently higher in females than in males (Supplemental Figure 51).

#### Endocarditis

Age-standardized mortality from endocarditis declined by 15.4% (95% UI: −27.9% to −1.6%), and the age-standardized DALYs fell by 25.7% (95% UI: −37.4% to −10.7%). In contrast, age-standardized prevalence increased by 62.6% (95% UI: 55.4%-70.3%), and absolute cases rose by 170.1% (95% UI: 151.2%-190.8%). Both prevalence and incidence have shown a continuous upward trend; incidence climbed by 29.7% (95% UI: 20.8%-39.8%), with absolute incident cases increasing accordingly (Supplemental Figure 52). Thailand recorded the highest age-standardized prevalence, at 19.4 per 100,000 (95% UI: 16.6-22.2), and the greatest DALYs, at 77.8 per 100,000 (95% UI: 59.1-105.6) (Supplemental Table 15, Supplemental Figure 53). The burden displays a bimodal age distribution, peaking in children under 5 years of age and adults 40 to 49 years of age, and DALYs are higher in men until about 80 years of age, after which the trend reverses (Supplemental Figure 54).

#### Other cardiovascular and circulatory diseases

For other cardiovascular and circulatory diseases, age-standardized mortality fell by 20.8% (95% UI: −38.4% to −5.4%), and the age-standardized DALYs dropped by 11.2% (95% UI: −22.1% to 4%), yet absolute DALYs still climbed by 68.2% (95% UI: 43.5%-108.6%). Age-standardized prevalence increased by 28.3% (95% UI: 19.4%-37.2%), with absolute cases rising 201% (95% UI: 177.9%-222.5%) (Supplemental Figure 55). Oman recorded the highest DALYs at 502.2 per 100,000 (95% UI: 408.9-613.6) (Supplemental Table 16, Supplemental Figure 56). The burden remains higher in men than in women, peaking between 65 and 75 years of age (Supplemental Figure 57).

### Percentage of DALYs attributable to risk factors for CVDs

For CVDs, the most significant risk factors include high systolic blood pressure (50.7%), dietary risks (33.0%), and air pollution (28.3%) (Supplemental Table 17). While most risk factors were similar between men and women, tobacco use was notably more prominent among male patients (Supplemental Figures 58 and 59). High systolic blood pressure, dietary risks, and elevated LDL cholesterol were identified as the primary risk factors for IHD, as anticipated. Additionally, air pollution emerged as a significant contributor to the burden of IHD, accounting for 35.4% of DALYs. High systolic blood pressure was identified as the most significant risk factor for stroke, contributing to more than 50% of DALYs. For aortic aneurysm, smoking was identified as the predominant risk factor in male patients (49.0%), while high blood pressure was the leading risk factor in female patients (16.8%).

The analysis of risk factors for IHD, stroke, hypertensive heart disease, atrial fibrillation and flutter, aortic aneurysm, and lower extremity peripheral arterial disease in both men and women across various Asian countries can be found in Supplemental Figures 60 to 71.

### Temporal trends in modifiable risk factors

**Figure 4:**
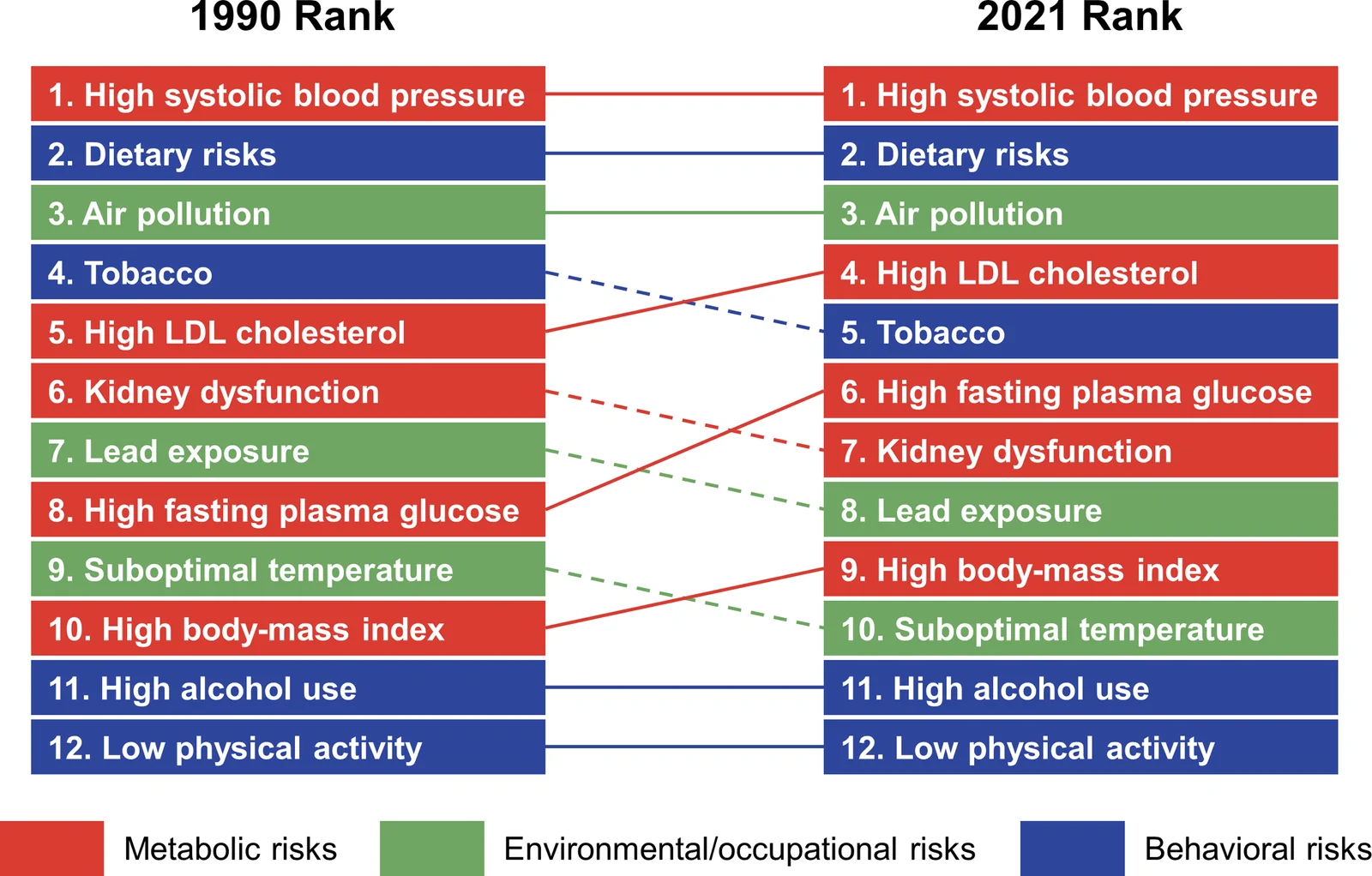
Trends in Modifiable Cardiovascular Disease Risk Factors Across Asia, 1990-2021 From 1990 to 2021, high systolic blood pressure, dietary risks, and air pollution remained the leading risk factors for cardiovascular diseases. LDL = low-density lipoprotein.

Between 1990 and 2021, the major risk factors for CVDs remained high blood pressure, dietary risks, and air pollution (Figure 4). Although smoking decreased compared with 1990, it remains a significant contributor to the CVD burden. Other environmental factors, such as lead exposure and suboptimal temperature, showed a decline since 1990. However, high LDL cholesterol, high body mass index, and high fasting plasma glucose have all increased since 1990.

### Age and sex pattern of CVD risk factors

In 2021, high systolic blood pressure remained the most significant risk factor for CVD, with DALYs peaking in the 65 to 69 years age group. Notably, this risk factor was more pronounced in men than in women before 79 years of age, after which the trend reversed (Supplemental Figure 72). Elevated LDL cholesterol exhibited a trend toward affecting younger populations, reaching its peak impact in the 55 to 59 years age group. Additionally, the DALYs associated with elevated LDL cholesterol were higher in men than in women before 79 years of age (Supplemental Figure 73). Prior to 69 years of age, DALYs of CVD attributable to high body mass index were more pronounced in men (Supplemental Figure 74). DALYs of CVD due to high fasting plasma glucose, air pollution, suboptimal temperature, lead exposure, tobacco, alcohol use, dietary risks, low physical activity, and impaired kidney function in different ages and sex can be found in Supplemental Figures 75 to 83.

### Association with the SDI

In 2021, we observed a nearly linear correlation between the SDI and age-standardized DALYs of CVD across Asian countries. As the SDI increased, the burden of CVD exhibited a gradual decline (Supplemental Figure 84). Specifically, when the SDI was below 0.45, the age-standardized DALYs for RHD remained largely unaffected by changes in SDI. However, when the SDI exceeded 0.45, a negative linear relationship emerged (Supplemental Figure 85). From 1990 to 2021, as the SDI increased, the burden of RHD demonstrated a consistent decline (Supplemental Figure 86). Relationships between other subtypes of CVDs and SDI are detailed in Supplemental Figures 87 to 108.

## Discussion

CVD persists as the predominant cause of both mortality and premature mortality in Asia. In 2019, the region accounted for 58% (10.8 million) of the 18.6 million global CVD-related deaths.^16^ Characterized by its vast population size, marked heterogeneity in ethnic composition, cultural norms, socioeconomic status, and health care systems, Asia faces unique challenges in CVD prevention and management. Timely and nuanced data on the epidemiological patterns and burden of CVD across Asian nations are critical for understanding these challenges and formulating evidence-based policies and targeted interventions to address the CVD pandemic.

This study presents the first systematic and comprehensive analysis of CVD-attributable disease burden in Asia and its constituent regions from 1990 to 2021 (Central Illustration).The main findings are as follows: 1) although the age-standardized prevalence showed a slight increase, the age-standardized incidence, death, and DALY rates experienced a significant decline during this period; 2) although the absolute numbers of prevalent cases, incident cases, deaths, and DALYs attributable to CVD in Asia have climbed sharply over recent decades, the age-standardized incidence, mortality, and prevalence rates have overall declined; 3) males and the elderly generally experienced a heavier burden of CVD compared with other demographic groups; 4) IHD represents the most substantial burden among CVDs, and high systolic blood pressure was identified as the most significant risk factor; and 5) as the SDI increased, the burden of CVD in Asia demonstrated a consistent decline.

**Central Illustration:**
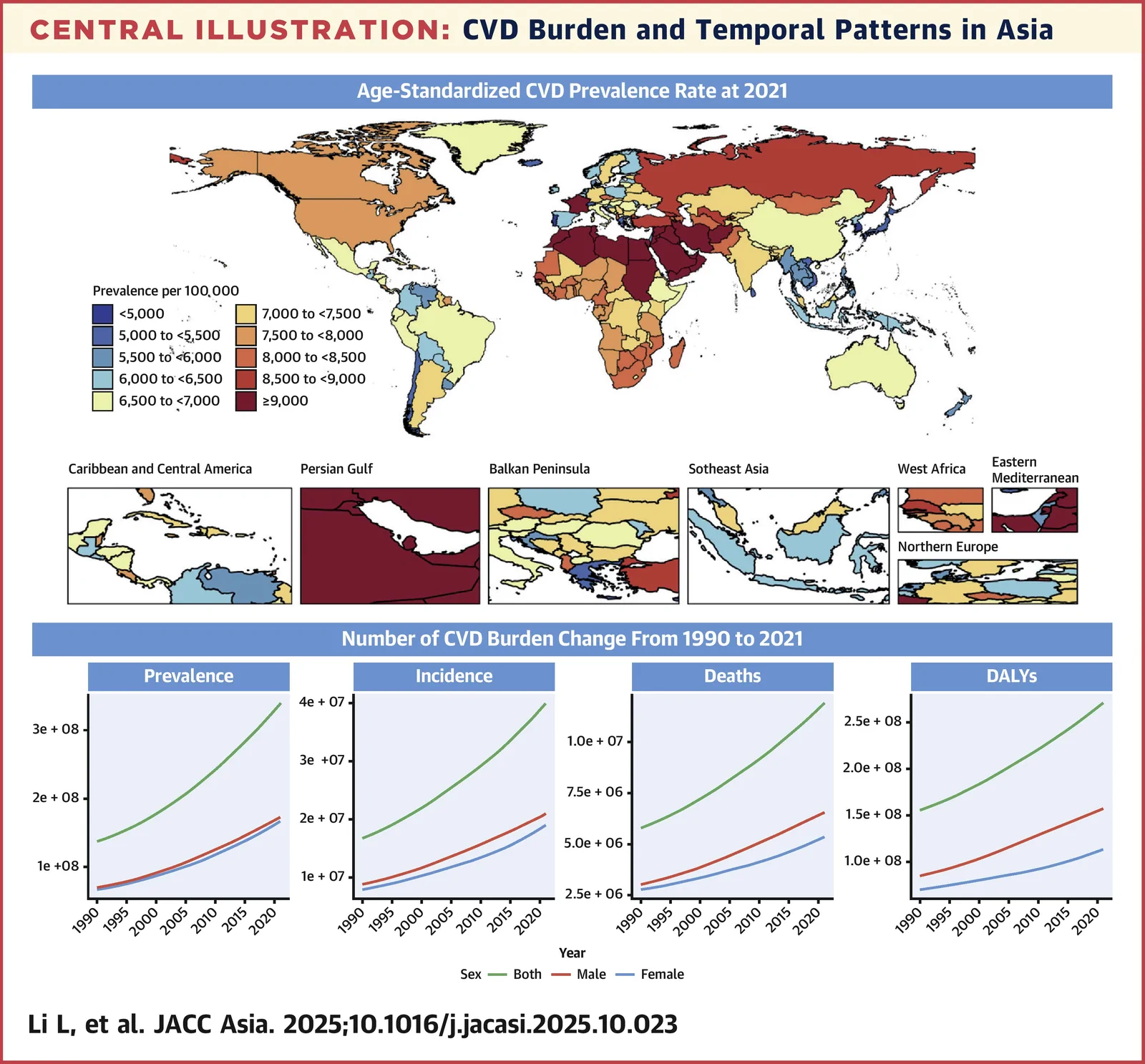

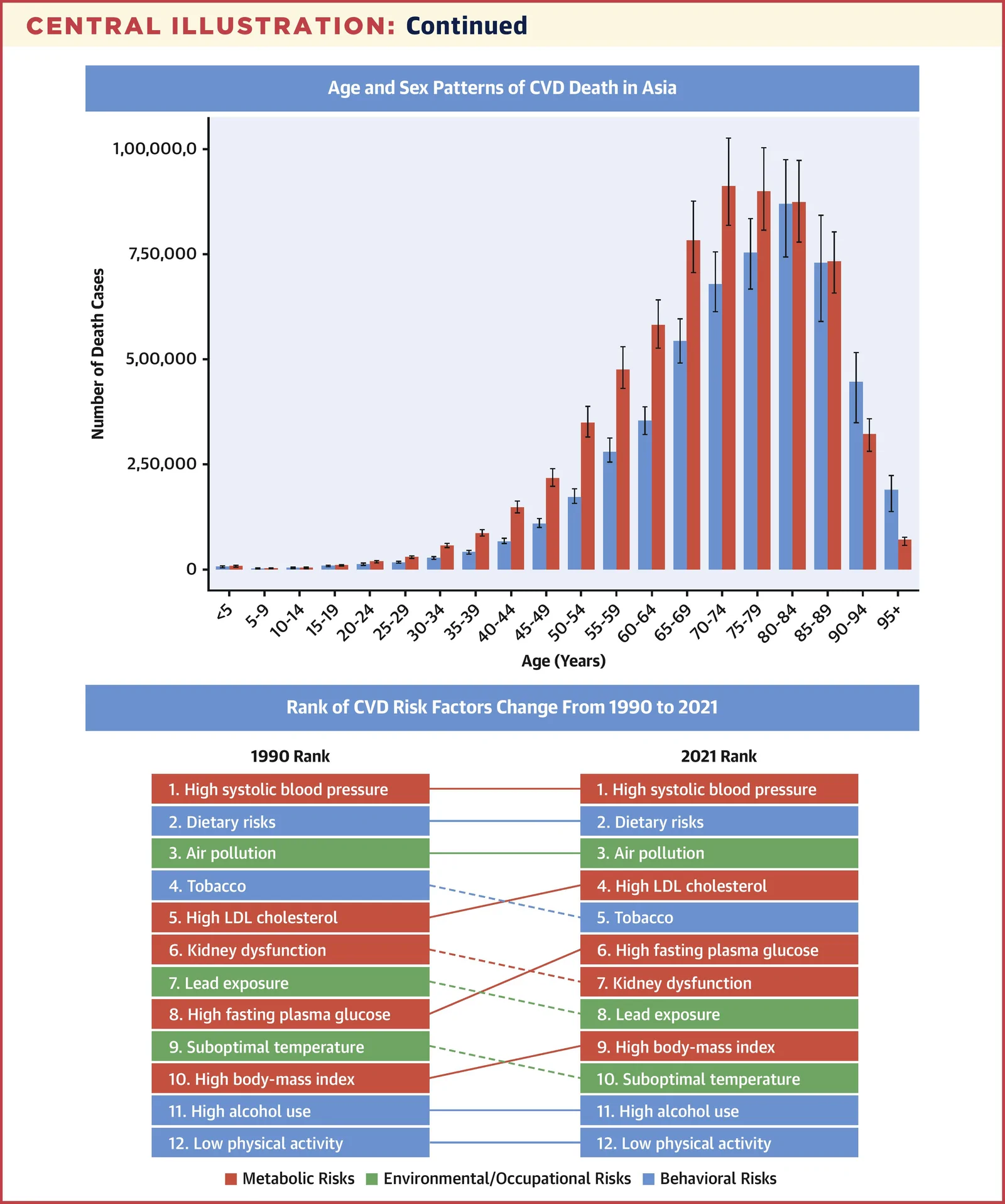
CVD Burden and Temporal Patterns in Asia From 1990 to 2021, the absolute burden of cardiovascular disease (CVD) has risen steadily, driven by demographic aging, with mortality remaining concentrated in the elderly. Throughout the period, high systolic blood pressure, dietary risks, and air pollution persisted as the leading modifiable risk factors. DALY = disability-adjusted life year; LDL = low-density lipoprotein.

The evolving epidemiological profile of CVD over the past 3 decades reflects advancements in health care and demographic shifts. Technological innovations in cardiovascular diagnostics, such as advanced imaging and artificial intelligence–assisted diagnostics, have significantly improved early detection.^17^^,^^18^ Furthermore, the implementation of national disease screening programs has led to the identification of numerous asymptomatic CVD cases, undoubtedly increasing the diagnostic and detection rates of CVD.^19^ On the other hand, the demographic structure of Asia is undergoing profound changes, with a rapidly aging population exacerbating the prevalence of age-related CVD, such as nonrheumatic valvular heart disease and atrial fibrillation.^20^ These factors have undoubtedly contributed to the rising burden of CVD prevalence. Conversely, remarkable progress has been made in the treatment of CVD in recent years, including functionally guided coronary revascularization,^21^ renal denervation for refractory hypertension,^22^ and pulsed field ablation for atrial fibrillation.^23^ Thus, even as absolute numbers of CVD cases continue to rise, age-standardized DALY and mortality rates have declined, a divergence that reflects the combined influence of demographic expansion and the expanding reach of effective screening, treatment, and secondary prevention strategies.

Gender and age disparities observed in this study merit special consideration. Men consistently demonstrated higher standardized rates for CVD prevalence, incidence, mortality, and DALYs compared with women. A recent prospective cohort study involving approximately 24,000 participants without baseline CVD found that, compared with females, males had a 49% higher relative risk of overall CVD, including a 96% increased risk of myocardial infarction, a 41% increased risk of heart failure, and a 71% increased risk of peripheral arterial disease. Additionally, males demonstrated a 43% higher relative risk of CVD-related mortality compared with females.^24^ At the cellular level, mechanistic studies have provided further evidence for these sex differences. Female cells exhibit a 20% lower rate of apoptosis, while their angiogenic capacity is 30% higher than that of males, offering a biological basis for the observed disparities in CVD burden.^25^

Our study also highlights the significant impact of age on CVD burden. As expected, older individuals bear a substantially higher burden of CVD compared with younger populations, likely due to prolonged exposure to modifiable cardiovascular risk factors such as hypertension, elevated LDL cholesterol, and diabetes.^26^ The accelerating trend of population aging has further exacerbated the CVD burden. Importantly, the reductions in CVD burden achieved through preventive measures have been—and will continue to be—partially offset by the growing aged population.^27^ Given Asia's vast population and rapid demographic aging, the region is poised to face an escalating CVD burden in the coming decades. In light of the immutable nature of biological aging, optimizing modifiable risk factors across the lifespan is critical for mitigating the future burden of CVD.

In 2021, the primary modifiable attributable risk factors for CVD in Asia were high systolic blood pressure, dietary risks, and air pollution, consistent with global trends.^28^ The impact of hypertension on CVD has been extensively validated across multiple Asian populations.^29^^,^^30^ In a previous study conducted at our center, which included 47,000 participants with a median follow-up of 11.9 years, hypertension was identified as the most significant risk factor for CVD.^31^ These findings underscore the critical importance of intensive blood pressure management in reducing the burden of CVD. Moreover, unhealthy diets, characterized by excessive salt and low fruit and vegetable intake, require public health interventions to promote healthier eating practices.^32^ Notably, the impact of air pollution on CVD in Asia has reached a level that cannot be overlooked. Across many regions of Asia, where 70% of the population resides, air pollution levels routinely exceed 35 μg/m^3^, with over 99% of individuals exposed to concentrations surpassing the annual air quality guidelines. In contrast, air pollution levels in the United States and Canada consistently average below 12 μg/m^3^.^33^ Although the mechanisms underlying air pollution–mediated systemic CVD risk are still being elucidated, numerous studies have demonstrated that exposure to air pollution significantly increases the risk of myocardial infarction,^34^ stroke,^35^ and heart failure.^36^ Therefore, important measures such as transitioning to clean fuels and reducing traffic emissions are imperative to mitigate the burden of CVD in Asia. Rapid urbanization demands coordinated action at both community and city levels. Expanding green spaces, creating walkable neighborhoods and improving public transport can all promote physical activity and lower cardiovascular risk. At the same time, primary health care systems must be strengthened by scaling up early screening for blood pressure and glucose and guaranteeing lifelong management of chronic diseases to meet the needs of an aging population. Finally, sustained collaboration across sectors is essential to tackle air pollution and unhealthy diets at their source.

In 2021, the 3 leading contributors to DALYs across Asia were IHD, stroke, and hypertensive heart disease, consistent with global patterns of CVD burden.^28^ Furthermore, we observed that the overall burden of CVD in Asia exhibited a gradual decline with increasing SDI, aligning with findings from global burden of disease studies. These studies have demonstrated that the lowest CVD burden occurs in high-SDI regions, a phenomenon attributable to improved control of risk factors and enhanced access to treatment for both acute and chronic CVD.^37^ This finding further underscores the critical importance of addressing socioeconomic disparities to reduce the burden of CVD.

Marked subregional differences persist. Central Asia continues to post some of Asia’s highest rates of CVD incidence, mortality, and DALYs, with virtually no improvement over the past 3 decades. By contrast, East Asia maintains one of the lowest regional burdens, and high-SDI nations such as Japan have achieved pronounced reductions in ASRs since 1990. These divergences likely reflect contrasting exposures to key risk factors, such as smoking prevalence, dietary patterns, hypertension control, and disparities in health care coverage.^38^

Such heterogeneity underscores the need for tailored intervention packages. Countries with a heavy or stagnating CVD burden should draw lessons from successful neighbors and strengthen the full continuum of care, from community-level prevention and early detection to timely clinical management. Priorities include expanding primary health-care capacity, ensuring early screening and treatment for high-risk groups, launching nationwide initiatives targeting dominant risk factors, and fostering cross-sector collaboration to improve environmental determinants of cardiovascular health.

### Study limitations

This study provides a comprehensive overview of CVD subtypes across multiple regions in Asia over an extended period, offering a detailed epidemiological landscape. The disaggregation of data by disease subtype, region, and risk factors enables targeted analysis. However, potential inconsistencies in data quality and reporting standards across countries may impact the accuracy of prevalence and incidence estimates. Additionally, the study focuses primarily on quantifying the burden, with limited exploration of the underlying causal mechanisms, which constrains the depth of causal inferences. Furthermore, data limitations in certain regions may create gaps in the overall analysis.

## Conclusions

The burden of CVDs in Asia has undergone significant transformations over the past 3 decades. While ASRs of incidence, mortality, and DALYs have demonstrated a declining trend, the absolute number of CVD cases has continued to increase, driven by population growth and aging. Males and older populations bear a disproportionately higher burden of CVD. Hypertension remains the most critical risk factor for CVD; however, the growing impact of air pollution on CVD burden necessitates urgent attention. The burden of CVD in Asia varies markedly across regions with different SDI levels, with high-SDI regions exhibiting a lower burden compared with low-SDI regions. This disparity highlights the imperative to address socioeconomic inequalities as a key strategy for reducing the burden of CVD.

### Data Sharing Statement

The datasets generated and/or analyzed during the current study are available at http://ghdx.healthdata.org/gbd-results-tool.

## Funding Support and Author Disclosures

This work was supported by Clinical and Translational Medicine Research Project of Chinese Academy of Medical Sciences (2022-LC04) and Noncommunicable Chronic Diseases-National Science and Technology Major Project (2024ZD0521900). The authors have reported that they have no relationships relevant to the contents of this paper to disclose.
