# Supplementary material for: Burden of Cardiovascular Disease in Asian Countries, 1990-2021
Source: JACC Asia. 2026 Feb 20;6(8):1717–30. doi: 10.1016/j.jacasi.2025.10.023 (PMC13491076; doi:10.1016/j.jacasi.2025.10.023)
Supplement: Supplementary file 1 [file mmc1.docx]

**Supplementary Materials**

**Burden of cardiovascular disease in Asian countries, 1990-2021: results from the Global Burden of Disease 2021 study**

| **Table S1. The definitions of cardiovascular diseases** | | |
| --- | --- | --- |
|  | **ICD-10 code** | **ICD-9 code** |
| Cardiovascular diseases | B33.2-B33.24, D86.85, G45-G46.8, I00.0-I01.9, I02.0, I03-I11.9, I14-I27.0, I27.2-I83.93, I86-I89.0, I89.9-I95.1, I98, I98.4-ID5.9, K75.1, R00-R01.2, R07-R07.9 | 074.2, 074.21-074.23, 391-391.9, 392.0, 393-398.99, 402-402.91, 410-416.0, 417-417.9, 420-425.5, 425.7-440.29, 440.4-445.89, 447-454.9, 456, 456.3-457, 457.1, 457.8-458.1, 459-459.9, 785-785.3, V12.5-V12.59, V15.1, V17.1, V17.3-V17.49, V42.1-V42.2, V43.2-V43.5, V45.0-V45.09, V45.81-V45.82, V47.2, V58.61, V58.63, V58.66, V58.73, V81-V81.2 |
| Rheumatic heart disease | I01-I01.9, I02.0, I05-I09.9 | 391-391.9, 392.0, 393-398.99 |
| Ischemic heart disease | I20-I21.6, I21.9-I25.9 | 410-414.9, V17.3 |
| Stroke | G45-G46.8, I60-I62, I62.9-I64, I64.1, I65-I69.998 | 430-439.6, V12.54, V17.1 |
| Hypertensive heart disease | I11-I11.2, I11.9 | 402-402.91 |
| Non-rheumatic valvular heart disease | I34-I37.9 | 424.0-424.3 |
| Cardiomyopathy and myocarditis | B33.2-B33.20, B33.22-B33.24, D86.85, I40-I41.8,  I42-I43.8, I51.4-I51.6 | 074.2, 074.23, 422-422.99, 425-425.5, 425.7-425.9, 429.0-  429.1 |
| Pulmonary Arterial Hypertension | \ | 416.0 |
| Atrial fibrillation and flutter | I48-I48.92 | 427.3-427.32 |
| Lower extremity peripheral arterial disease | I70.2-I70.92, I73-I73.9 | 440.2-440.29, 440.4-440.9, 443-443.2, 443.8-443.9 |
| Endocarditis | B33.21, I33-I33.9, I38-I38.0, I39-I39.9 | 074.22, 421-421.9, 424, 424.4-424.99 |
| Other cardiovascular and circulatory diseases | I30-I32.8, I51-I51.3, I51.7-I52.8, I62.0-I62.1, I72-  I72.9, I77-I83.93, I86-I89.0, I89.9, I95.0-I95.1, I98,  I98.8-I99.9, K75.1 | 074.21, 417-417.9, 420-420.99, 423-423.9, 429, 429.2-  429.9, 442-442.9, 443.21-443.29, 447-454.9, 456, 456.3-  457, 457.1, 457.8-458.1, 459-459.9 |

| **Table S2. Age-Standardized Rates (per 100,000) and Absolute Number (1,000s) of DALYs, Death, Prevalence, and Incidence by Cardiovascular Diseases Category in Asia, 1990–2021** | | | | | | | |
| --- | --- | --- | --- | --- | --- | --- | --- |
|  | **ASRs (per 100,000) (95% UI)** | | |  | **Absolute number (100,000s) (95% UI)** | | |
|  | **1990** | **2021** | **Percentage**  **Change, 1990 to 2021** |  | **1990** | **2021** | **Percentage**  **Change, 1990 to 2021** |
| **Cardiovascular diseases** | | | | | | | |
| Prevalence | 6416.1  (5994.4 to 6822.4) | 6782.6  (6292.4 to 7301.6) | 5.7%  (3.9 to 8.1) |  | 1362.0  (1263.5 to 1450.0) | 3404.1  (3151.3 to 3669.2) | 149.9%  (143.7 to 157.4) |
| Incidence | 820.3  (746.6 to 900.1) | 806.1  (728.8 to 887.6) | -1.7%  (-3.0 to -0.5) |  | 167.0  (151.5 to 183.5) | 399.0  (358.9 to 440.5) | 138.9%  (133.6 to 144.5) |
| Deaths | 347.7  (321.8 to 369.2) | 257.4  (233.2 to 279.6) | -26.0%  (-32.6 to -18.4) |  | 57.7  (53.9 to 61.4) | 119.0  (108.5 to 129.1) | 106.1%  (86.3 to 127.9) |
| DALYs | 7553.3  (7090.2 to 8010.5) | 5476.7  (5096.4 to 5865.4) | -27.5%  (-33.4 to -20.8) |  | 1549.8  (1456.5 to 1643.8) | 2704.2  (2515.6 to 2901.6) | 74.5%  (59.8 to 90.6) |
| **Rheumatic heart disease** | | | | | | | |
| Prevalence | 610.1  (482.9 to 748.1) | 609.4  (476.2 to 753.9) | -0.1%  (-2.1 to 1.7) |  | 195.8  (152.3 to 244.2) | 293.4  (231.3 to 362.1) | 49.9%  (43.2 to 56.1) |
| Incidence | 42.8  (34.2 to 52.9) | 42.7  (33.9 to 53.1) | -0.3%  (-2.3 to 1.7) |  | 15.2  (12.0 to 19.1) | 18.8  (15.0 to 23.2) | 23.2%  (19.4 to 27.4) |
| Deaths | 16.4  (13.3 to 19.8) | 6.6  (5.6 to 8.0) | -59.8%  (-68.2 to -50.2) |  | 3.3  (2.7 to 4.1) | 3.2  (2.7 to 3.9) | -5.4%  (-23.7 to 17.3) |
| DALYs | 501.9  (411.0 to 608.7) | 217.2  (186.6 to 256.8) | -56.7%  (-64.0 to -48.1) |  | 128.3  (106.2 to 154.7) | 107.1  (91.8 to 126.9) | 16.5%  (-29.8 to -1.0) |
| **Ischemic heart disease** | | | | | | | |
| Prevalence | 2884.2  (2523.4 to 3276.9) | 3223.6  (2785.2 to 3816.6) | 11.8%  (7.3 to 17.3) |  | 554.6  (483.1 to 631.6) | 1624.1  (1398.9 to 1913.5) | 192.8%  (180.9 to 206.4) |
| Incidence | 389.4  (317.4 to 470.0) | 406.6  (331.2 to 486.2) | 4.4%  (3 to 5.9) |  | 74.1  (59.8 to 90.4) | 202.4  (164.6 to 245.4) | 173%  (165.5 to 182.5) |
| Deaths | 117.0  (108.6 to 124.7) | 113.3  (102.6 to 122.4) | -3.1%  (-11.1 to 5.4) |  | 19.4  (18.1 to 20.6) | 52.6  (48 to 56.8) | 131.3%  (112.3 to 152.7) |
| DALYs | 2476  (2322.6 to 2631.1) | 2340.9  (2169.7 to 2515.9) | -5.5%  (-13.0 to 2.7) |  | 503.6  (471.6 to 535.2) | 1164.9  (1080.1 to 1249.2) | 192.8%  (180.9 to 206.4) |
| **Stroke** | | | | | | | |
| Prevalence | 1164.9  (1092.9 to 1248.2) | 1137.0  (1069.5 to 1212.6) | -2.4%  (-4 to -0.9) |  | 262.0  (246.9 to 278.7) | 573.3  (538.4 to 612.3) | 118.8%  (114.0 to 124.1) |
| Incidence | 194.9  (176.3 to 215.5) | 162.7  (146.6 to 180.4) | -16.6%  (-19.6 to -13.4) |  | 37.7  (34.1 to 42.1) | 79.5  (71.4 to 88.7) | 110.8%  (101.9 to 119.6) |
| Deaths | 172.8  (157.4 to 187.2) | 108.2  (96.1 to 118.5) | -37.4%  (-44 to -29.2) |  | 28.6  (26.3 to 30.9) | 50.3  (45.0 to 55.1) | 75.6%  (56.5 to 98.6) |
| DALYs | 3687.5  (3401.8 to 3977.7) | 2280.1  (2054.1 to 2472.5) | -38.2%  (-44.4 to -30.8) |  | 740.4  (687.4 to 796.5) | 1128.7  (1020.2 to 1228.3) | 52.4%  (36.7 to 70.9) |
| **Hypertensive heart disease** | | | | | | | |
| Prevalence | 155  (123.1 to 192.9) | 150.8  (117.7 to 190.6) | -2.7%  (-9.9 to 4.4) |  | 26.6  (21 to 33.4) | 72.7  (56.8 to 92.4) | 172.8%  (150.8 to 192.8) |
| Deaths | 28.1  (21.7 to 32.1) | 16.7  (13.1 to 19.7) | -40.5%  (-51.5 to -19.8) |  | 4.3  (3.2 to 4.9) | 7.5 (5.9 to 8.8) | 76.2%  (44.6 to 139.7) |
| DALYs | 515.0  (391.1 to 585.3) | 300.4  (238.0 to 346.9) | -41.7%  (-51.2 to -21.3) |  | 94.1  (69.1 to 107.9) | 145.2  (114.8 to 167.9) | 54.4%  (28.5 to 111.3) |
| **Non-rheumatic valvular heart disease** | | | | | | | |
| Prevalence | 170.0  (156.1 to 184.9) | 189.5  (176.2 to 203.1) | 11.4%  (8.6 to 14.3) |  | 29.7  (27.4 to 32.4) | 91.0  (84.6 to 97.7) | 206.6%  (197.5 to 216.3) |
| Incidence | 12.9  (11.9 to 14.0) | 13.9  (12.9 to 14.9) | 8.1%  (5.2 to 11.0) |  | 2.7  (2.5 to 2.9) | 7.3  (6.8 to 7.9) | 172.4%  (164.5 to 180.8) |
| Deaths | 1.4  (1.2 to 1.6) | 1.2  (1.0 to 1.4) | -11.3%  (-21.7 to -2.5) |  | 0.2  (0.2 to 0.2) | 0.5  (0.4 to 0.6) | 164.2%  (127.2 to 199.1) |
| DALYs | 26.1  (22.9 to 30.3) | 21.8  (19.1 to 24.8) | -16.7%  (-25 to -8.3) |  | 4.7  (4 to 5.5) | 9.8  (8.6 to 11.3) | 111.1%  (83.6 to 140.6) |
| **Cardiomyopathy and myocarditis** | | | | | | | |
| Prevalence | 33.0  (28.1 to 37.4) | 41.9  (34.9 to 49) | 27.1%  (19.9 to 34.7) |  | 8.7  (7.4 to 10.2) | 19.0  (15.6 to 22.2) | 117.3%  (102 to 134) |
| Incidence | 17.0  (13.6 to 20.9) | 16.4  (13.2 to 20.0) | -3.5%  (-4.5 to -2.3) |  | 0.7  (0.6 to 0.9) | 1.5  (1.3 to 1.7) | 107.3%  (68.8 to 159.5) |
| Deaths | 3.9  (3.0 to 4.8) | 3.2  (2.9 to 3.6) | -17.7%  (-31.0 to 1.4) |  | 0.7  (0.6 to 0.9) | 1.5  (1.3 to 1.7) | 107.3%  (68.8 to 159.5) |
| DALYs | 110.9  (86.0 to 134.2) | 92.4  (83.0 to 104.1) | -16.7%  (-30.8 to 2.5) |  | 28.7  (22.2 to 34.9) | 43.4  (39.1 to 49.1) | 51.3%  (19.9 to 88.4) |
| **Pulmonary arterial hypertension** | | | | | | | |
| Prevalence | 2.0  (1.6 to 2.5) | 2.0  (1.7 to 2.5) | 1.9%  (1.1 to 2.7) |  | 0.5  (0.4 to 0.6) | 1.0  (0.8 to 1.3) | 97.5%  (85.3 to 109.7) |
| Incidence | 0.5  (0.4 to 0.6) | 0.5  (0.4 to 0.6) | 2%  (1.3 to 2.6) |  | 0.1  (0.1 to 0.2) | 0.3  (0.2 to 0.3) | 96.9%  (85.7 to 107.6) |
| Deaths | 0.4  (0.3 to 0.5) | 0.3  (0.2 to 0.4) | -21.8%  (-43.1 to 4.7) |  | 0.1  (0.1 to 0.1) | 0.1  (0.1 to 0.2) | 67.4%  (23.9 to 119.6) |
| DALYs | 13.4  (10.4 to 16.5) | 8.7  (6.8 to 10.4) | -34.7%  (-49.7 to -17.4) |  | 3.8  (2.9 to 4.7) | 3.9  (3 to 4.6) | 0.7%  (-27.1 to 32) |
| **Atrial fibrillation and flutter** | | | | | | | |
| Prevalence | 507.6  (401 to 652.6) | 529  (420.3 to 680.9) | 4.2%  (2.4 to 6.0) |  | 87.4  (69.3 to 114) | 257.4  (204.3 to 335.9) | 194.6%  (188 to 202) |
| Incidence | 46.3  (35.3 to 61.4) | 46.7  (35.9 to 61.7) | 0.9%  (-1.1 to 2.6) |  | 8.5  (6.6 to 11.1) | 23.2  (18 to 30.5) | 173.5%  (164.7 to 182.2) |
| Deaths | 3.6  (3.0 to 4.5) | 3.8  (3.2 to 4.3) | 5.3%  (-14.5 to 27.6) |  | 0.4  (0.3 to 0.5) | 1.5  (1.3 to 1.7) | 289.6%  (215.9 to 377.3) |
| DALYs | 85.1  (68.7 to 106.2) | 88.0  (72.8 to 107.3) | 3.4%  (-8.5 to 15.4) |  | 13.0  (10.2 to 16.3) | 40.3  (32.7 to 49.8) | 210.7%  (179.1 to 247.7) |
| **Aortic aneurysm** | | | | | | | |
| Deaths | 1.0  (0.9 to 1.2) | 1.4  (1.2 to 1.6) | 39.5%  (20.3 to 59.6) |  | 0.2  (0.1 to 0.2) | 0.6  (0.5 to 0.7) | 286.9%  (228 to 349.3) |
| DALYs | 19.7  (16.8 to 23.9) | 25.7  (22.9 to 29.3) | 30.6%  (9.2 to 53.1) |  | 3.9  (3.3 to 4.8) | 12.5  (11.2 to 14.2) | 220.7%  (163.9 to 282.9) |
| **Lower extremity peripheral arterial disease** | | | | | | | |
| Prevalence | 1208.7  (1044.3 to 1397.5) | 1196.4  (1032.7 to 1382.8) | -1.0%  (-1.9 to -0.2) |  | 225.8  (193.2 to 263.3) | 600.0  (516.0 to 695.0) | 165.8%  (162.2 to 170.1) |
| Incidence | 107.6  (93.1 to 124.2) | 105.1  (90.7 to 121.6) | -2.4%  (-3.2 to -1.5) |  | 21.6  (18.6 to 25.2) | 54.1  (46.7 to 63.2) | 150.5%  (146.6 to 154.6) |
| Deaths | 0.2  (0.2 to 0.3) | 0.2  (0.2 to 0.3) | 5.9%  (-9.6 to 19.7) |  | 0.0  (0.0 to 0.0) | 0.1  (0.1 to 0.1) | 266.2%  (204.8 to 327.1) |
| DALYs | 9.8  (6.2 to 16.1) | 9.2  (6.1 to 14.7) | -6.2%  (-12.5 to 1.6) |  | 1.6  (1.0 to 2.7) | 4.4  (2.9 to 7.1) | 171.4%  (150.8 to 197.2) |
| **Endocarditis** | | | | | | | |
| Prevalence | 2.1  (1.9 to 2.3) | 3.4  (3 to 3.8) | 62.6%  (55.4 to 70.3) |  | 0.6  (0.5 to 0.6) | 1.5  (1.4 to 1.7) | 170.1%  (151.2 to 190.8) |
| Incidence | 8.9  (7.6 to 10.7) | 11.6  (9.9 to 13.6) | 29.7%  (20.8 to 39.8) |  | 2.5  (2.1 to 3.0) | 5.6  (4.7 to 6.6) | 129.4%  (103.8 to 160.3) |
| Deaths | 0.8  (0.6 to 0.9) | 0.7  (0.6 to 0.8) | -15.4%  (-27.9 to -1.6) |  | 0.2  (0.1 to 0.2) | 0.3  (0.3 to 0.4) | 85.8%  (56 to 120.8) |
| DALYs | 24.7  (18.9 to 29.0) | 18.3  (15.9 to 23.1) | -25.7%  (-37.4 to -10.7) |  | 6.7  (5.0 to 8.0) | 8.6  (7.5 to 10.9) | 28.2%  (6.1 to 62.0) |
| **Other cardiovascular and circulatory diseases** | | | | | | | |
| Prevalence | 429.4  (339.6 to 541.9) | 550.8  (425.6 to 695.8) | 28.3%  (19.4 to 37.2) |  | 90.0  (71.7 to 111.3) | 271.0  (205.4 to 347.0) | 201%  (177.9 to 222.5) |
| Deaths | 2.1  (1.8 to 2.7) | 1.7  (1.5 to 1.9) | -20.8%  (-38.4 to -5.4) |  | 0.4  (0.3 to 0.5) | 0.8  (0.7 to 0.9) | 89.9%  (55.6 to 133.7) |
| DALYs | 83.3  (70.1 to 96.6) | 74  (62.8 to 89.2) | -11.2%  (-22.1 to 4) |  | 21.0  (17.0 to 24.7) | 35.3  (29.6 to 43) | 68.2%  (43.5 to 108.6) |

ASR: Age-standardized rate; DALYs: disability-adjusted life years; UI: uncertainty interval

| **Table S3. Age-Standardized Rates (per 100,000) and Absolute Number (1,000s) of DALYs, Death, Prevalence, and Incidence by Cardiovascular Diseases Category in Asia, 2019–2021** | | | | | | | |
| --- | --- | --- | --- | --- | --- | --- | --- |
|  | **ASRs (per 100,000) (95% UI)** | | |  | **Absolute number (100,000s) (95% UI)** | | |
|  | **2019** | **2021** | **Percentage**  **Change, 2019 to 2021** |  | **2019** | **2021** | **Percentage**  **Change, 2019 to 2021** |
| **Cardiovascular diseases** | | | | | | | |
| Prevalence | 6721.3  (6274.8 to 7171.1) | 6782.6  (6292.4 to 7301.6) | 0.9%  (-0.6 to 2.8) |  | 3202.3  (2987.1 to 3418.4) | 3404.1  (3151.3 to 3669.2) | 6.3%  (4.7 to 8.4) |
| Incidence | 808.9  (739.2 to 883.4) | 806.1  (728.8 to 887.6) | -0.3%  (-2.1 to 1.7) |  | 379.5  (345.7 to 414.9) | 399.0  (358.9 to 440.5) | 5.1%  (3.2 to 7.2) |
| Deaths | 262.7  (239.2 to 281.6) | 257.4  (233.2 to 279.6) | -2.0%  (-10.1 to 7.2) |  | 114.0  (104.7 to 122) | 119.0  (108.5 to 129.1) | 4.4%  (-4.3 to 14.3) |
| DALYs | 5587.8  (5172.4 to 5956.3) | 5476.7  (5096.4 to 5865.4) | -2.0%  (-9.3 to 6.6) |  | 2615.2  (2432.5 to 2787.5) | 2704.2  (2515.6 to 2901.6) | 3.4%  (-4.5 to 12.7) |
| **Rheumatic heart disease** | | | | | | | |
| Prevalence | 607.5  (475.8 to 750.5) | 609.4  (476.2 to 753.9) | 0.3%  (-0.7 to 1.2) |  | 288.2  (226.9 to 354.2) | 293.4  (231.3 to 362.1) | 1.8%  (0.9 to 2.7) |
| Incidence | 42.5  (33.7 to 52.8) | 42.7  (33.9 to 53.1) | 0.5%  (-0.6 to 1.6) |  | 18.6  (14.8 to 23.0) | 18.8  (15.0 to 23.2) | 1.1%  (-0.2 to 2.2) |
| Deaths | 7.0  (6.0 to 8.6) | 6.6  (5.6 to 8.0) | -6.0%  (-13.4 to 3.3) |  | 3.2  (2.8 to 3.9) | 3.2  (2.7 to 3.9) | -1.0%  (-8.9 to 9.0) |
| DALYs | 227.9  (198.2 to 273.5) | 217.2  (186.6 to 256.8) | -4.7%  (-11 to 2.9) |  | 108.5  (94 to 130.7) | 107.1  (91.8 to 126.9) | -1.3%  (-7.9 to 6.7) |
| **Ischemic heart disease** | | | | | | | |
| Prevalence | 3182.4  (2792.1 to 3608.3) | 3223.6  (2785.2 to 3816.6) | 1.3% (-2.5 to 5.7) |  | 1512.5  (1322.8 to 1723.0) | 1624.1  (1398.9 to 1913.5) | 7.4%  (3.3 to 12.2) |
| Incidence | 406.7  (343.1 to 478.6) | 406.6  (331.2 to 486.2) | 0.0% (-3.8 to 3.9) |  | 191.4  (160.2 to 227.1) | 202.4  (164.6 to 245.4) | 5.7%  (1.6 to 9.9) |
| Deaths | 114.9  (105.3 to 122.9) | 113.3  (102.6 to 122.4) | -1.4% (-8.7 to 7.0) |  | 50.1  (46.2 to 53.4) | 52.6  (48 to 56.8) | 5.0%  (-2.9 to 14.1) |
| DALYs | 2372.9  (2209.8 to 2528) | 2340.9  (2169.7 to 2515.9) | -1.3% (-8.6 to 7.2) |  | 1120.0  (1045.7 to 1190.5) | 1164.9  (1080.1 to 1249.2) | 4.0%  (-3.8 to 13.2) |
| **Stroke** | | | | | | | |
| Prevalence | 1122.2  (1060.8 to 1189.8 | 1137.0  (1069.5 to 1212.6) | 1.3%  (-0.4 to 3.1) |  | 538.6  (509.2 to 570.9) | 573.3  (538.4 to 612.3) | 6.4%  (4.6 to 8.4) |
| Incidence | 166.0  (150.6 to 182.4) | 162.7  (146.6 to 180.4) | -2.0%  (-3.2 to -0.6) |  | 76.8  (69.4 to 84.6) | 79.5  (71.4 to 88.7) | 3.6%  (2.3 to 5.3) |
| Deaths | 110.6  (99.3 to 120.4) | 108.2  (96.1 to 118.5) | -2.1%  (-11.9 to 8.8) |  | 48.2  (43.6 to 52.4) | 50.3  (45.0 to 55.1) | 4.3%  (-6.3 to 16.2) |
| DALYs | 2331.9  (2123.6 to 2520.6) | 2280.1  (2054.1 to 2472.5) | -2.2%  (-11.3 to 8.1) |  | 1091.8  (995.8 to 1182.2) | 1128.7  (1020.2 to 1228.3) | 3.4%  (-6.3 to 14.6) |
| **Hypertensive heart disease** | | | | | | | |
| Prevalence | 146.7  (117.6 to 181.3) | 150.8  (117.7 to 190.6) | 2.8%  (-3.9 to 8.1) |  | 66.4  (53.3 to 82.1) | 72.7  (56.8 to 92.4) | 9.5%  (2.3 to 15.5) |
| Deaths | 17.3  (13.4 to 20.0) | 16.7  (13.1 to 19.7) | -3.2%  (-11.1 to 5.3) |  | 7.2  (5.6 to 8.4) | 7.5 (5.9 to 8.8) | 3.6%  (-5.0 to 12.8) |
| DALYs | 309.3  (240.9 to 355.1) | 300.4  (238.0 to 346.9) | -2.9%  (-10.6 to 5.3) |  | 140.8  (109.8 to 161.5) | 145.2  (114.8 to 167.9) | 3.1%  (-5.2 to 12.0) |
| **Non-rheumatic valvular heart disease** | | | | | | | |
| Prevalence | 170.0  (156.1 to 184.9) | 189.5  (176.2 to 203.1) | -1.4%  (-2.1 to -0.8) |  | 86.4  (80.5 to 92.8) | 91.0  (84.6 to 97.7) | 5.3%  (4.6 to 6.0) |
| Incidence | 14.1  (13.2 to 15.2) | 13.9  (12.9 to 14.9) | -1.7%  (-2.5 to -1.0) |  | 7.0  (6.5 to 7.5) | 7.3  (6.8 to 7.9) | 4.6%  (3.8 to 5.4) |
| Deaths | 1.3  (1.1 to 1.4) | 1.2  (1.0 to 1.4) | -2.8%  (-5.2 to -0.4) |  | 0.5  (0.4 to 0.5) | 0.5  (0.4 to 0.6) | 5.0%  (1.8 to 8.1) |
| DALYs | 22.2  (19.5 to 25.4) | 21.8  (19.1 to 24.8) | -2.1%  (-5.2 to 1.2) |  | 9.4  (8.3 to 10.8) | 9.8  (8.6 to 11.3) | 4.1%  (0.3 to 7.9) |
| **Cardiomyopathy and myocarditis** | | | | | | | |
| Prevalence | 41.7  (35.5 to 47.8) | 41.9  (34.9 to 49) | 0.7%  (-3.4 to 5.0) |  | 18.4  (15.6 to 21.1) | 19.0  (15.6 to 22.2) | 3.3%  (-1.0 to 7.6) |
| Incidence | 16.4  (13.2 to 20.0) | 16.4  (13.2 to 20.0) | 0.0%  (-0.1 to 0.0) |  | 7.5  (6.1 to 9.3) | 1.5  (1.3 to 1.7) | 3.3%  (2.7 to 3.9) |
| Deaths | 3.4  (3.0 to 3.8) | 3.2  (2.9 to 3.6) | -4.4%  (-9.6 to 1.7) |  | 1.5  (1.3 to 1.7) | 1.5  (1.3 to 1.7) | 1.2%  (-4.5 to 8.0) |
| DALYs | 96.7  (86.3 to 108.5) | 92.4  (83.0 to 104.1) | -4.5%  (-9.2 to 1.3) |  | 43.8  (39.2 to 49.3) | 43.4  (39.1 to 49.1) | -0.9%  (-6.2 to 5.3) |
| **Pulmonary arterial hypertension** | | | | | | | |
| Prevalence | 2.1  (1.7 to 2.6) | 2.0  (1.7 to 2.5) | 1.9%  (-2.6 to -1.3) |  | 1.0  (0.8 to 1.3) | 1.0  (0.8 to 1.3) | 1.7%  (0.8 to 2.6) |
| Incidence | 0.5  (0.4 to 0.6) | 0.5  (0.4 to 0.6) | 1.2%  (0.6 to 1.7) |  | 0.2  (0.2 to 0.3) | 0.3  (0.2 to 0.3) | 4.9%  (4.1 to 5.7) |
| Deaths | 0.3  (0.2 to 0.4) | 0.3  (0.2 to 0.4) | -4.3%  (-13.0 to 6.5) |  | 0.1  (0.1 to 0.2) | 0.1  (0.1 to 0.2) | 0.9%  (-8.7 to 13.1) |
| DALYs | 9.3  (7.4 to 11.0) | 8.7  (6.8 to 10.4) | -6.0%  (-12.6 to 2.1) |  | 4.0  (3.1 to 4.7) | 3.9  (3 to 4.6) | -3.2%  (-10.4 to 6.1) |
| **Atrial fibrillation and flutter** | | | | | | | |
| Prevalence | 522.0  (416.7 to 669.1) | 529.0  (420.3 to 680.9) | 1.4%  (0.4 to 2.3) |  | 238.5  (190.6 to 309.1) | 257.4  (204.3 to 335.9) | 7.9%  (6.9 to 9.0) |
| Incidence | 46.2  (35.6 to 61.0) | 46.7  (35.9 to 61.7) | 1.0%  (0.2 to 1.8) |  | 21.6  (16.8 to 28.3) | 23.2  (18 to 30.5) | 7.3%  (6.2 to 8.3) |
| Deaths | 3.8  (3.2 to 4.3) | 3.8  (3.2 to 4.3) | -0.3%  (-7.4 to 8.0) |  | 1.4  (1.2 to 1.6) | 1.5  (1.3 to 1.7) | 7.7%  (-0.1 to 16.8) |
| DALYs | 87.7  (71.6 to 105.9) | 88.0  (72.8 to 107.3) | 0.3%  (-3.6 to 4.9) |  | 37.6  (30.3 to 46.2) | 40.3  (32.7 to 49.8) | 7.2%  (3.2 to 11.8) |
| **Aortic aneurysm** | | | | | | | |
| Deaths | 1.4  (1.2 to 1.6) | 1.4  (1.2 to 1.6) | -2.1%  (-5.9 to 1.6) |  | 0.6  (0.5 to 0.7) | 0.6  (0.5 to 0.7) | 4.7%  (0.1 to 9.1) |
| DALYs | 26.2  (23.4 to 29.9) | 25.7  (22.9 to 29.3) | -1.9%  (-7.2 to 3.5) |  | 12.0  (10.8 to 13.8) | 12.5  (11.2 to 14.2) | 3.8%  (-2.3 to 9.9) |
| **Lower extremity peripheral arterial disease** | | | | | | | |
| Prevalence | 1191.8  (1030.2 to 1378.9) | 1196.4  (1032.7 to 1382.8) | 0.4%  (-0.1 to 0.9) |  | 563.0  (484.8 to 651.2) | 600.0  (516.0 to 695.0) | 6.6%  (6.0 to 7.1) |
| Incidence | 104.9  (90.6 to 121.2) | 105.1  (90.7 to 121.6) | 0.1%  (-0.4 to 0.6) |  | 51.2  (44.1 to 59.7) | 54.1  (46.7 to 63.2) | 5.8%  (5.1 to 6.5) |
| Deaths | 0.2  (0.2 to 0.3) | 0.2  (0.2 to 0.3) | -1.3%  (-6.1 to 3.5) |  | 0.1  (0.1 to 0.1) | 0.1  (0.1 to 0.1) | 6.6%  (0.8 to 12.4) |
| DALYs | 9.2  (6.2 to 14.8) | 9.2  (6.1 to 14.7) | -0.4%  (-2.8 to 2.0) |  | 4.1  (2.7 to 6.7) | 4.4  (2.9 to 7.1) | 6.6%  (3.8 to 9.3) |
| **Endocarditis** | | | | | | | |
| Prevalence | 3.4  (3.0 to 3.8) | 3.4  (3.0 to 3.8) | 0.8%  (-1.9 to 3.5) |  | 1.5  (1.3 to 1.6) | 1.5  (1.4 to 1.7) | 4.6%  (2.0 to 7.4) |
| Incidence | 11.4  (9.7 to 13.4) | 11.6  (9.9 to 13.6) | 1.6%  (0.7 to 2.4) |  | 5.3  (4.5 to 6.2) | 5.6  (4.7 to 6.6) | 6.3%  (5.2 to 7.3) |
| Deaths | 0.7  (0.6 to 0.8) | 0.7  (0.6 to 0.8) | -3.4%  (-7.1 to 0.4) |  | 0.3  (0.3 to 0.4) | 0.3  (0.3 to 0.4) | 2.0%  (-2.3 to 6.4) |
| DALYs | 19.0  (16.5 to 23.3) | 18.3  (15.9 to 23.1) | -3.4%  (-7.5 to 1.4) |  | 8.6  (7.5 to 10.7) | 8.6  (7.5 to 10.9) | -0.1%  (-4.6 to 5.0) |
| **Other cardiovascular and circulatory diseases** | | | | | | | |
| Prevalence | 541.2  (428.3 to 665.1) | 550.8  (425.6 to 695.8) | 1.8%  (-4.6 to 7.4) |  | 253.8  (199.9 to 315.1) | 271.0  (205.4 to 347.0) | 6.8%  (-0.2 to 12.8) |
| Deaths | 1.7  (1.5 to 2.0) | 1.7  (1.5 to 1.9) | -3.5%  (-8.3 to 1.5) |  | 0.8  (0.7 to 0.9) | 0.8  (0.7 to 0.9) | 2.0%  (-3.4 to 7.7) |
| DALYs | 75.5  (64.7 to 89.7) | 74  (62.8 to 89.2) | -1.9%  (-5.4 to 1.9) |  | 34.6  (29.5 to 41.3) | 35.3  (29.6 to 43) | 2.2%  (-1.7 to 6.6) |

ASR: Age-standardized rate; DALYs: disability-adjusted life years; UI: uncertainty interval

| **Table S4. Cardiovascular diseases burden by country.** | | | | | | | | |
| --- | --- | --- | --- | --- | --- | --- | --- | --- |
|  | **Prevalence (95%UI)** | | **Incidence (95%UI)** | | **Death (95%UI)** | | **DALYs (95%UI)** | |
| **Location** | **ASR (per 100,000) 2021** | **Percent change, 1990-2021** | **ASR (per 100,000) 2021** | **Percent change, 1990-2021** | **ASR (per 100,000) 2021** | **Percent change, 1990-2021** | **ASR (per 100,000) 2021** | **Percent change, 1990-2021** |
| **Central Asia** | **8144.0 (7697.7 to 8542.4)** | **5.1 (3.5 to 6.6)** | **1280.2 (1202.2 to 1376.2)** | **14.3 (11.6 to 17.0)** | **436.1 (395.7 to 474.5)** | **-15.0 (-21.7 to -8.0)** | **8630.7 (7862.7 to 9433.9)** | **-19.1 (-25.8 to -12.2)** |
| Kazakhstan | 7465.3 (7035.1 to 7841.7) | -3.5 (-6.2 to -0.9) | 1032.1 (951.4 to 1122.2) | -7.9 (-11.1 to -4.7) | 461.4 (420.3 to 501.6) | -8.9 (-17.0 to -0.7) | 8782.2 (7973.4 to 9566.8) | -16.3 (-24.4 to -8.4) |
| Kyrgyzstan | 7164.3 (6753.0 to 7555.8) | -7.5 (-9.7 to -5.4) | 1005.4 (914.6 to 1110.5) | -7.2 (-10.9 to -3.6) | 404.2 (344.3 to 463.4) | -17.9 (-29.5 to -5.1) | 8003.5 (6837.3 to 9209.8) | -23.2 (-34.8 to -10.5) |
| Tajikistan | 7463.5 (7038.0 to 7906.7) | 1.7 (-0.7 to 4.1) | 1249.2 (1150.4 to 1357.4) | 20.9 (14.7 to 27.1) | 414.5 (356.4 to 467.1) | -20.2 (-31.1 to -9.3) | 7891.0 (6832.9 to 8938.1) | -26.2 (-36.3 to -15.2) |
| Turkmenistan | 8446.1 (7988.8 to 8882.7) | 6.5 (3.9 to 9.1) | 1178.0 (1075.5 to 1286.1) | -0.2 (-3.9 to 3.8) | 552.2 (453.6 to 672.8) | -6.4 (-23.5 to 14.8) | 11751.3 (9600.2 to 14451.2) | -6.5 (-23.8 to 15.5) |
| Uzbekistan | 8783.8 (8317.7 to 9210.5) | 18.4 (15.7 to 20.9) | 1721.9 (1633.5 to 1826.6) | 65.2 (56.6 to 75.2) | 478.5 (417.3 to 539.4) | 0.7 (-11.6 to 13.1) | 9428.7 (8285.0 to 10587.0) | -6.2 (-17.9 to 5.9) |
| **East Asia** | **6601.7 (6123.7 to 7076.6)** | **9.2 (6.9 to 12.1)** | **809.3 (734.4 to 888.7)** | **3.0 (0.8 to 5.2)** | **276.2 (235.1 to 318.3)** | **-31.5 (-42.4 to -18.1)** | **5093.3 (4333.6 to 5873.2)** | **-36.5 (-46.5 to -23.7)** |
| China | 6603.7 (6121.9 to 7087.6) | 9.6 (7.3 to 12.7) | 811.8 (736.1 to 892.0) | 3.6 (1.3 to 5.8) | 280.1 (237.9 to 323.9) | -31.3 (-42.4 to -17.4) | 5120.1 (4340.2 to 5935.8) | -36.6 (-46.8 to -23.6) |
| Japan | 5040.4 (4708.9 to 5378.6) | -9.5 (-12.0 to -6.6) | 423.2 (395.9 to 454.3) | -21.4 (-23.6 to -19.3) | 72.5 (60.8 to 78.8) | -61.1 (-63.2 to -59.8) | 1651.0 (1498.4 to 1780.4) | -53.0 (-54.6 to -51.5) |
| Mongolia | 8167.1 (7698.1 to 8634.0) | 0.4 (-2.1 to 3.1) | 1185.2 (1060.3 to 1322.5) | -5.9 (-9.0 to -2.5) | 390.3 (346.6 to 430.3) | -33.4 (-40.8 to -24.8) | 7981.4 (7159.0 to 8816.0) | -34.8 (-42.5 to -25.6) |
| North Korea | 6428.0 (6079.5 to 6760.0) | 0.0 (-2.1 to 2.3) | 845.4 (781.6 to 914.7) | -0.3 (-3.9 to 4.0) | 358.9 (296.6 to 426.3) | -6.4 (-24.0 to 17.4) | 7813.0 (6375.8 to 9425.8) | -4.0 (-24.0 to 22.6) |
| South Korea | 4930.9 (4653.8 to 5181.4) | -21.8 (-24.0 to -19.2) | 423.5 (399.0 to 452.4) | -47.0 (-49.2 to -44.9) | 85.9 (70.7 to 95.9) | -74.0 (-76.9 to -71.6) | 1698.4 (1499.4 to 1884.4) | -73.7 (-75.8 to -71.3) |
| Taiwan [Province of China] | 6521.0 (6157.4 to 6851.2) | -3.6 (-6.3 to -1.1) | 676.1 (610.6 to 750.3) | -17.3 (-20.2 to -14.3) | 89.4 (78.7 to 95.8) | -64.5 (-67.2 to -62.5) | 2107.1 (1921.9 to 2258.0) | -59.4 (-61.9 to -57.3) |
| **Southeast Asia** | **5832.5 (5460.9 to 6152.7)** | **2.8 (1.7 to 3.9)** | **675.1 (627.4 to 730.3)** | **-3.5 (-4.7 to -2.2)** | **302.9 (273.2 to 326.1)** | **-14.8 (-23.9 to -3.4)** | **6714.6 (6071.1 to 7285.1)** | **-17.2 (-25.9 to -7.6)** |
| Brunei Darussalam | 4907.0 (4647.2 to 5161.0) | -19.8 (-12.0 to -6.6) | 500.4 (464.2 to 539.7) | -25.8 (-28.0 to -23.3) | 198.5 (176.9 to 222.7) | -44.1 (-50.8 to -35.6) | 4115.5 (3735.9 to 4557.4) | -45.8 (-52.4 to -36.9) |
| Cambodia | 5516.8 (5175.1 to 5800.8) | 3.8 (1.7 to 6.0) | 706.8 (659.2 to 760.2) | 0.3 (-2.7 to 3.9) | 341.8 (281.0 to 398.6) | -16.9 (-34.2 to 1.8) | 6971.0 (5737.5 to 8341.6) | -25.4 (-40.6 to -6.0) |
| Indonesia | 6076.5 (5642.8 to 6485.1) | 5.0 (3.4 to 6.9) | 764.0 (699.4 to 834.5) | 6.4 (4.8 to 8.0) | 409.9 (343.7 to 459.4) | 15.1 (-6.4 to 39.6) | 8680.4 (7378.5 to 9908.1) | 2.6 (-14.5 to 22.4) |
| Lao PDR | 5952.4 (5591.2 to 6299.9) | -0.7 (-2.8 to 1.4) | 730.7 (676.4 to 792.1) | -7.7 (-10.3 to -4.9) | 410.9 (337.2 to 485.9) | -32.7 (-47.5 to -13.1) | 8921.9 (7189.7 to 10805.3) | -39.1 (-53.2 to -19.2) |
| Malaysia | 7264.9 (6868.6 to 7648.2) | 6.6 (4.5 to 8.6) | 752.2 (699.3 to 812.3) | -6.7 (-10.0 to -3.4) | 267.9 (250.1 to 282.6) | -22.8 (-27.1 to -17.7) | 5946.1 (5641.6 to 6246.6) | -26.1 (-29.7 to -21.4) |
| Myanmar | 5873.4 (5512.2 to 6173.8) | -5.7 (-7.9 to -3.5) | 705.2 (661.8 to 757.4) | -14.6 (-17.4 to -12.0) | 363.6 (306.7 to 439.0) | -34.0 (-49.0 to -14.3) | 7873.6 (6574.2 to 9553.1) | -40.3 (-54.0 to -22.0) |
| Philippines | 5673.9 (5244.0 to 6038.7) | 3.4 (2.1 to 4.7) | 603.3 (555.6 to 656.4) | 0.1 (-3.0 to 2.8) | 315.1 (272.0 to 359.8) | -13.2 (-24.9 to 0.2) | 7252.9 (6231.6 to 8292.0) | -5.1 (-18.9 to 10.8) |
| Singapore | 4579.6 (4347.2 to 4791.4) | -19.8 (-22.0 to -17.7) | 428.6 (389.8 to 472.1) | -36.2 (-38.4 to -34.0) | 75.8 (67.4 to 80.9) | -72.4 (-74.4 to -71.0) | 1730.9 (1593.0 to 1839.6) | -71.9 (-73.3 to -70.6) |
| Thailand | 5634.5 (5303.0 to 5956.7) | -1.8 (-3.6 to 0.2) | 552.2 (516.1 to 595.4) | -16.9 (-18.9 to -14.8) | 128.0 (101.4 to 156.2) | -43.2 (-55.4 to -28.0) | 3126.6 (2552.9 to 3754.9) | -37.0 (-49.4 to -22.1) |
| Timor-Leste | 5597.0 (5270.0 to 5911.6) | 4.7 (2.6 to 6.9) | 663.3 (604.3 to 728.0) | 4.5 (1.4 to 7.1) | 380.4 (308.9 to 458.4) | 3.2 (-20.6 to 34.9) | 8053.2 (6513.5 to 9837.1) | -2.4 (-25.2 to 26.7) |
| Viet Nam | 5421.2 (5128.6 to 5694.6) | 9.2 (7.0 to 11.4) | 670.0 (629.8 to 715.0) | 2.2 (-1.0 to 5.9) | 310.5 (262.2 to 351.1) | -9.3 (-28.4 to 13.0) | 6181.9 (5223.1 to 7097.2) | -13.4 (-32.7 to 9.9) |
| **South Asia** | **7419.9 (6732.3 to 8143.3)** | **4.9 (2.5 to 8.0)** | **912.2 (805.6 to 1032.6)** | **-4.7 (-5.8 to -3.6)** | **278.1 (256.5 to 299.6)** | **-8.0 (-17.7 to 2.6)** | **6339.9 (5860.4 to 6836.7)** | **-14.1 (-23.1 to -4.9)** |
| Afghanistan | 9632.4 (9097.4 to 10204.1) | -2.0 (-5.0 to 1.2) | 1346.9 (1215.6 to 1512.5) | -13.9 (-18.1 to -8.7) | 567.4 (460.6 to 672.2) | -23.9 (-38.1 to -6.0) | 12478.3 (10077.8 to 15039.0) | -27.6 (-42.5 to -8.8) |
| Bangladesh | 7253.6 (6780.3 to 7681.2) | 3.7 (1.0 to 6.5) | 839.1 (770.1 to 917.3) | -7.0 (-11.2 to -2.8) | 313.0 (259.9 to 372.7) | -21.1 (-36.8 to -2.5) | 6573.3 (5427.4 to 7920.3) | -30.6 (-44.8 to -13.5) |
| Bhutan | 7351.8 (6854.5 to 7865.9) | 5.6 (2.0 to 9.4) | 893.0 (761.5 to 1048.3) | -2.3 (-5.8 to 1.0) | 233.1 (192.1 to 272.4) | -17.4 (-36.3 to 9.4) | 4950.5 (4007.9 to 5920.4) | -26.1 (-43.0 to -3.7) |
| India | 7339.4 (6607.5 to 8117.8) | 5.6 (2.9 to 9.0) | 905.2 (791.7 to 1031.3) | -5.1 (-6.2 to -4.1) | 267.6 (243.2 to 293.4) | -6.9 (-18.4 to 5.4) | 6151.1 (5606.0 to 6737.6) | -13.7 (-23.9 to -2.7) |
| Iran | 10385.9 (9450.6 to 11390.4) | 4.3 (1.3 to 8.0) | 1197.1 (1008.8 to 1398.4) | -10.5 (-12.1 to -8.8) | 254.5 (230.2 to 271.3) | -43.0 (-45.9 to -40.1) | 4977.4 (4594.5 to 5292.4) | -45.3 (-48.1 to -42.4) |
| Maldives | 6390.4 (5998.4 to 6767.8) | -4.5 (-6.8 to -2.1) | 611.1 (553.6 to 675.2) | -17.2 (-19.6 to -14.6) | 180.5 (150.5 to 208.3) | -58.4 (-64.8 to -51.5) | 3623.3 (3080.3 to 4153.9) | -64.6 (-70.5 to -58.4) |
| Nepal | 6514.6 (6108.0 to 6916.2) | -0.8 (-3.6 to 2.2) | 833.1 (746.7 to 931.6) | -5.2 (-9.4 to -0.7) | 278.3 (234.6 to 336.6) | -14.5 (-32.9 to 9.9) | 5973.3 (4959.3 to 7300.9) | -22.7 (-39.4 to -1.4) |
| Pakistan | 8450.9 (7665.8 to 9305.7) | 3.4 (0.6 to 6.6) | 1066.2 (931.9 to 1212.4) | 2.6 (0.4 to 4.9) | 342.0 (292.3 to 416.6) | 10.0 (-8.2 to 35.8) | 7782.4 (6619.3 to 9417.9) | 7.4 (-10.7 to 32.6) |
| Sri Lanka | 5476.4 (5157.2 to 5764.6) | 2.6 (0.4 to 4.7) | 614.5 (570.2 to 665.7) | -7.1 (-9.9 to -4.2) | 232.3 (167.8 to 299.8) | -37.3 (-55.4 to -18.6) | 4596.8 (3303.8 to 5947.9) | -38.0 (-55.7 to -19.7) |
| Armenia | 8266.1 (7722.9 to 8725.2) | 6.6 (3.7 to 9.3) | 976.0 (869.4 to 1092.1) | -10.7 (-14.0 to -7.5) | 314.8 (281.0 to 348.5) | -32.9 (-38.5 to -26.7) | 6038.4 (5491.9 to 6673.5) | -33.8 (-39.5 to -27.3) |
| Azerbaijan | 8015.1 (7542.8 to 8437.7) | 4.6 (2.2 to 7.1) | 1267.9 (1179.0 to 1367.6) | 16.5 (10.6 to 22.3) | 449.6 (401.1 to 498.4) | -16.7 (-24.6 to -7.5) | 8624.7 (7704.9 to 9576.3) | -23.1 (-31.1 to -13.9) |
| Bahrain | 9655.2 (9031.9 to 10304.3) | 2.0 (-1.5 to 6.0) | 1284.5 (1057.5 to 1553.0) | -6.7 (-11.0 to -3.1) | 289.9 (257.3 to 325.0) | -54.5 (-60.0 to -48.3) | 5054.7 (4447.4 to 5647.6) | -57.8 (-63.0 to -51.9) |
| Cyprus | 5040.4 (4645.9 to 5396.3) | -12.0 (-14.3 to -9.4) | 486.9 (450.9 to 526.6) | -22.2 (-24.6 to -19.8) | 184.9 (162.5 to 205.1) | -66.7 (-70.2 to -63.0) | 2850.3 (2549.5 to 3154.0) | -65.4 (-69.2 to -61.3) |
| Georgia | 8820.1 (8307.3 to 9307.6) | 4.1 (1.4 to 6.8) | 1097.4 (995.2 to 1205.6) | -15.7 (-19.8 to -10.7) | 355.2 (319.9 to 387.9) | -39.1 (-44.1 to -33.9) | 7202.4 (6589.6 to 7815.5) | -39.1 (-44.0 to -34.2) |
| Iraq | 10418.9 (9875.4 to 11011.5) | 2.6 (0.0 to 6.0) | 1455.3 (1320.0 to 1620.4) | 1.1 (-3.9 to 6.6) | 477.6 (397.1 to 553.4) | -6.0 (-25.8 to 13.7) | 9361.9 (7578.8 to 10934.8) | -19.3 (-37.7 to 0.6) |
| Israel | 5946.4 (5548.4 to 6310.8) | -3.5 (-6.9 to 0.3) | 540.0 (498.9 to 586.4) | -27.1 (-29.9 to -24.1) | 75.2 (64.4 to 81.2) | -72.2 (-74.3 to -70.6) | 1500.9 (1346.2 to 1631.1) | -70.2 (-72.2 to -68.4) |
| Jordan | 9490.5 (8942.3 to 10067.7) | 1.8 (-1.5 to 4.9) | 1249.5 (1067.8 to 1467.6) | -7.2 (-11.5 to -3.2) | 213.4 (177.5 to 252.6) | -49.2 (-58.7 to -36.5) | 4232.1 (3565.3 to 5055.7) | -52.1 (-61.2 to -39.9) |
| Kuwait | 9993.1 (9373.3 to 10671.7) | 1.9 (-1.3 to 5.2) | 1336.0 (1108.7 to 1604.3) | -1.8 (-6.6 to 3.4) | 167.8 (138.0 to 200.2) | -44.5 (-53.3 to -34.2) | 3568.0 (3008.2 to 4249.6) | -46.4 (-54.7 to -36.4) |
| Lebanon | 10311.1 (9711.4 to 10933.8) | 6.7 (3.3 to 9.9) | 1238.3 (1059.5 to 1428.4) | -5.7 (-10.1 to -1.2) | 164.5 (138.9 to 191.8) | -62.8 (-69.3 to -55.2) | 3323.3 (2898.3 to 3821.1) | -64.4 (-71.0 to -57.0) |
| Oman | 10991.9 (10301.6 to 11746.5) | -0.7 (-4.6 to 3.2) | 1333.2 (1133.8 to 1564.9) | 0.9 (-4.0 to 5.8) | 326.4 (272.3 to 379.8) | -37.7 (-52.2 to -17.6) | 6441.6 (5427.1 to 7551.7) | -45.0 (-58.0 to -27.1) |
| Palestine | 9281.2 (8707.0 to 9865.1) | 2.1 (-1.4 to 5.4) | 1309.2 (1127.2 to 1524.8) | -1.7 (-5.4 to 1.9) | 356.8 (311.7 to 397.1) | -37.2 (-48.9 to -24.0) | 6469.1 (5680.1 to 7184.5) | -40.1 (-51.4 to -26.2) |
| Qatar | 10014.5 (9425.3 to 10708.2) | -1.8 (-5.1 to 1.5) | 1312.8 (1075.8 to 1587.5) | -5.9 (-9.4 to -2.3) | 207.0 (166.7 to 247.5) | -66.1 (-73.0 to -58.2) | 3768.2 (3061.1 to 4489.7) | -66.5 (-73.6 to -58.3) |
| Saudi Arabia | 9918.3 (9320.0 to 10521.4) | 14.9 (11.3 to 18.5) | 1218.1 (1076.5 to 1379.6) | 2.9 (-2.3 to 8.5) | 353.3 (305.3 to 408.4) | -25.1 (-40.5 to -0.3) | 7663.8 (6525.2 to 8994.0) | -23.7 (-40.5 to 2.2) |
| Syrian Arab Republic | 10460.1 (9903.1 to 11036.9) | 0.0 (-3.0 to 2.8) | 1480.4 (1334.6 to 1657.0) | 0.9 (-4.2 to 6.0) | 521.6 (422.6 to 639.1) | -13.4 (-34.1 to 14.3) | 10120.0 (8054.9 to 12795.0) | -24.2 (-43.3 to 1.9) |
| Turkey | 8562.8 (7985.4 to 9114.2) | -8.0 (-11.3 to -4.7) | 939.2 (837.9 to 1058.4) | -19.3 (-24.6 to -13.6) | 247.9 (206.2 to 288.5) | -40.1 (-49.9 to -30.3) | 4615.0 (3896.9 to 5379.7) | -47.5 (-56.4 to -38.4) |
| United Arab Emirates | 11066.8 (10490.2 to 11668.2) | 2.1 (-0.4 to 4.8) | 1464.5 (1235.1 to 1723.2) | -3.4 (-7.5 to 1.6) | 314.1 (261.1 to 366.1) | -33.6 (-42.0 to -24.6) | 5758.5 (4881.6 to 6694.1) | -43.2 (-50.6 to -34.9) |
| Yemen | 9722.2 (9129.2 to 10312.6) | 3.1 (-0.0 to 6.0) | 1318.7 (1187.1 to 1480.5) | -5.6 (-10.6 to -0.1) | 517.8 (415.8 to 637.9) | -19.9 (-37.3 to 3.6) | 10703.2 (8442.4 to 13415.0) | -25.8 (-43.0 to -1.9) |

ASR: Age-standardized rate; UI: Uncertainty interval; DALYs: Disability-adjusted life years

| **Table S5. Ischemic heart disease burden by country.** | | | | | | | | |
| --- | --- | --- | --- | --- | --- | --- | --- | --- |
|  | **Prevalence (95%UI)** | | **Incidence (95%UI)** | | **Death (95%UI)** | | **DALYs (95%UI)** | |
| **Location** | **ASR (per 100,000) 2021** | **Percent change, 1990-2021** | **ASR (per 100,000) 2021** | **Percent change, 1990-2021** | **ASR (per 100,000) 2021** | **Percent change, 1990-2021** | **ASR (per 100,000) 2021** | **Percent change, 1990-2021** |
| **Central Asia** | **4408.4 (4040.6 to 4801.1)** | **7.6 (4.2 to 11.3)** | **801.6 (732.0 to 893.8)** | **24.9 (19.5 to 31.3)** | **265.5 (240.7 to 290.4)** | **-17.1 (-24.5 to -10.2)** | **4864.5 (4415.5 to 5338.7)** | **-21.6 (-29.1 to -14.3)** |
| Kazakhstan | 4230.9 (3828.9 to 4677.0) | -2.3 (-7.1 to 2.9) | 584.4 (515.4 to 678.0) | -7.5 (-12.6 to -2.2) | 236.0 (212.0 to 258.9) | -21.0 (-28.9 to -12.5) | 3970.5 (3569.8 to 4401.1) | -31.9 (-39.3 to -23.3) |
| Kyrgyzstan | 3492.3 (3183.7 to 3858.4) | -5.8 (-10.5 to -1.2) | 594.1 (510.7 to 697.8) | 3.4 (-3.7 to 10.8) | 274.4 (234.2 to 313.7) | 3.8 (-11.1 to 18.7) | 4780.5 (4093.6 to 5490.6) | -7.2 (-22.1 to 8.0) |
| Tajikistan | 4105.8 (3730.2 to 4525.3) | 6.2 (1.7 to 10.7) | 774.6 (678.9 to 883.8) | 31.8 (20.2 to 44.8) | 244.9 (206.6 to 281.6) | -19.7 (-32.5 to -4.5) | 4400.4 (3740.1 to 5106.8) | -26.2 (-38.0 to -12.4) |
| Turkmenistan | 4460.7 (4029.4 to 4943.3) | 7.3 (1.9 to 12.8) | 689.7 (588.8 to 796.6) | -3.5 (-8.9 to 2.2) | 343.7 (280.9 to 420.0) | -15.6 (-31.0 to 3.7) | 6512.7 (5219.5 to 8027.7) | -17.2 (-33.2 to 3.2) |
| Uzbekistan | 5015.6 (4660.9 to 5391.6) | 30.4 (25.7 to 35.1) | 1206.0 (1125.0 to 1305.5) | 107.2 (88.8 to 129.0) | 339.5 (295.6 to 382.0) | 6.5 (-6.9 to 19.9) | 6218.8 (5449.8 to 7058.1) | 1.6 (-11.5 to 15.8) |
| **East Asia** | **3031.2 (2597.7 to 3606.2)** | **19.6 (13.7 to 26.7)** | **363.6 (291.9 to 437.5)** | **15.0 (12.6 to 17.3)** | **108.9 (91.2 to 125.8)** | **15.9 (-2.6 to 38.1)** | **1839.9 (1541.2 to 2135.3)** | **3.9 (-13.9 to 26.4)** |
| China | 3042.4 (2601.7 to 3629.9) | 20.4 (14.5 to 27.7) | 365.7 (293.3 to 440.1) | 16.0 (13.5 to 18.4) | 110.9 (92.4 to 128.6) | 17.8 (-1.6 to 41.1) | 1856.5 (1548.7 to 2159.8) | 4.8 (-13.6 to 28.1) |
| Japan | 802.3 (686.8 to 938.2) | -15.0 (-20.1 to -10.2) | 90.6 (71.5 to 114.0) | -1.3 (-4.9 to 2.3) | 25.4 (21.7 to 27.3) | -62.0 (-63.5 to -60.9) | 502.3 (460.0 to 526.3) | -56.0 (-56.8 to -55.2) |
| Mongolia | 4213.8 (3806.6 to 4658.6) | -2.9 (-7.8 to 2.9) | 669.1 (547.5 to 799.8) | -11.3 (-15.8 to -6.0) | 219.6 (190.3 to 247.4) | -32.4 (-42.7 to -20.8) | 3892.3 (3366.1 to 4387.1) | -35.8 (-46.1 to -23.8) |
| North Korea | 2631.7 (2382.0 to 2915.8) | 3.3 (-1.9 to 8.2) | 346.2 (293.7 to 411.1) | 4.0 (-5.3 to 13.7) | 125.3 (100.8 to 153.3) | 11.3 (-12.1 to 42.4) | 2594.2 (2058.2 to 3211.8) | 15.1 (-12.7 to 52.1) |
| South Korea | 841.8 (748.1 to 948.8) | -24.7 (-29.4 to -19.4) | 96.3 (79.3 to 116.3) | -53.6 (-58.3 to -48.1) | 28.3 (22.9 to 32.6) | -53.0 (-59.1 to -45.8) | 471.0 (402.6 to 526.8) | -56.6 (-61.2 to -50.8) |
| Taiwan [Province of China] | 2817.3 (2556.5 to 3144.3) | -2.6 (-7.7 to 3.0) | 303.2 (243.9 to 375.5) | -14.2 (-19.8 to -8.4) | 33.6 (30.0 to 36.2) | -54.4 (-57.7 to -51.6) | 684.7 (626.6 to 728.3) | -49.1 (-52.7 to -45.9) |
| **Southeast Asia** | 2088.4 (1847.4 to 2376.7) | 4.3 (0.7 to 7.7) | 231.6 (193.1 to 275.9) | -3.5 (-6.0 to -1.0) | 110.9 (100.2 to 120.2) | -3.3 (-14.7 to 9.9) | 2415.6 (2177.9 to 2635.3) | -5.2 (-16.5 to 7.0) |
| Brunei Darussalam | 937.7 (832.6 to 1056.2) | -16.2 (-20.9 to -10.8) | 115.5 (90.1 to 146.8) | -11.3 (-15.9 to -6.1) | 84.5 (74.3 to 98.9) | -43.5 (-52.0 to -33.7) | 1726.3 (1525.6 to 1929.9) | -44.7 (-53.3 to -34.3) |
| Cambodia | 1828.6 (1645.6 to 2037.8) | 7.0 (2.2 to 11.9) | 237.8 (197.7 to 281.6) | 6.4 (-1.5 to 16.3) | 111.5 (89.2 to 134.6) | -3.6 (-26.3 to 20.6) | 2251.2 (1767.8 to 2790.1) | -13.7 (-34.8 to 11.1) |
| Indonesia | 2349.9 (1998.8 to 2782.4) | 12.7 (7.7 to 18.5) | 270.6 (215.3 to 331.5) | 12.7 (10.3 to 15.4) | 143.3 (119.3 to 163.3) | 39.1 (11.2 to 74.2) | 3043.1 (2527.3 to 3544.7) | 25.7 (0.8 to 54.8) |
| Lao PDR | 1968.3 (1773.7 to 2200.5) | 0.3 (-4.9 to 5.2) | 250.2 (205.2 to 302.2) | -4.2 (-10.7 to 2.3) | 176.5 (141.7 to 213.3) | -25.0 (-43.1 to 0.1) | 3667.1 (2896.8 to 4573.2) | -32.9 (-50.2 to -7.4) |
| Malaysia | 2857.8 (2581.7 to 3143.7) | 10.6 (5.5 to 15.8) | 313.1 (271.0 to 364.9) | 0.7 (-6.4 to 10.0) | 149.7 (138.2 to 159.7) | -15.7 (-22.9 to -7.9) | 3180.4 (2984.1 to 3352.2) | -18.0 (-25.0 to -10.7) |
| Myanmar | 1927.9 (1745.9 to 2132.7) | -7.0 (-11.6 to -2.1) | 241.7 (208.6 to 279.9) | -14.4 (-19.4 to -8.7) | 138.2 (113.0 to 171.6) | -28.5 (-45.6 to -3.9) | 2773.1 (2241.1 to 3446.1) | -36.5 (-52.8 to -12.7) |
| Philippines | 1881.9 (1604.1 to 2205.6) | -5.2 (-8.4 to -1.3) | 177.2 (140.1 to 219.9) | -19.0 (-22.6 to -15.7) | 150.4 (129.2 to 171.9) | -13.6 (-26.0 to 0.3) | 3326.5 (2831.6 to 3850.5) | -5.5 (-19.8 to 12.4) |
| Singapore | 1350.8 (1280.8 to 1431.8) | -7.0 (-13.1 to -0.2) | 149.9 (117.6 to 188.0) | -31.5 (-35.3 to -27.0) | 46.7 (41.8 to 49.9) | -68.5 (-70.7 to -66.8) | 934.1 (866.5 to 985.5) | -70.6 (-72.1 to -69.1) |
| Thailand | 1732.2 (1551.2 to 1933.3) | -5.3 (-10.5 to 0.0) | 169.6 (140.0 to 200.9) | -22.5 (-26.6 to -18.2) | 47.1 (36.8 to 58.6) | -44.3 (-57.4 to -27.6) | 1039.7 (825.6 to 1278.9) | -38.3 (-52.3 to -21.0) |
| Timor-Leste | 1887.3 (1692.8 to 2134.9) | 7.9 (3.1 to 14.1) | 221.8 (174.7 to 277.0) | 5.0 (-1.9 to 12.2) | 156.9 (121.1 to 196.0) | 27.8 (-6.5 to 69.7) | 3163.0 (2433.5 to 4007.9) | 21.2 (-12.6 to 64.2) |
| Viet Nam | 1961.3 (1779.9 to 2182.1) | 11.5 (6.6 to 17.4) | 232.7 (202.0 to 270.5) | 6.1 (-1.1 to 15.3) | 77.0 (63.5 to 90.7) | 16.0 (-10.8 to 48.0) | 1464.6 (1198.7 to 1740.5) | 10.3 (-16.3 to 44.0) |
| **South Asia** | 4455.7 (3796.7 to 5339.2) | 6.8 (2.2 to 12.2) | 580.2 (472.9 to 704.6) | -3.3 (-4.8 to -1.6) | 149.1 (137.0 to 161.2) | 9.3 (-2.2 to 23.3) | 3351.1 (3075.4 to 3616.4) | 2.3 (-9.0 to 15.1) |
| Afghanistan | 6317.6 (5785.3 to 6886.8) | -7.5 (-12.7 to -2.5) | 964.6 (833.2 to 1126.0) | -16.5 (-21.9 to -10.3) | 280.0 (221.4 to 346.2) | -22.5 (-39.2 to -2.2) | 6178.3 (4747.6 to 7813.3) | -26.2 (-43.6 to -4.1) |
| Bangladesh | 3969.6 (3596.9 to 4375.7) | 3.1 (-2.0 to 8.2) | 469.8 (405.9 to 551.3) | -8.1 (-15.0 to -1.3) | 107.5 (86.2 to 131.7) | -9.8 (-29.9 to 14.8) | 2366.9 (1856.4 to 2929.7) | -21.2 (-40.0 to 1.7) |
| Bhutan | 4367.5 (3913.4 to 4869.9) | 6.5 (-0.3 to 13.4) | 587.3 (453.8 to 739.4) | 0.2 (-4.8 to 5.0) | 111.0 (86.5 to 133.9) | 4.0 (-20.6 to 40.2) | 2299.5 (1758.0 to 2871.3) | -8.0 (-31.5 to 26.1) |
| India | 4458.2 (3744.5 to 5412.8) | 8.4 (3.4 to 14.5) | 585.2 (470.6 to 713.2) | -3.7 (-5.2 to -2.2) | 151.2 (137.5 to 165.1) | 9.6 (-3.6 to 26.3) | 3400.0 (3098.5 to 3720.8) | 2.4 (-10.3 to 17.5) |
| Iran | 6327.2 (5393.2 to 7502.0) | -2.6 (-7.1 to 2.4) | 887.1 (698.9 to 1089.0) | -11.2 (-13.4 to -9.1) | 146.1 (130.7 to 157.0) | -43.8 (-47.1 to -40.1) | 2731.3 (2517.3 to 2920.9) | -47.1 (-50.1 to -43.6) |
| Maldives | 2050.9 (1821.8 to 2311.7) | -5.6 (-10.2 to -0.3) | 221.4 (173.7 to 279.0) | -7.3 (-11.6 to -1.5) | 79.4 (66.2 to 93.8) | -52.7 (-61.1 to -42.9) | 1481.0 (1238.4 to 1760.0) | -61.2 (-69.0 to -53.0) |
| Nepal | 3740.0 (3407.5 to 4131.3) | -2.4 (-7.3 to 2.6) | 511.9 (431.3 to 615.4) | -5.1 (-11.9 to 2.5) | 136.5 (111.7 to 169.9) | 4.9 (-19.0 to 39.7) | 2890.1 (2320.6 to 3596.1) | -4.9 (-27.1 to 25.3) |
| Pakistan | 5155.3 (4349.3 to 6133.6) | 3.9 (-1.9 to 10.4) | 673.6 (540.9 to 825.3) | 6.1 (2.7 to 9.8) | 183.4 (154.6 to 226.8) | 30.4 (6.8 to 66.3) | 4069.0 (3417.0 to 4985.8) | 28.2 (4.1 to 66.0) |
| Sri Lanka | 2204.1 (1990.8 to 2458.3) | -2.2 (-6.7 to 2.2) | 248.5 (210.6 to 290.2) | -10.7 (-15.9 to -5.5) | 94.6 (64.8 to 125.4) | -31.7 (-52.9 to -7.6) | 1963.6 (1330.0 to 2662.4) | -31.5 (-53.7 to -5.9) |
| Armenia | 4081.4 (3674.1 to 4505.4) | 6.0 (0.6 to 11.5) | 591.0 (490.4 to 710.6) | -9.0 (-14.4 to -3.8) | 209.8 (186.4 to 233.4) | -31.8 (-38.2 to -24.4) | 3788.5 (3403.8 to 4233.6) | -33.2 (-39.9 to -25.2) |
| Azerbaijan | 4415.2 (4047.7 to 4805.9) | 8.2 (3.1 to 13.0) | 806.3 (725.6 to 906.9) | 23.2 (13.1 to 32.8) | 306.1 (270.8 to 343.5) | -15.5 (-25.4 to -5.0) | 5496.3 (4818.7 to 6182.3) | -23.2 (-33.0 to -13.0) |
| Bahrain | 7066.0 (6420.8 to 7782.3) | -2.0 (-7.9 to 4.2) | 1023.5 (792.3 to 1294.1) | -6.1 (-11.6 to -1.5) | 161.6 (140.6 to 184.4) | -60.4 (-65.2 to -54.8) | 2796.6 (2418.6 to 3179.5) | -63.6 (-68.7 to -58.2) |
| Cyprus | 1198.4 (1033.8 to 1385.6) | -9.1 (-14.0 to -3.5) | 119.6 (95.4 to 148.3) | -21.1 (-27.1 to -15.1) | 87.0 (75.8 to 98.1) | -68.0 (-72.8 to -62.6) | 1395.0 (1225.3 to 1564.9) | -66.2 (-71.1 to -60.8) |
| Georgia | 4117.7 (3704.6 to 4551.6) | -6.7 (-11.9 to -1.4) | 553.8 (458.9 to 662.1) | -29.0 (-36.2 to -21.5) | 124.2 (111.0 to 136.5) | -65.4 (-68.7 to -61.9) | 2565.2 (2301.9 to 2822.2) | -62.7 (-66.4 to -58.9) |
| Iraq | 7185.5 (6654.6 to 7885.0) | 4.1 (-0.5 to 10.2) | 1040.6 (915.1 to 1203.0) | 1.1 (-5.9 to 9.1) | 254.8 (204.2 to 296.7) | 2.1 (-20.7 to 26.6) | 4905.3 (3858.3 to 5827.7) | -11.2 (-33.1 to 13.3) |
| Israel | 1435.4 (1255.6 to 1633.6) | -23.3 (-27.7 to -18.5) | 153.2 (122.6 to 189.0) | -44.4 (-49.3 to -39.6) | 34.8 (29.9 to 37.5) | -80.4 (-82.0 to -79.3) | 599.6 (537.8 to 637.8) | -81.2 (-82.4 to -80.2) |
| Jordan | 6817.0 (6205.0 to 7546.4) | 2.3 (-3.0 to 7.3) | 913.4 (737.8 to 1134.2) | -6.1 (-12.0 to -0.5) | 98.3 (80.7 to 117.7) | -50.7 (-60.5 to -38.8) | 1947.2 (1603.4 to 2349.6) | -53.9 (-63.6 to -42.3) |
| Kuwait | 7806.4 (7141.6 to 8556.3) | 0.9 (-3.9 to 5.7) | 1061.2 (836.1 to 1330.5) | -2.9 (-8.8 to 3.6) | 109.1 (90.4 to 131.4) | -45.0 (-54.3 to -34.7) | 2299.4 (1917.0 to 2762.5) | -46.5 (-56.0 to -35.8) |
| Lebanon | 6814.6 (6242.7 to 7463.0) | 7.5 (1.7 to 12.5) | 931.4 (756.1 to 1127.8) | -7.1 (-12.6 to -1.1) | 92.1 (77.7 to 108.0) | -60.9 (-68.7 to -51.3) | 1735.3 (1488.4 to 2027.4) | -64.7 (-72.0 to -55.5) |
| Oman | 6854.9 (6259.0 to 7535.7) | 8.6 (2.2 to 14.7) | 1027.1 (829.7 to 1253.8) | 0.8 (-5.7 to 6.9) | 179.3 (150.3 to 211.7) | -40.5 (-54.7 to -21.0) | 3393.7 (2837.0 to 4065.1) | -47.7 (-61.2 to -29.2) |
| Palestine | 6302.6 (5748.3 to 6947.2) | 2.4 (-3.1 to 7.9) | 942.1 (762.4 to 1163.1) | -1.7 (-6.3 to 3.5) | 188.7 (162.7 to 211.7) | -34.7 (-46.8 to -19.9) | 3458.7 (3014.4 to 3871.8) | -37.5 (-50.6 to -20.8) |
| Qatar | 7143.8 (6501.0 to 7864.4) | -3.2 (-8.4 to 1.9) | 1032.1 (800.6 to 1299.5) | -3.2 (-8.1 to 1.5) | 123.2 (97.9 to 147.9) | -68.0 (-75.1 to -60.3) | 2139.5 (1690.4 to 2627.2) | -69.6 (-76.7 to -61.4) |
| Saudi Arabia | 7341.6 (6747.5 to 7966.8) | 17.1 (11.4 to 23.0) | 920.1 (778.0 to 1085.3) | 4.8 (-1.6 to 12.6) | 185.9 (158.1 to 219.1) | -17.6 (-36.3 to 14.0) | 4219.8 (3530.6 to 5082.9) | -13.7 (-35.1 to 22.5) |
| Syrian Arab Republic | 6859.5 (6321.6 to 7467.6) | 2.3 (-2.6 to 7.1) | 1130.3 (993.5 to 1305.6) | 4.1 (-2.6 to 10.7) | 353.0 (281.6 to 432.0) | -4.8 (-27.6 to 26.5) | 6688.8 (5230.4 to 8518.9) | -15.2 (-38.2 to 17.4) |
| Turkey | 5365.9 (4895.5 to 5874.7) | -11.9 (-17.3 to -6.9) | 665.1 (568.3 to 784.6) | -19.3 (-27.0 to -11.4) | 133.4 (109.9 to 155.9) | -34.8 (-46.4 to -21.8) | 2419.3 (2008.1 to 2857.0) | -43.1 (-53.7 to -31.4) |
| United Arab Emirates | 7608.9 (6995.2 to 8286.0) | 4.6 (0.1 to 9.4) | 1084.3 (843.4 to 1343.9) | -0.1 (-5.9 to 7.8) | 167.7 (136.6 to 196.3) | -37.2 (-46.0 to -26.2) | 2923.7 (2395.6 to 3422.0) | -47.6 (-55.7 to -37.3) |
| Yemen | 6182.8 (5696.1 to 6755.9) | -1.4 (-6.1 to 3.2) | 948.5 (819.7 to 1114.4) | -6.7 (-13.1 to 0.6) | 263.5 (201.8 to 344.2) | -17.7 (-36.7 to 7.7) | 5442.6 (4100.2 to 7104.9) | -23.6 (-42.6 to 3.1) |

ASR: Age-standardized rate; UI: Uncertainty interval; DALYs: Disability-adjusted life years

| **Table S6. Stroke burden by country.** | | | | | | | | |
| --- | --- | --- | --- | --- | --- | --- | --- | --- |
|  | **Prevalence (95%UI)** | | **Incidence (95%UI)** | | **Death (95%UI)** | | **DALYs (95%UI)** | |
| **Location** | **ASR (per 100,000) 2021** | **Percent change, 1990-2021** | **ASR (per 100,000) 2021** | **Percent change, 1990-2021** | **ASR (per 100,000) 2021** | **Percent change, 1990-2021** | **ASR (per 100,000) 2021** | **Percent change, 1990-2021** |
| **Central Asia** | **1281.9 (1235.1 to 1330.7)** | **-11.7 (-13.1 to -10.0)** | **216.6 (200.7 to 232.7)** | **-8.8 (-12.1 to -5.4)** | **124.1 (112.5 to 135.5)** | **-22.2 (-28.2 to -15.3)** | **2565.9 (2336.7 to 2798.3)** | **-26.8 (-32.5 to -20.1)** |
| Kazakhstan | 1482.6 (1423.3 to 1546.1) | -20.1 (-22.9 to -17.2) | 227.3 (208.1 to 246.7) | -18.4 (-23.9 to -12.9) | 163.0 (144.2 to 183.1) | -10.0 (-21.2 to 3.0) | 3099.2 (2711.1 to 3486.1) | -19.3 (-29.4 to -7.5) |
| Kyrgyzstan | 1029.5 (985.3 to 1072.8) | -31.8 (-34.1 to -29.5) | 159.7 (145.1 to 175.8) | -38.2 (-42.1 to -33.6) | 93.1 (79.4 to 107.4) | -53.3 (-60.5 to -45.7) | 2190.0 (1870.2 to 2526.3) | -49.2 (-57.4 to -40.9) |
| Tajikistan | 1046.4 (996.3 to 1096.1) | -8.3 (-11.0 to -5.4) | 223.7 (205.3 to 244.1) | 11.9 (6.0 to 18.6) | 130.1 (105.4 to 154.8) | -19.0 (-36.2 to 1.9) | 2614.5 (2123.2 to 3122.5) | -25.8 (-41.2 to -7.3) |
| Turkmenistan | 1571.1 (1502.1 to 1638.5) | 18.5 (14.9 to 22.7) | 225.3 (206.4 to 245.1) | 5.9 (-0.5 to 11.6) | 160.5 (128.7 to 194.0) | 21.6 (-2.7 to 50.7) | 3848.7 (3090.4 to 4661.2) | 21.4 (-3.4 to 49.9) |
| Uzbekistan | 1349.9 (1294.6 to 1409.4) | -3.2 (-6.7 to 1.0) | 249.0 (229.6 to 270.3) | 9.9 (3.9 to 16.5) | 105.4 (92.2 to 121.5) | -20.8 (-31.3 to -7.8) | 2302.2 (2012.6 to 2639.8) | -28.0 (-37.7 to -16.4) |
| **East Asia** | **1302.7 (1204.6 to 1404.1)** | **10.0 (7.0 to 12.8)** | **203.8 (180.5 to 230.3)** | **-10.5 (-15.7 to -5.2)** | **136.2 (115.0 to 157.6)** | **-43.0 (-52.6 to -31.2)** | **2635.4 (2251.8 to 3049.7)** | **-45.0 (-54.1 to -33.8)** |
| China | 1301.4 (1200.6 to 1405.7) | 11.5 (8.3 to 14.5) | 204.8 (181.0 to 231.5) | -9.8 (-15.2 to -4.2) | 138.0 (116.7 to 160.3) | -43.0 (-52.8 to -30.9) | 2648.0 (2253.4 to 3076.9) | -45.2 (-54.4 to -33.7) |
| Japan | 1176.0 (1104.8 to 1247.5) | -14.1 (-16.8 to -10.5) | 97.0 (88.2 to 106.7) | -37.7 (-41.7 to -32.8) | 28.4 (23.8 to 31.0) | -67.1 (-69.0 to -65.6) | 710.5 (638.2 to 775.4) | -57.6 (-59.6 to -55.8) |
| Mongolia | 1309.0 (1261.9 to 1355.9) | -0.8 (-3.3 to 1.8) | 240.0 (225.1 to 255.0) | 0.5 (-4.1 to 5.4) | 134.1 (109.1 to 159.6) | -37.6 (-51.4 to -22.5) | 3036.3 (2536.0 to 3573.5) | -37.4 (-50.4 to -22.3) |
| North Korea | 1458.1 (1401.9 to 1523.2) | -6.6 (-9.4 to -3.5) | 248.6 (228.2 to 269.6) | -8.2 (-12.8 to -2.7) | 190.7 (154.5 to 232.4) | -12.8 (-30.9 to 12.2) | 4320.2 (3509.0 to 5234.6) | -9.4 (-29.7 to 16.9) |
| South Korea | 1240.9 (1203.5 to 1281.2) | -51.4 (-52.6 to -50.0) | 109.7 (99.0 to 120.8) | -67.1 (-69.0 to -65.2) | 41.2 (33.8 to 46.8) | -82.5 (-84.7 to -79.2) | 859.6 (754.7 to 961.1) | -81.5 (-83.2 to -78.9) |
| Taiwan [Province of China] | 1190.1 (1153.8 to 1229.1) | -26.1 (-28.2 to -24.2) | 126.7 (114.2 to 138.9) | -44.6 (-48.6 to -40.8) | 32.2 (28.7 to 34.8) | -76.4 (-78.3 to -74.8) | 865.8 (775.1 to 941.2) | -70.8 (-73.0 to -68.8) |
| **Southeast Asia** | **1292.1 (1223.2 to 1365.5)** | **-6.7 (-8.1 to -5.3)** | **188.0 (173.1 to 204.5)** | **-12.4 (-14.5 to -10.2)** | **154.8 (139.4 to 168.8)** | **-20.6 (-29.9 to -9.5)** | **3425.3 (3081.4 to 3736.6)** | **-22.8 (-31.3 to -13.1)** |
| Brunei Darussalam | 1384.9 (1340.6 to 1433.9) | -39.6 (-41.1 to -37.9) | 144.7 (131.3 to 158.0) | -45.4 (-48.5 to -41.9) | 71.1 (60.5 to 81.7) | -51.1 (-58.5 to -40.6) | 1509.0 (1324.4 to 1727.1) | -53.0 (-60.1 to -43.3) |
| Cambodia | 1063.0 (1023.1 to 1108.7) | -6.5 (-9.1 to -3.4) | 204.2 (189.3 to 221.7) | -11.1 (-15.5 to -6.6) | 185.2 (148.0 to 220.1) | -22.1 (-39.4 to -3.3) | 3709.4 (2911.2 to 4460.1) | -29.8 (-45.5 to -11.9) |
| Indonesia | 1526.6 (1405.3 to 1662.7) | -6.7 (-9.0 to -4.3) | 243.3 (218.6 to 270.9) | -0.1 (-3.7 to 3.9) | 217.7 (182.0 to 245.4) | 5.5 (-14.2 to 26.3) | 4587.1 (3889.4 to 5248.2) | -5.8 (-20.9 to 12.4) |
| Lao PDR | 1232.5 (1184.7 to 1283.3) | -10.8 (-13.5 to -8.2) | 204.9 (187.4 to 222.4) | -19.7 (-23.7 to -15.1) | 183.7 (148.5 to 223.8) | -38.2 (-52.9 to -19.9) | 4061.5 (3276.8 to 4988.7) | -43.3 (-57.6 to -26.5) |
| Malaysia | 1356.7 (1312.2 to 1405.2) | -9.1 (-11.4 to -6.6) | 163.2 (148.4 to 179.7) | -29.5 (-33.0 to -25.1) | 94.3 (84.5 to 104.8) | -32.7 (-40.1 to -23.6) | 2122.3 (1934.6 to 2323.9) | -36.1 (-42.2 to -28.7) |
| Myanmar | 1159.7 (1119.6 to 1203.4) | -15.4 (-17.6 to -13.0) | 187.8 (174.3 to 202.4) | -25.3 (-29.1 to -20.6) | 181.1 (147.6 to 222.2) | -37.5 (-52.5 to -16.7) | 4036.4 (3276.2 to 4964.3) | -42.6 (-56.5 to -23.5) |
| Philippines | 1158.5 (1074.2 to 1257.4) | 19.6 (16.7 to 22.3) | 159.1 (143.8 to 176.0) | 27.9 (22.7 to 33.7) | 114.8 (98.7 to 131.2) | -14.1 (-25.9 to -0.9) | 2761.8 (2384.5 to 3156.6) | -4.2 (-17.1 to 11.3) |
| Singapore | 1000.4 (966.6 to 1035.1) | -48.6 (-50.1 to -46.8) | 78.8 (70.4 to 88.8) | -63.1 (-66.0 to -60.2) | 14.2 (12.3 to 15.6) | -84.3 (-85.8 to -65.6) | 428.6 (377.9 to 476.4) | -79.6 (-81.3 to -78.0) |
| Thailand | 1050.2 (1018.7 to 1087.0) | -18.2 (-20.3 to -15.9) | 114.9 (105.7 to 124.4) | -35.1 (-38.5 to -31.9) | 65.6 (51.0 to 80.8) | -46.1 (-57.9 to -29.7) | 1636.0 (1316.7 to 1984.0) | -40.5 (-52.8 to -24.2) |
| Timor-Leste | 1058.5 (1016.0 to 1100.4) | 1.3 (-1.4 to 4.2) | 181.6 (166.1 to 199.3) | 2.9 (-2.5 to 8.6) | 175.0 (133.8 to 219.2) | -7.6 (-30.4 to 23.1) | 3749.5 (2901.8 to 4710.0) | -11.9 (-34.4 to 16.1) |
| Viet Nam | 1233.3 (1193.6 to 1277.2) | 1.7 (-1.4 to 5.2) | 203.4 (190.5 to 217.8) | -9.3 (-14.8 to -4.4) | 196.6 (164.2 to 226.8) | -14.3 (-33.4 to 7.4) | 3952.5 (3287.4 to 4604.9) | -17.4 (-36.5 to 5.5) |
| **South Asia** | **783.2 (732.0 to 843.7)** | **-8.2 (-10.0 to -6.5)** | **117.2 (106.3 to 128.2)** | **-22.2 (-24.7 to -19.5)** | **81.9 (74.7 to 89.9)** | **-23.1 (-32.4 to -12.4)** | **1805.1 (1659.8 to 1973.1)** | **-26.4 (-35.3 to -17.2)** |
| Afghanistan | 1098.9 (1050.0 to 1149.1) | -14.7 (-17.3 to -12.3) | 179.8 (165.6 to 195.7) | -21.6 (-25.5 to -17.5) | 183.9 (137.6 to 228.4) | -28.1 (-43.7 to -8.7) | 3982.5 (3016.5 to 4998.5) | -32.0 (-47.6 to -11.4) |
| Bangladesh | 986.0 (947.2 to 1028.7) | -10.5 (-12.9 to -7.9) | 167.3 (154.3 to 181.9) | -15.4 (-19.3 to -10.7) | 149.0 (122.6 to 179.4) | -26.7 (-42.0 to -6.7) | 2937.8 (2411.7 to 3565.4) | -35.8 (-49.2 to -18.1) |
| Bhutan | 784.0 (750.6 to 819.4) | -10.7 (-13.2 to -7.9) | 110.0 (100.6 to 120.0) | -22.0 (-26.2 to -16.9) | 71.2 (56.3 to 86.7) | -32.2 (-48.5 to -10.2) | 1476.9 (1184.8 to 1804.9) | -37.8 (-53.2 to -18.4) |
| India | 727.1 (674.6 to 788.5) | -8.0 (-10.0 to -6.0) | 107.4 (96.7 to 118.2) | -24.0 (-26.7 to -21.0) | 72.0 (64.7 to 79.7) | -22.8 (-33.8 to -10.0) | 1625.7 (1465.3 to 1788.7) | -26.1 (-36.5 to -14.8) |
| Iran | 934.3 (860.3 to 1015.7) | -14.8 (-17.4 to -11.9) | 100.8 (88.4 to 114.2) | -31.8 (-34.7 to -29.2) | 63.9 (56.8 to 69.7) | -48.8 (-53.3 to -43.1) | 1229.2 (1119.5 to 1332.6) | -50.2 (-54.5 to -45.2) |
| Maldives | 1013.5 (978.5 to 1052.2) | -40.2 (-41.8 to -38.4) | 131.1 (120.4 to 143.1) | -47.5 (-50.2 to -44.3) | 80.1 (66.6 to 92.8) | -64.7 (-71.3 to -57.1) | 1601.7 (1348.0 to 1849.8) | -70.1 (-75.6 to -63.6) |
| Nepal | 714.8 (683.4 to 749.8) | -14.1 (-16.6 to -11.4) | 117.3 (107.5 to 127.3) | -18.0 (-21.8 to -13.9) | 86.4 (69.9 to 108.3) | -32.0 (-47.8 to -10.6) | 1801.1 (1453.6 to 2269.3) | -36.9 (-51.8 to -18.3) |
| Pakistan | 1088.1 (1000.7 to 1188.9) | -5.6 (-8.2 to -2.8) | 152.8 (138.2 to 169.4) | -15.1 (-18.7 to -11.3) | 98.0 (81.1 to 119.4) | -8.1 (-25.0 to 14.7) | 2194.4 (1832.7 to 2676.9) | -8.5 (-25.2 to 14.2) |
| Sri Lanka | 1027.1 (989.2 to 1066.7) | -15.2 (-17.3 to -13.0) | 125.4 (113.8 to 137.3) | -24.9 (-28.5 to -20.7) | 110.7 (78.9 to 143.3) | -37.4 (-56.2 to -16.5) | 1992.1 (1450.5 to 2562.0) | -39.1 (-57.3 to -18.5) |
| Armenia | 990.2 (953.9 to 1029.4) | -16.6 (-19.3 to -13.6) | 111.4 (102.1 to 120.8) | -40.7 (-44.2 to -36.7) | 65.3 (57.3 to 72.9) | -45.0 (-50.8 to -38.4) | 1310.1 (1180.0 to 1461.3) | -45.3 (-50.8 to -39.0) |
| Azerbaijan | 1030.7 (987.3 to 1076.2) | -6.3 (-9.3 to -3.4) | 197.1 (182.4 to 213.8) | 6.1 (0.6 to 12.3) | 101.3 (85.3 to 120.1) | -20.4 (-34.1 to -2.6) | 2011.5 (1671.6 to 2369.7) | -27.4 (-40.7 to -10.5) |
| Bahrain | 736.5 (707.6 to 769.8) | -21.3 (-23.5 to -19.4) | 79.0 (72.4 to 86.5) | -35.5 (-39.2 to -31.4) | 81.5 (70.7 to 94.5) | -49.1 (-57.3 to -39.0) | 1384.2 (1212.7 to 1582.0) | -52.9 (-60.3 to -44.0) |
| Cyprus | 521.5 (495.7 to 553.5) | -39.2 (-41.4 to -36.5) | 67.5 (62.6 to 73.1) | -48.3 (-52.2 to -44.1) | 47.8 (40.1 to 55.4) | -73.1 (-78.1 to -66.9) | 684.9 (586.2 to 784.0) | -72.6 (-77.7 to -66.9) |
| Georgia | 1286.9 (1236.0 to 1342.6) | -5.7 (-8.3 to -3.0) | 212.6 (196.7 to 228.7) | -16.6 (-21.5 to -11.1) | 158.7 (141.1 to 176.1) | -17.4 (-26.3 to -7.9) | 3054.1 (2706.2 to 3392.2) | -23.2 (-32.1 to -12.9) |
| Iraq | 1284.1 (1231.3 to 1336.9) | -7.2 (-9.8 to -4.0) | 187.5 (170.1 to 207.1) | -8.1 (-14.0 to -1.9) | 170.0 (136.9 to 201.3) | -14.4 (-34.3 to 6.3) | 3324.6 (2661.1 to 3981.4) | -27.2 (-44.8 to -7.1) |
| Israel | 775.2 (749.6 to 803.5) | -29.6 (-31.4 to -27.6) | 65.1 (58.1 to 73.3) | -50.8 (-54.1 to -47.3) | 20.3 (17.3 to 22.2) | -67.2 (-70.1 to -64.7) | 426.5 (379.2 to 466.0) | -64.5 (-66.7 to -62.2) |
| Jordan | 1367.6 (1308.4 to 1427.4) | -4.6 (-7.5 to -1.1) | 158.5 (140.2 to 175.3) | -24.1 (-29.4 to -17.8) | 69.3 (55.8 to 84.0) | -53.7 (-63.8 to -40.2) | 1383.1 (1147.4 to 1636.3) | -55.8 (-65.0 to -43.8) |
| Kuwait | 1077.2 (1038.0 to 1116.0) | -8.0 (-10.6 to -5.2) | 101.0 (91.4 to 110.9) | -9.2 (-15.1 to -3.7) | 34.0 (28.0 to 40.8) | -32.7 (-43.0 to -20.4) | 754.7 (635.5 to 879.2) | -36.9 (-45.8 to -27.0) |
| Lebanon | 1086.7 (1037.8 to 1135.9) | -1.5 (-4.1 to 1.2) | 116.9 (105.3 to 129.1) | -24.1 (-28.5 to -19.2) | 44.5 (36.8 to 52.4) | -68.6 (-76.3 to -59.3) | 917.0 (790.1 to 1059.9) | -68.9 (-76.4 to -60.0) |
| Oman | 1169.9 (1128.2 to 1214.3) | -4.7 (-7.8 to -1.6) | 149.6 (135.4 to 163.5) | -13.1 (-19.4 to -6.0) | 74.2 (60.0 to 87.0) | -41.3 (-55.9 to -18.5) | 1470.8 (1215.6 to 1716.3) | -48.3 (-61.1 to -28.6) |
| Palestine | 863.4 (826.6 to 900.8) | -12.4 (-15.4 to -9.5) | 158.0 (144.9 to 171.4) | -13.4 (-18.3 to -8.2) | 114.0 (98.2 to 129.3) | -42.2 (-53.3 to -28.8) | 1991.7 (1731.9 to 2243.3) | -45.6 (-55.9 to -32.5) |
| Qatar | 1012.2 (972.7 to 1054.2) | -37.1 (-38.9 to -35.4) | 110.6 (101.0 to 122.6) | -36.7 (-40.8 to -33.1) | 54.8 (43.6 to 66.7) | -65.4 (-73.3 to -56.1) | 1004.2 (818.8 to 1201.4) | -65.8 (-73.5 to -56.8) |
| Saudi Arabia | 965.2 (924.9 to 1006.4) | -1.8 (-4.9 to 1.2) | 130.7 (119.4 to 141.7) | -21.4 (-26.5 to -16.4) | 99.5 (83.5 to 120.4) | -38.5 (-53.2 to -17.3) | 2023.3 (1687.1 to 2452.5) | -39.5 (-53.4 to -19.3) |
| Syrian Arab Republic | 1044.3 (1002.1 to 1086.5) | -24.3 (-26.2 to -22.5) | 135.2 (124.7 to 146.2) | -25.7 (-29.8 to -22.0) | 106.3 (84.0 to 130.9) | -29.2 (-47.1 to -2.2) | 2124.4 (1670.8 to 2652.8) | -40.1 (-55.6 to -17.1) |
| Turkey | 999.1 (963.3 to 1039.7) | -30.1 (-31.9 to -28.2) | 110.4 (99.8 to 121.8) | -37.9 (-41.8 to -33.3) | 72.1 (59.5 to 85.7) | -50.2 (-59.8 to -37.5) | 1343.0 (1130.4 to 1571.1) | -56.9 (-65.2 to -46.7) |
| United Arab Emirates | 1429.4 (1361.6 to 1493.0) | -15.8 (-18.8 to -12.9) | 173.5 (157.5 to 191.8) | -28.1 (-32.8 to -23.6) | 88.8 (72.6 to 105.9) | -34.8 (-46.1 to -21.7) | 1648.8 (1364.3 to 1951.6) | -43.6 (-53.2 to -32.2) |
| Yemen | 963.6 (916.7 to 1009.1) | -8.7 (-11.5 to -5.7) | 157.2 (143.8 to 171.5) | -15.9 (-20.7 to -10.8) | 165.1 (124.3 to 222.4) | -25.2 (-44.1 to 0.4) | 3331.1 (2501.0 to 4441.5) | -31.6 (-48.8 to -7.1) |

ASR: Age-standardized rate; UI: Uncertainty interval; DALYs: Disability-adjusted life years

| **Table S7. Atrial fibrillation and flutter burden by country.** | | | | | | | | |
| --- | --- | --- | --- | --- | --- | --- | --- | --- |
|  | **Prevalence (95%UI)** | | **Incidence (95%UI)** | | **Death (95%UI)** | | **DALYs (95%UI)** | |
| **Location** | **ASR (per 100,000) 2021** | **Percent change, 1990-2021** | **ASR (per 100,000) 2021** | **Percent change, 1990-2021** | **ASR (per 100,000) 2021** | **Percent change, 1990-2021** | **ASR (per 100,000) 2021** | **Percent change, 1990-2021** |
| **Central Asia** | **536.7 (410.8 to 698.7)** | **4.5 (2.3 to 7.4)** | **44.5 (33.5 to 57.8)** | **1.1 (-1.1 to 3.7)** | **2.4 (2.1 to 2.6)** | **26.9 (7.3 to 47.5)** | **75.0 (59.9 to 95.5)** | **13.2 (6.3 to 21.8)** |
| Kazakhstan | 589.3 (453.4 to 768.6) | 4.7 (0.2 to 10.2) | 48.9 (37.1 to 63.7) | 1.4 (-3.4 to 6.3) | 3.0 (2.6 to 3.3) | 34.9 (3.0 to 72.6) | 85.4 (68.1 to 108.7) | 14.6 (2.4 to 29.8) |
| Kyrgyzstan | 491.2 (377.3 to 639.2) | 2.6 (-2.3 to 7.4) | 41.1 (31.4 to 53.8) | -0.8 (-5.2 to 3.7) | 2.2 (1.8 to 2.5) | 8.2 (-13.4 to 33.2) | 69.1 (54.4 to 88.3) | 6.4 (-4.1 to 18.5) |
| Tajikistan | 489.5 (373.7 to 641.5) | 2.7 (-1.8 to 7.9) | 40.8 (30.5 to 53.3) | -0.6 (-5.0 to 4.0) | 1.2 (0.9 to 1.4) | -8.7 (-39.9 to 27.6) | 54.3 (39.7 to 72.3) | -1.4 (-13.6 to 8.9) |
| Turkmenistan | 563.7 (432.8 to 729.2) | 12.4 (7.2 to 18.6) | 47.0 (35.5 to 61.0) | 8.8 (4.3 to 14.9) | 2.9 (2.3 to 3.8) | 32.1 (1.3 to 67.6) | 86.5 (67.7 to 106.7) | 22.8 (7.3 to 40.5) |
| Uzbekistan | 496.6 (380.6 to 651.6) | 7.9 (2.4 to 14.4) | 41.5 (30.9 to 54.3) | 4.7 (-0.7 to 10.8) | 1.4 (1.2 to 1.6) | 35.7 (-22.2 to 138.0) | 59.0 (44.7 to 77.9) | 17.5 (-0.9 to 39.6) |
| **East Asia** | **526.4 (420.6 to 683.6)** | **13.8 (10.2 to 17.8)** | **45.1 (35.1 to 59.6)** | **5.0 (1.2 to 8.7)** | **4.3 (3.4 to 5.2)** | **-12.9 (-34.3 to 12.6)** | **89.8 (72.4 to 109.6)** | **-4.3 (-19.3 to 13.0)** |
| China | 524.0 (418.1 to 681.2) | 14.5 (10.8 to 18.8) | 44.9 (35.0 to 59.4) | 5.4 (1.4 to 9.3) | 4.3 (3.4 to 5.2) | -12.3 (-34.6 to 15.6) | 89.8 (72.1 to 109.7) | -3.8 (-19.4 to 14.2) |
| Japan | 391.9 (310.6 to 511.2) | -24.6 (-26.8 to -22.9) | 31.9 (24.8 to 41.9) | -24.2 (-26.1 to -22.5) | 2.3 (1.9 to 2.6) | -23.5 (-29.4 to -19.6) | 61.8 (50.4 to 76.4) | -24.1 (-26.6 to -22.0) |
| Mongolia | 544.6 (413.5 to 704.4) | 7.3 (2.2 to 13.1) | 45.6 (34.3 to 60.1) | 3.5 (-1.0 to 9.0) | 1.9 (1.5 to 2.4) | -4.6 (-28.4 to 24.9) | 69.4 (52.4 to 89.8) | 0.1 (-11.6 to 12.5) |
| North Korea | 532.4 (422.7 to 687.4) | -0.3 (-4.8 to 4.5) | 48.1 (37.2 to 63.3) | -3.2 (-7.5 to 0.7) | 5.1 (3.8 to 7.7) | 0.5 (-23.5 to 37.4) | 104.0 (80.3 to 138.0) | 0.1 (-14.3 to 19.1) |
| South Korea | 675.1 (584.7 to 790.6) | 10.6 (-4.1 to 27.9) | 47.2 (39.3 to 57.5) | -4.4 (-16.4 to 8.9) | 3.5 (2.6 to 4.2) | 3.6 (-50.0 to 45.8) | 96.3 (76.8 to 118.0) | 2.6 (-28.0 to 26.7) |
| Taiwan [Province of China] | 629.8 (513.3 to 784.2) | -2.4 (-8.3 to 4.1) | 53.2 (42.4 to 67.1) | -3.2 (-9.4 to 3.3) | 3.7 (3.1 to 4.1) | -29.1 (-35.3 to -23.9) | 92.1 (74.5 to 114.0) | -17.9 (-22.6 to -13.8) |
| **Southeast Asia** | **662.3 (519.6 to 854.4)** | **3.9 (2.7 to 5.3)** | **59.7 (45.6 to 79.3)** | **1.5 (0.0 to 3.0)** | **5.3 (4.4 to 6.1)** | **24.5 (-1.8 to 53.7)** | **115.4 (95.9 to 139.1)** | **12.8 (-1.4 to 26.8)** |
| Brunei Darussalam | 452.0 (354.8 to 583.0) | -15.9 (-19.4 to -11.4) | 35.5 (27.3 to 45.6) | -17.7 (-21.5 to -13.7) | 6.2 (5.2 to 7.4) | -13.0 (-32.0 to 19.0) | 122.0 (102.5 to 144.0) | -15.6 (-28.9 to 2.6) |
| Cambodia | 578.6 (457.2 to 751.3) | 2.8 (-0.3 to 6.5) | 52.9 (40.2 to 69.7) | -0.6 (-4.2 to 2.9) | 4.3 (3.3 to 5.6) | 35.1 (1.3 to 75.6) | 98.5 (76.5 to 122.3) | 14.1 (-3.3 to 33.0) |
| Indonesia | 728.2 (569.7 to 942.3) | 3.9 (2.4 to 5.7) | 66.4 (50.8 to 87.9) | 3.0 (1.4 to 4.5) | 6.4 (4.8 to 7.8) | 76.6 (26.0 to 134.8) | 133.1 (106.8 to 160.6) | 31.0 (11.4 to 52.8) |
| Lao PDR | 605.1 (475.9 to 782.3) | 3.0 (-1.2 to 7.1) | 55.0 (41.9 to 72.8) | -1.4 (-5.3 to 2.6) | 5.1 (4.0 to 6.7) | 22.6 (-9.4 to 73.6) | 111.2 (87.6 to 138.4) | 8.6 (-10.0 to 32.3) |
| Malaysia | 673.2 (526.9 to 873.0) | 6.6 (2.0 to 10.8) | 58.7 (44.4 to 78.7) | 2.8 (-1.6 to 7.1) | 4.9 (4.1 to 5.7) | 53.7 (22.9 to 100.0) | 112.6 (91.2 to 135.6) | 22.0 (9.5 to 39.4) |
| Myanmar | 614.4 (480.4 to 795.8) | 0.1 (-4.0 to 4.7) | 55.7 (42.1 to 74.5) | -3.0 (-7.3 to 1.1) | 5.1 (3.9 to 6.6) | 23.8 (-13.4 to 72.0) | 111.5 (88.0 to 140.6) | 8.1 (-13.6 to 30.6) |
| Philippines | 653.7 (513.1 to 843.1) | 0.8 (0.1 to 1.8) | 60.3 (45.8 to 80.2) | 1.1 (0.3 to 2.0) | 4.8 (4.0 to 5.8) | -8.4 (-24.7 to 6.9) | 111.0 (89.2 to 135.2) | -0.7 (-10.7 to 8.9) |
| Singapore | 430.3 (339.4 to 557.4) | -13.3 (-16.5 to -9.7) | 33.1 (25.7 to 43.0) | -16.7 (-20.0 to -12.8) | 1.2 (1.0 to 1.3) | -37.0 (-42.9 to -32.4) | 50.5 (38.4 to 65.6) | -26.8 (-32.0 to -22.4) |
| Thailand | 603.8 (470.5 to 784.5) | 4.6 (0.4 to 8.9) | 53.2 (40.5 to 70.8) | 1.0 (-3.8 to 5.6) | 4.6 (3.4 to 5.8) | -21.5 (-44.4 to 9.7) | 101.0 (80.1 to 124.4) | -10.4 (-28.9 to 8.0) |
| Timor-Leste | 605.8 (476.4 to 787.0) | 3.4 (-1.8 to 9.2) | 54.6 (41.8 to 72.3) | 0.2 (-4.4 to 5.3) | 4.5 (3.5 to 5.8) | 27.7 (-11.1 to 94.2) | 102.4 (80.7 to 131.2) | 12.9 (-6.9 to 38.4) |
| Viet Nam | 644.9 (502.7 to 844.1) | 10.8 (5.9 to 15.7) | 57.3 (43.8 to 76.7) | 6.2 (1.3 to 10.8) | 5.8 (4.4 to 7.4) | 40.8 (5.0 to 86.9) | 118.9 (91.5 to 147.6) | 23.9 (4.8 to 47.8) |
| **South Asia** | **530.9 (410.2 to 696.7)** | **2.2 (1.2 to 3.1)** | **51.0 (38.1 to 67.9)** | **1.8 (0.9 to 2.7)** | **3.7 (2.7 to 4.7)** | **55.1 (14.8 to 111.4)** | **88.3 (68.0 to 111.4)** | **23.0 (5.9 to 43.0)** |
| Afghanistan | 338.8 (257.5 to 442.8) | 8.3 (3.9 to 13.1) | 33.3 (24.5 to 44.5) | 4.3 (-0.1 to 8.7) | 3.3 (2.0 to 4.9) | 19.4 (-7.0 to 61.0) | 72.1 (51.5 to 95.1) | 14.3 (-6.2 to 37.4) |
| Bangladesh | 494.7 (380.7 to 643.9) | 4.8 (0.2 to 9.5) | 46.2 (34.3 to 61.3) | 1.4 (-3.7 to 6.4) | 5.0 (3.6 to 7.0) | 53.3 (13.6 to 136.0) | 97.3 (74.5 to 130.0) | 23.7 (2.9 to 52.3) |
| Bhutan | 492.9 (384.1 to 647.5) | 5.5 (0.9 to 10.2) | 46.3 (34.6 to 61.8) | 1.5 (-3.1 to 5.9) | 4.5 (3.1 to 6.3) | 69.4 (19.0 to 147.9) | 93.8 (70.8 to 121.0) | 31.0 (6.8 to 59.2) |
| India | 533.9 (412.7 to 700.0) | 2.4 (1.3 to 3.4) | 51.4 (38.4 to 68.5) | 2.3 (1.2 to 3.2) | 3.4 (2.5 to 4.3) | 60.2 (14.8 to 118.1) | 86.1 (65.7 to 108.5) | 24.1 (5.9 to 43.8) |
| Iran | 425.4 (327.2 to 559.2) | 10.2 (8.6 to 11.7) | 40.6 (30.0 to 54.4) | 8.6 (6.9 to 10.2) | 3.3 (2.5 to 3.8) | -0.4 (-22.3 to 28.3) | 72.4 (57.4 to 88.3) | 2.6 (-13.1 to 18.2) |
| Maldives | 619.1 (486.1 to 803.1) | 2.9 (-2.2 to 8.2) | 54.1 (41.3 to 71.7) | 0.8 (-3.9 to 6.1) | 4.1 (3.0 to 5.1) | 0.4 (-34.4 to 55.2) | 93.3 (73.3 to 118.4) | -4.2 (-24.4 to 18.5) |
| Nepal | 471.7 (359.9 to 618.9) | -0.0 (-4.3 to 4.3) | 44.6 (33.4 to 59.3) | -2.8 (-7.0 to 1.1) | 4.1 (2.8 to 5.8) | 75.5 (26.1 to 159.8) | 87.3 (65.7 to 116.3) | 30.5 (10.1 to 57.6) |
| Pakistan | 558.6 (429.5 to 735.7) | 0.7 (-2.6 to 4.0) | 53.8 (40.3 to 71.6) | 1.1 (-2.0 to 4.5) | 4.6 (3.3 to 6.4) | 63.6 (22.5 to 126.3) | 101.5 (79.7 to 131.7) | 27.6 (10.2 to 50.8) |
| Sri Lanka | 632.2 (494.6 to 817.1) | 5.3 (1.3 to 9.5) | 55.7 (42.4 to 73.3) | 3.1 (-1.0 to 8.5) | 4.1 (2.9 to 5.4) | 11.3 (-23.6 to 53.9) | 95.6 (72.0 to 120.6) | 5.8 (-11.5 to 27.6) |
| Armenia | 556.6 (424.7 to 729.8) | 9.6 (4.9 to 14.4) | 45.7 (34.2 to 60.6) | 6.1 (1.4 to 11.0) | 2.5 (2.2 to 2.9) | 66.7 (32.2 to 100.8) | 76.9 (59.8 to 96.1) | 27.3 (16.1 to 41.3) |
| Azerbaijan | 540.9 (415.4 to 701.5) | 7.8 (3.0 to 13.5) | 44.9 (33.8 to 59.0) | 3.6 (-1.1 to 9.2) | 1.8 (1.4 to 2.2) | 8.4 (-22.4 to 44.6) | 67.9 (51.4 to 90.1) | 8.7 (-3.2 to 23.1) |
| Bahrain | 386.7 (293.8 to 505.6) | 7.0 (1.8 to 11.5) | 36.3 (26.6 to 48.2) | 2.7 (-2.5 to 7.1) | 5.7 (2.6 to 7.9) | -18.7 (-39.0 to -0.7) | 94.9 (57.6 to 122.8) | -16.1 (-32.7 to -2.0) |
| Cyprus | 487.2 (395.5 to 615.6) | -25.3 (-33.0 to -17.4) | 43.0 (34.0 to 55.3) | -23.0 (-30.6 to -13.9) | 8.4 (6.8 to 9.9) | -40.7 (-56.4 to -12.8) | 129.8 (108.8 to 154.1) | -40.4 (-53.5 to -20.1) |
| Georgia | 568.7 (436.2 to 738.6) | 1.8 (-2.5 to 6.4) | 47.2 (35.9 to 61.5) | -0.2 (-4.3 to 4.2) | 5.0 (4.4 to 5.6) | 54.6 (21.4 to 115.7) | 122.4 (102.9 to 145.3) | 36.9 (20.1 to 66.1) |
| Iraq | 406.1 (308.5 to 532.0) | 8.8 (3.7 to 14.3) | 38.4 (28.1 to 51.2) | 4.9 (-0.8 to 11.2) | 4.9 (3.5 to 6.2) | 49.1 (5.9 to 132.1) | 93.6 (74.2 to 114.0) | 22.5 (-2.4 to 65.8) |
| Israel | 1155.5 (958.3 to 1312.4) | 36.2 (13.9 to 72.9) | 92.0 (75.2 to 105.2) | 29.8 (7.6 to 63.2) | 4.8 (4.0 to 5.4) | -23.3 (-29.8 to -17.6) | 148.8 (117.3 to 184.4) | 2.5 (-7.1 to 15.4) |
| Jordan | 396.6 (300.6 to 520.3) | 12.0 (7.5 to 18.1) | 37.0 (27.5 to 49.7) | 7.7 (3.2 to 13.1) | 2.7 (2.1 to 3.3) | -13.2 (-36.0 to 9.6) | 63.1 (49.4 to 78.3) | -8.2 (-23.8 to 6.1) |
| Kuwait | 408.1 (310.6 to 530.2) | 10.3 (4.9 to 14.9) | 37.9 (27.9 to 50.9) | 6.8 (1.3 to 11.6) | 3.0 (2.4 to 3.6) | 15.9 (-1.2 to 32.8) | 66.6 (52.8 to 82.2) | 6.9 (-2.9 to 17.4) |
| Lebanon | 387.1 (298.5 to 511.1) | 13.1 (7.2 to 18.4) | 36.2 (27.0 to 48.3) | 8.7 (2.8 to 14.3) | 3.4 (2.8 to 4.4) | -31.3 (-57.6 to 33.1) | 68.9 (56.3 to 84.2) | -25.9 (-49.2 to 11.9) |
| Oman | 365.3 (276.4 to 482.9) | 24.7 (18.0 to 30.3) | 34.4 (25.3 to 46.3) | 18.8 (12.6 to 25.3) | 4.7 (3.6 to 5.9) | 17.4 (-22.7 to 82.3) | 91.0 (74.6 to 109.2) | 13.7 (-17.0 to 54.3) |
| Palestine | 347.4 (265.2 to 454.0) | 7.5 (3.0 to 12.1) | 33.2 (24.4 to 44.7) | 3.8 (-0.3 to 8.7) | 5.4 (4.3 to 6.7) | 5.1 (-20.0 to 43.1) | 89.8 (74.3 to 107.6) | 3.1 (-16.6 to 29.1) |
| Qatar | 402.6 (305.8 to 529.3) | 10.8 (5.6 to 15.8) | 37.6 (27.6 to 50.6) | 6.8 (0.8 to 11.9) | 4.1 (1.5 to 6.0) | -44.9 (-61.9 to -27.3) | 78.5 (45.5 to 103.7) | -32.5 (-47.4 to -15.0) |
| Saudi Arabia | 391.9 (301.9 to 511.7) | 18.5 (13.7 to 25.0) | 36.9 (27.2 to 49.8) | 13.0 (8.1 to 19.3) | 3.4 (2.7 to 4.1) | 12.7 (-22.0 to 54.4) | 72.2 (58.2 to 88.6) | 12.8 (-11.0 to 39.4) |
| Syrian Arab Republic | 377.8 (288.1 to 497.3) | 8.5 (3.9 to 12.8) | 35.3 (25.7 to 47.1) | 3.4 (-1.0 to 7.6) | 4.6 (2.9 to 5.9) | 7.4 (-28.3 to 59.2) | 85.7 (63.8 to 105.2) | 7.3 (-15.0 to 39.0) |
| Turkey | 282.9 (252.2 to 314.7) | -15.0 (-30.8 to 3.6) | 27.3 (24.1 to 31.3) | -14.6 (-31.1 to 3.7) | 3.9 (3.0 to 4.7) | 8.2 (-22.4 to 46.4) | 67.9 (56.4 to 80.4) | -5.7 (-26.2 to 16.4) |
| United Arab Emirates | 424.7 (326.5 to 554.8) | 11.3 (6.2 to 16.8) | 39.7 (29.2 to 52.8) | 7.1 (2.4 to 13.0) | 3.8 (2.5 to 4.9) | 5.6 (-30.4 to 47.1) | 85.8 (66.1 to 105.2) | 7.9 (-15.2 to 30.2) |
| Yemen | 337.2 (255.8 to 443.6) | 9.7 (4.9 to 16.2) | 32.9 (24.2 to 44.1) | 6.0 (0.6 to 11.6) | 3.7 (2.6 to 5.1) | 21.6 (-17.1 to 84.4) | 75.2 (55.6 to 97.4) | 14.7 (-11.0 to 49.4) |

ASR: Age-standardized rate; UI: Uncertainty interval; DALYs: Disability-adjusted life year

| **Table S8. Hypertensive heart disease burden by country.** | | | | | | |
| --- | --- | --- | --- | --- | --- | --- |
|  | **Prevalence (95%UI)** | | **Death (95%UI)** | | **DALYs (95%UI)** | |
| **Location** | **ASR (per 100,000) 2021** | **Percent change, 1990-2021** | **ASR (per 100,000) 2021** | **Percent change, 1990-2021** | **ASR (per 100,000) 2021** | **Percent change, 1990-2021** |
| **Central Asia** | **95.6 (64.6 to 132.6)** | **19.3 (3.8 to 33.9)** | **20.6 (17.7 to 23.9)** | **28.5 (1.4 to 57.4)** | **376.1 (320.9 to 441.9)** | **16.1 (-5.1 to 40.5)** |
| Kazakhstan | 58.7 (39.0 to 84.0) | -11.4 (-28.0 to 7.6) | 9.2 (7.5 to 11.0) | -5.3 (-25.8 to 21.2) | 173.9 (140.6 to 211.2) | -21.9 (-39.3 to 1.1) |
| Kyrgyzstan | 92.8 (62.8 to 127.4) | 49.1 (27.5 to 77.3) | 17.7 (14.9 to 21.1) | 56.6 (23.0 to 96.5) | 344.1 (289.6 to 409.5) | 43.9 (15.7 to 79.3) |
| Tajikistan | 163.2 (112.0 to 221.0) | -4.1 (-18.4 to 14.0) | 33.5 (23.5 to 47.4) | -25.8 (-53.7 to 25.9) | 591.3 (410.1 to 820.4) | -28.1 (-54.3 to 16.4) |
| Turkmenistan | 82.0 (55.0 to 116.0) | 31.0 (11.2 to 54.6) | 21.0 (15.3 to 30.0) | 22.5 (-14.3 to 74.9) | 421.9 (305.9 to 605.6) | 20.4 (-16.1 to 72.7) |
| Uzbekistan | 87.8 (59.8 to 125.3) | 19.3 (-0.8 to 40.5) | 19.6 (14.8 to 26.1) | 40.9 (-9.0 to 108.6) | 389.9 (286.2 to 517.1) | 32.5 (-9.6 to 90.6) |
| **East Asia** | **193.2 (147.4 to 245.9)** | **-10.9 (-19.2 to -3.3)** | **18.7 (13.0 to 24.1)** | **-55.3 (-66.5 to -34.4)** | **292.6 (210.4 to 371.8)** | **-58.5 (-68.0 to -37.8)** |
| China | 192.5 (146.7 to 245.0) | -11.8 (-20.3 to -4.3) | 18.8 (12.9 to 24.5) | -55.8 (-67.2 to -34.7) | 292.5 (208.2 to 374.2) | -59.1 (-68.9 to -38.3) |
| Japan | 44.9 (33.6 to 80.5) | -7.8 (-19.1 to 4.7) | 2.6 (2.0 to 2.9) | -73.2 (-75.5 to -71.8) | 41.0 (35.5 to 44.6) | -70.3 (-71.7 to -69.0) |
| Mongolia | 51.9 (34.1 to 75.7) | -3.1 (-20.8 to 18.3) | 9.0 (6.0 to 12.9) | -34.7 (-61.9 to 13.6) | 167.9 (114.3 to 236.2) | -38.7 (-64.0 to 5.3) |
| North Korea | 181.4 (140.0 to 237.9) | 13.2 (-1.8 to 29.0) | 26.8 (19.3 to 36.2) | -9.0 (-36.9 to 61.2) | 465.3 (336.7 to 611.3) | -7.8 (-36.7 to 64.9) |
| South Korea | 103.0 (81.1 to 130.0) | -4.4 (-19.6 to 14.8) | 5.6 (4.0 to 8.7) | -68.8 (-77.4 to -36.8) | 75.4 (58.5 to 119.5) | -73.5 (-80.0 to -41.3) |
| Taiwan [Province of China] | 230.6 (178.4 to 301.0) | 17.3 (4.8 to 32.1) | 10.9 (9.4 to 12.0) | -54.1 (-58.4 to -50.0) | 198.0 (177.8 to 214.3) | -51.1 (-55.3 to -47.3) |
| **Southeast Asia** | **163.3 (131.2 to 205.2)** | **-2.8 (-9.5 to 3.6)** | **22.9 (16.9 to 26.5)** | **-22.5 (-35.8 to -0.6)** | **464.8 (339.7 to 536.9)** | **-24.0 (-36.4 to -1.0)** |
| Brunei Darussalam | 57.0 (36.2 to 58.3) | -1.8 (-14.7 to 17.1) | 14.4 (11.6 to 17.8) | -36.4 (-51.8 to -14.5) | 223.3 (182.3 to 284.7) | -39.5 (-54.7 to -14.9) |
| Cambodia | 196.7 (153.5 to 254.2) | 1.5 (-8.2 to 13.8) | 32.4 (20.3 to 41.7) | -24.2 (-45.8 to 12.4) | 631.4 (392.2 to 834.5) | -30.4 (-51.5 to 5.0) |
| Indonesia | 190.4 (148.5 to 240.8) | 0.5 (-7.7 to 7.3) | 32.9 (21.4 to 42.3) | 1.6 (-21.7 to 38.7) | 632.3 (405.4 to 817.6) | -7.4 (-27.7 to 26.2) |
| Lao PDR | 170.1 (130.0 to 221.1) | 3.5 (-8.8 to 17.7) | 34.9 (21.8 to 46.5) | -38.0 (-56.8 to 24.7) | 690.3 (427.3 to 933.1) | -42.9 (-61.7 to 22.5) |
| Malaysia | 58.9 (45.2 to 76.7) | -7.9 (-20.4 to 6.3) | 5.9 (5.0 to 7.2) | -36.1 (-50.0 to -5.1) | 118.9 (101.2 to 140.7) | -38.2 (-51.3 to -11.5) |
| Myanmar | 162.7 (126.6 to 208.3) | -0.1 (-11.3 to 12.9) | 28.8 (15.1 to 41.9) | -37.7 (-55.2 to 2.9) | 575.3 (305.5 to 831.1) | -42.4 (-58.8 to -4.6) |
| Philippines | 200.0 (157.7 to 249.9) | 4.3 (-2.3 to 10.3) | 35.6 (29.6 to 42.0) | -3.6 (-18.9 to 14.6) | 738.9 (618.4 to 869.2) | 1.2 (-15.9 to 21.0) |
| Singapore | 114.5 (87.3 to 147.9) | 46.1 (27.3 to 74.7) | 6.4 (5.6 to 7.0) | -60.8 (-64.5 to -57.9) | 126.1 (113.5 to 136.6) | -57.7 (-61.5 to -54.3) |
| Thailand | 82.7 (64.3 to 108.5) | 30.2 (15.2 to 48.6) | 3.3 (2.5 to 4.4) | -31.4 (-51.4 to 8.4) | 68.3 (53.3 to 86.0) | -28.0 (-47.1 to 7.4) |
| Timor-Leste | 178.2 (139.9 to 230.1) | -7.0 (-17.0 to 5.8) | 32.9 (21.2 to 45.1) | -16.4 (-42.0 to 30.0) | 648.1 (421.2 to 884.4) | -18.4 (-44.8 to 39.3) |
| Viet Nam | 205.7 (159.8 to 267.4) | -4.2 (-15.2 to 10.1) | 22.6 (14.9 to 31.4) | -29.2 (-51.0 to 4.1) | 410.4 (269.0 to 590.4) | -33.5 (-53.9 to -2.0) |
| **South Asia** | **111.1 (86.6 to 142.7)** | **6.7 (-1.1 to 13.8)** | **16.5 (13.1 to 21.4)** | **-7.7 (-30.2 to 38.3)** | **301.7 (238.1 to 392.3)** | **-12.6 (-33.8 to 31.4)** |
| Afghanistan | 252.6 (194.6 to 323.8) | -3.2 (-14.1 to 9.5) | 78.3 (43.8 to 115.0) | -21.7 (-40.1 to 9.7) | 1501.8 (790.5 to 2264.0) | -24.8 (-43.6 to 6.3) |
| Bangladesh | 139.5 (108.7 to 178.4) | 5.7 (-7.3 to 19.4) | 20.7 (12.9 to 44.9) | -19.4 (-49.7 to 36.0) | 340.7 (213.7 to 742.7) | -28.1 (-55.5 to 25.5) |
| Bhutan | 132.9 (104.2 to 168.2) | 0.9 (-10.4 to 13.9) | 18.7 (12.7 to 24.1) | -22.8 (-43.7 to 18.7) | 318.8 (215.2 to 413.2) | -29.5 (-49.4 to 7.9) |
| India | 106.2 (82.0 to 137.4) | 8.5 (-0.3 to 16.5) | 15.3 (11.6 to 19.6) | -4.6 (-29.2 to 50.9) | 284.0 (213.8 to 364.7) | -10.8 (-33.6 to 39.8) |
| Iran | 250.2 (196.9 to 312.9) | 10.7 (2.9 to 17.9) | 28.9 (24.2 to 34.9) | -30.9 (-44.5 to -12.9) | 495.3 (420.8 to 571.8) | -33.6 (-46.8 to -16.6) |
| Maldives | 130.8 (99.7 to 170.1) | 14.3 (-1.3 to 31.4) | 9.2 (7.2 to 11.4) | -54.9 (-71.1 to 5.2) | 167.3 (134.1 to 205.9) | -60.0 (-74.2 to 1.8) |
| Nepal | 121.8 (95.0 to 156.7) | 20.4 (4.9 to 36.7) | 21.4 (15.7 to 29.6) | -0.7 (-31.0 to 61.9) | 369.5 (270.4 to 507.1) | -8.1 (-36.7 to 49.5) |
| Pakistan | 125.1 (94.9 to 159.1) | 4.5 (-5.6 to 13.8) | 23.4 (17.0 to 32.0) | 7.2 (-13.3 to 39.0) | 421.0 (304.7 to 575.2) | 4.3 (-16.0 to 34.7) |
| Sri Lanka | 164.8 (128.8 to 213.0) | -4.6 (-15.7 to 8.1) | 15.6 (10.1 to 23.9) | -52.2 (-70.1 to -14.6) | 300.3 (193.4 to 462.6) | -51.8 (-70.2 to -12.8) |
| Armenia | 127.8 (97.5 to 163.3) | 33.4 (12.6 to 58.6) | 15.0 (12.0 to 18.6) | -21.7 (-44.7 to 9.1) | 245.3 (196.3 to 312.9) | -24.7 (-46.4 to 6.6) |
| Azerbaijan | 98.3 (66.7 to 134.8) | -1.0 (-16.6 to 16.8) | 20.4 (14.1 to 27.7) | -25.7 (-56.5 to 17.6) | 375.7 (261.3 to 521.0) | -27.1 (-54.1 to 17.1) |
| Bahrain | 180.7 (139.3 to 231.8) | 29.1 (14.3 to 46.3) | 25.7 (19.6 to 32.3) | -35.1 (-52.1 to -9.2) | 391.7 (294.9 to 502.3) | -38.9 (-54.8 to -13.0) |
| Cyprus | 67.4 (48.9 to 92.3) | 18.5 (1.2 to 43.8) | 15.3 (11.6 to 18.7) | -51.6 (-68.7 to 1.5) | 179.0 (144.3 to 214.6) | -55.1 (-70.1 to -6.2) |
| Georgia | 152.2 (79.0 to 223.7) | 94.4 (33.7 to 138.4) | 44.8 (39.1 to 50.2) | 242.7 (177.6 to 331.5) | 759.2 (664.7 to 856.7) | 183.7 (127.7 to 261.0) |
| Iraq | 194.9 (151.4 to 253.3) | -5.7 (-17.1 to 6.7) | 34.1 (26.1 to 42.6) | -8.7 (-35.7 to 42.0) | 585.6 (444.4 to 740.3) | -19.3 (-43.8 to 31.7) |
| Israel | 35.9 (26.3 to 48.5) | 46.1 (21.7 to 76.2) | 2.4 (2.0 to 2.6) | -57.4 (-62.1 to -53.8) | 34.5 (30.1 to 37.7) | -58.7 (-62.3 to -55.7) |
| Jordan | 341.9 (268.8 to 427.8) | 11.8 (-2.0 to 25.8) | 37.2 (28.9 to 46.8) | -35.2 (-53.7 to -6.6) | 631.5 (490.8 to 792.3) | -39.6 (-57.6 to -11.7) |
| Kuwait | 286.2 (222.3 to 367.5) | -6.6 (-17.8 to 5.3) | 17.0 (13.3 to 21.2) | -59.4 (-67.1 to -50.4) | 279.9 (223.9 to 352.8) | -63.7 (-70.5 to -55.4) |
| Lebanon | 184.8 (142.4 to 236.4) | -4.6 (-16.0 to 7.5) | 12.3 (9.8 to 15.1) | -60.8 (-75.1 to -7.3) | 201.6 (165.8 to 244.3) | -63.5 (-77.0 to -13.6) |
| Oman | 256.0 (199.5 to 332.8) | 31.6 (15.1 to 51.6) | 43.9 (32.5 to 55.2) | -11.7 (-45.8 to 35.1) | 761.6 (566.7 to 962.2) | -19.0 (-51.4 to 26.5) |
| Palestine | 234.1 (184.0 to 299.1) | 0.5 (-10.4 to 12.1) | 38.9 (31.1 to 47.0) | -35.9 (-51.9 to -3.0) | 615.1 (500.9 to 736.0) | -39.2 (-55.4 to -4.7) |
| Qatar | 174.5 (133.7 to 227.2) | 21.4 (6.2 to 38.2) | 14.3 (10.4 to 18.5) | -59.9 (-70.4 to -46.7) | 226.6 (169.4 to 301.9) | -59.9 (-70.5 to -45.5) |
| Saudi Arabia | 289.1 (222.8 to 372.7) | 3.3 (-10.4 to 15.6) | 50.4 (28.8 to 69.8) | -19.4 (-46.8 to 16.0) | 927.3 (526.7 to 1308.3) | -19.1 (-46.5 to 18.4) |
| Syrian Arab Republic | 178.9 (135.2 to 235.9) | -5.2 (-18.9 to 7.8) | 38.9 (29.6 to 51.7) | -21.8 (-44.7 to 8.7) | 629.7 (467.3 to 857.0) | -26.7 (-49.8 to 8.4) |
| Turkey | 234.4 (181.5 to 301.2) | -0.6 (-11.9 to 12.2) | 26.0 (19.2 to 31.8) | -36.9 (-55.1 to -8.7) | 387.5 (306.1 to 470.2) | -41.1 (-58.5 to -11.0) |
| United Arab Emirates | 247.1 (189.3 to 324.3) | 27.6 (13.7 to 42.1) | 33.0 (24.6 to 41.1) | -13.0 (-41.8 to 21.2) | 545.4 (424.6 to 681.0) | -21.5 (-48.3 to 7.9) |
| Yemen | 253.8 (200.1 to 325.2) | 0.9 (-9.4 to 14.2) | 66.3 (40.4 to 108.1) | -17.9 (-43.3 to 32.0) | 1207.5 (705.7 to 2037.5) | -21.4 (-47.1 to 29.8) |

ASR: Age-standardized rate; UI: Uncertainty interval; DALYs: Disability-adjusted life years

| **Table S9. Rheumatic heart disease burden by country.** | | | | | | | | |
| --- | --- | --- | --- | --- | --- | --- | --- | --- |
|  | **Prevalence (95%UI)** | | **Incidence (95%UI)** | | **Death (95%UI)** | | **DALYs (95%UI)** | |
| **Location** | **ASR (per 100,000) 2021** | **Percent change, 1990-2021** | **ASR (per 100,000) 2021** | **Percent change, 1990-2021** | **ASR (per 100,000) 2021** | **Percent change, 1990-2021** | **ASR (per 100,000) 2021** | **Percent change, 1990-2021** |
| **Central Asia** | **871.3 (685.9 to 1081.7)** | **4.3 (1.2 to 7.7)** | **56.7 (44.8 to 71.2)** | **0.6 (-2.7 to 4.2)** | **3.2 (2.8 to 3.7)** | **-46.9 (-57.5 to -35.8)** | **142.1 (120.7 to 168.0)** | **-45.9 (-54.4 to -37.5)** |
| Kazakhstan | 108.0 (92.1 to 126.2) | -40.7 (-48.8 to -32.5) | 7.2 (6.4 to 8.1) | -52.1 (-56.6 to -47.7) | 1.9 (1.5 to 2.3) | -75.9 (-82.8 to -68.8) | 58.1 (44.4 to 70.9) | -80.4 (-85.8 to -74.9) |
| Kyrgyzstan | 1004.0 (789.4 to 1241.1) | -2.1 (-7.8 to 4.4) | 66.4 (51.8 to 83.2) | -0.8 (-6.9 to 6.1) | 2.9 (2.3 to 3.6) | -62.1 (-71.4 to -50.0) | 143.0 (117.2 to 175.2) | -57.9 (-66.2 to -48.2) |
| Tajikistan | 1061.5 (832.7 to 1343.7) | -0.4 (-7.1 to 6.1) | 70.5 (54.5 to 90.1) | 1.3 (-6.7 to 8.9) | 1.8 (1.4 to 2.5) | -49.0 (-69.1 to -13.4) | 116.6 (88.7 to 154.8) | -36.2 (-52.3 to -10.2) |
| Turkmenistan | 1023.3 (793.0 to 1285.8) | -1.3 (-8.1 to 5.0) | 65.8 (51.3 to 83.6) | -3.9 (-11.1 to 2.7) | 2.9 (2.2 to 3.9) | -58.8 (-70.9 to -42.5) | 157.1 (125.3 to 197.0) | -50.2 (-60.4 to -37.8) |
| Uzbekistan | 1060.6 (821.5 to 1328.7) | 6.0 (-0.6 to 12.8) | 69.6 (54.0 to 87.9) | 3.3 (-2.8 to 10.7) | 4.8 (3.9 to 5.7) | -5.0 (-36.9 to 34.5) | 206.7 (168.7 to 246.6) | -16.0 (-36.9 to 7.0) |
| **East Asia** | **614.5 (488.5 to 756.6)** | **-12.3 (-14.9 to -9.9)** | **39.6 (31.4 to 49.3)** | **-18.0 (-20.1 to -16.0)** | **4.2 (3.3 to 5.3)** | **-77.7 (-84.0 to -68.0)** | **110.8 (90.2 to 135.1)** | **-76.2 (-82.0 to -67.5)** |
| China | 619.8 (492.1 to 763.7) | -12.5 (-15.1 to -10.0) | 39.9 (31.5 to 49.6) | -18.5 (-20.7 to -16.5) | 4.2 (3.3 to 5.4) | -77.9 (-84.3 to -68.1) | 111.7 (90.4 to 136.8) | -76.6 (-82.3 to -67.7) |
| Japan | 35.7 (31.2 to 40.6) | -32.8 (-37.6 to -28.1) | 2.7 (2.4 to 3.1) | -37.1 (-41.1 to -32.8) | 0.8 (0.6 to 0.9) | -65.1 (-69.3 to -27.8) | 13.9 (12.0 to 15.2) | -70.0 (-72.3 to -68.0) |
| Mongolia | 1061.5 (819.9 to 1331.9) | -1.1 (-7.4 to 6.0) | 71.3 (55.8 to 89.7) | -4.2 (-10.4 to 3.9) | 3.5 (2.9 to 4.1) | -60.9 (-75.0 to -37.4) | 159.1 (128.9 to 196.6) | -54.0 (-68.9 to -32.8) |
| North Korea | 710.7 (553.2 to 879.0) | -6.7 (-11.9 to -0.3) | 48.6 (38.2 to 60.7) | -4.2 (-10.0 to 1.9) | 5.9 (3.4 to 11.2) | -51.0 (-70.7 to -18.6) | 169.1 (108.6 to 289.8) | -46.9 (-64.7 to -17.3) |
| South Korea | 25.6 (20.7 to 30.4) | -22.1 (-31.2 to -12.4) | 1.6 (1.3 to 1.8) | -31.1 (-37.6 to -25.0) | 0.5 (0.4 to 0.6) | -67.1 (-74.1 to -58.8) | 9.4 (7.9 to 10.8) | -71.4 (-76.8 to -65.2) |
| Taiwan [Province of China] | 140.3 (120.3 to 162.3) | -27.6 (-37.2 to -16.8) | 8.4 (7.3 to 9.6) | -38.2 (-46.5 to -29.9) | 0.6 (0.5 to 0.7) | -85.7 (-87.7 to -84.1) | 19.0 (16.0 to 22.3) | -80.9 (-83.6 to -78.2) |
| **Southeast Asia** | **409.9 (328.5 to 501.0)** | **1.6 (-1.5 to 4.3)** | **28.2 (22.8 to 34.7)** | **-1.8 (-5.1 to 1.0)** | **1.1 (0.9 to 1.4)** | **-65.4 (-73.6 to -52.3)** | **65.9 (53.4 to 82.0)** | **-58.5 (-66.8 to -46.1)** |
| Brunei Darussalam | 70.1 (58.3 to 83.2) | -23.7 (-28.9 to -17.8) | 5.5 (4.8 to 6.3) | -25.0 (-29.3 to -20.5) | 2.1 (1.7 to 2.5) | -47.1 (-62.7 to -27.8) | 44.1 (37.6 to 51.4) | -51.5 (-64.6 to -34.2) |
| Cambodia | 681.9 (534.7 to 853.7) | -4.6 (-11.0 to 2.4) | 48.8 (38.5 to 61.0) | -6.4 (-12.6 to 0.5) | 1.6 (1.0 to 2.1) | -68.1 (-78.9 to -50.8) | 94.9 (68.0 to 127.0) | -62.0 (-73.6 to -44.8) |
| Indonesia | 206.7 (171.8 to 248.4) | 11.4 (7.6 to 15.2) | 15.4 (12.9 to 18.6) | -1.8 (-6.5 to 2.5) | 1.3 (0.9 to 1.9) | -63.2 (-73.0 to -47.3) | 62.5 (47.4 to 86.3) | -62.6 (-71.3 to -48.2) |
| Lao PDR | 889.2 (687.1 to 1114.0) | -3.6 (-11.0 to 3.6) | 62.1 (48.4 to 78.2) | -6.5 (-13.0 to 1.1) | 2.3 (1.4 to 3.8) | -69.2 (-80.3 to -52.1) | 147.9 (104.0 to 220.2) | -62.5 (-74.6 to -43.5) |
| Malaysia | 822.5 (654.3 to 1013.6) | 1.4 (-4.4 to 7.5) | 47.3 (37.2 to 59.7) | -2.1 (-8.0 to 3.9) | 0.8 (0.6 to 1.0) | -72.1 (-80.6 to -64.7) | 69.9 (54.3 to 90.0) | -55.2 (-64.4 to -46.1) |
| Myanmar | 861.6 (671.4 to 1073.3) | -4.6 (-14.7 to 5.5) | 60.1 (47.1 to 75.9) | -8.4 (-19.0 to 0.1) | 1.9 (1.4 to 2.6) | -72.8 (-81.1 to -58.6) | 125.3 (95.5 to 165.6) | -66.5 (-76.2 to -51.5) |
| Philippines | 626.9 (486.6 to 780.1) | 5.4 (1.9 to 9.3) | 42.3 (33.2 to 52.9) | 4.0 (0.1 to 7.9) | 1.0 (0.7 to 1.5) | -53.7 (-67.1 to -39.6) | 77.5 (55.7 to 104.4) | -40.8 (-51.2 to -28.7) |
| Singapore | 25.2 (20.9 to 29.8) | -55.8 (-61.8 to -49.1) | 1.4 (1.2 to 1.6) | -63.2 (-68.0 to -58.5) | 0.6 (0.5 to 0.6) | -76.5 (-79.5 to -73.8) | 11.5 (10.3 to 12.7) | -79.5 (-81.7 to -77.5) |
| Thailand | 723.7 (568.9 to 892.5) | -2.5 (-8.4 to 4.0) | 45.6 (35.7 to 57.8) | -5.9 (-12.1 to 0.1) | 0.4 (0.3 to 0.5) | -54.7 (-73.0 to -17.9) | 50.6 (37.2 to 70.2) | -31.4 (-49.1 to -10.3) |
| Timor-Leste | 787.4 (614.5 to 993.2) | -0.7 (-7.3 to 6.3) | 52.8 (40.9 to 66.5) | -2.9 (-9.5 to 4.8) | 2.3 (1.3 to 4.2) | -52.9 (-68.9 to -26.7) | 139.3 (87.6 to 232.6) | -46.8 (-63.5 to -23.6) |
| Viet Nam | 124.3 (106.0 to 145.4) | 16.3 (7.4 to 26.0) | 8.6 (7.5 to 9.8) | -1.8 (-8.5 to 5.0) | 0.9 (0.6 to 1.2) | -65.7 (-78.3 to -44.9) | 37.4 (27.7 to 48.9) | -63.5 (-75.4 to -45.6) |
| **South Asia** | **732.2 (575.8 to 916.5)** | **3.4 (0.8 to 5.8)** | **51.3 (40.4 to 64.3)** | **2.1 (-0.5 to 4.5)** | **14.9 (12.2 to 20.0)** | **-46.5 (-57.4 to -33.9)** | **453.6 (380.1 to 580.9)** | **-48.8 (-58.4 to -38.1)** |
| Afghanistan | 698.2 (550.9 to 858.6) | 1.2 (-4.5 to 7.9) | 52.8 (41.8 to 65.6) | 1.1 (-4.9 to 8.2) | 4.2 (2.5 to 6.6) | -52.6 (-66.1 to -34.1) | 174.8 (117.0 to 247.1) | -52.1 (-64.5 to -35.3) |
| Bangladesh | 755.6 (588.8 to 937.0) | 6.4 (-3.2 to 16.6) | 51.5 (39.5 to 65.2) | 3.3 (-6.5 to 13.6) | 16.3 (11.1 to 27.9) | -45.5 (-64.3 to -22.8) | 457.6 (325.0 to 745.6) | -50.2 (-64.1 to -32.1) |
| Bhutan | 733.2 (576.9 to 916.4) | 1.7 (-6.0 to 9.6) | 47.9 (37.6 to 60.3) | -0.9 (-8.4 to 7.7) | 14.3 (8.0 to 27.4) | -53.5 (-72.3 to -32.5) | 401.9 (251.2 to 725.1) | -57.3 (-72.9 to -38.6) |
| India | 690.9 (539.6 to 867.3) | 0.3 (-2.5 to 2.7) | 48.3 (37.8 to 61.1) | -1.4 (-4.3 to 1.2) | 14.3 (11.8 to 19.0) | -48.0 (-60.1 to -34.3) | 431.5 (361.6 to 548.6) | -51.3 (-60.9 to -39.8) |
| Iran | 709.9 (558.3 to 877.4) | 2.0 (0.3 to 3.9) | 44.4 (34.9 to 55.7) | -0.3 (-1.8 to 1.4) | 1.4 (1.1 to 1.6) | -58.8 (-73.0 to -39.8) | 69.8 (55.7 to 88.5) | -51.1 (-64.2 to -35.8) |
| Maldives | 764.8 (590.9 to 948.7) | -2.8 (-9.1 to 3.1) | 45.3 (35.8 to 57.5) | -5.9 (-12.0 to 0.1) | 0.5 (0.4 to 0.6) | -85.4 (-89.9 to -75.8) | 55.9 (41.3 to 76.6) | -71.3 (-79.8 to -57.5) |
| Nepal | 692.5 (543.5 to 875.1) | 4.0 (-4.7 to 13.8) | 48.4 (37.9 to 61.4) | 2.5 (-7.6 to 13.9) | 17.0 (11.5 to 26.8) | -47.4 (-64.9 to -23.7) | 489.9 (343.9 to 726.5) | -51.5 (-67.3 to -32.1) |
| Pakistan | 991.0 (789.8 to 1236.0) | 12.2 (7.5 to 17.1) | 68.5 (54.0 to 86.5) | 11.6 (6.9 to 16.7) | 17.9 (13.1 to 25.8) | -32.8 (-51.9 to -12.3) | 585.0 (443.3 to 816.9) | -28.5 (-47.5 to -7.8) |
| Sri Lanka | 62.9 (51.8 to 75.7) | -11.1 (-19.9 to -3.0) | 4.6 (3.9 to 5.3) | -24.2 (-29.9 to -17.9) | 0.5 (0.4 to 0.7) | -72.6 (-82.9 to -56.9) | 25.0 (17.9 to 33.6) | -71.4 (-81.2 to -57.1) |
| Armenia | 1114.3 (879.7 to 1368.2) | -4.8 (-10.7 to 0.7) | 64.2 (49.8 to 79.2) | -6.5 (-12.8 to -0.3) | 1.9 (1.4 to 2.3) | -59.8 (-76.5 to -42.2) | 104.2 (77.0 to 135.0) | -51.1 (-65.2 to -38.9) |
| Azerbaijan | 1029.5 (811.0 to 1288.0) | -3.2 (-9.0 to 2.4) | 63.1 (49.4 to 80.2) | -4.6 (-10.2 to 1.9) | 1.4 (0.8 to 2.0) | -50.8 (-75.2 to -21.6) | 93.8 (68.4 to 125.1) | -36.8 (-55.2 to -19.2) |
| Bahrain | 59.9 (49.7 to 70.9) | -18.0 (-24.9 to -11.0) | 4.4 (3.7 to 5.1) | -22.7 (-28.5 to -17.1) | 0.8 (0.6 to 0.9) | -78.6 (-85.9 to -71.3) | 20.6 (16.5 to 25.4) | -79.3 (-85.8 to -72.2) |
| Cyprus | 34.7 (28.3 to 42.4) | -40.1 (-45.6 to -34.1) | 4.9 (4.4 to 5.5) | -46.6 (-50.2 to -43.3) | 2.7 (2.1 to 3.1) | -67.6 (-78.0 to -53.6) | 44.1 (36.7 to 51.4) | -68.6 (-79.3 to -55.3) |
| Georgia | 1078.7 (847.5 to 1331.1) | -2.7 (-7.8 to 2.8) | 65.5 (51.2 to 82.0) | -1.9 (-8.1 to 4.5) | 4.3 (3.7 to 5.1) | -36.1 (-49.5 to -13.7) | 169.3 (142.9 to 201.8) | -44.7 (-54.1 to -30.5) |
| Iraq | 741.8 (575.0 to 921.0) | 2.1 (-4.6 to 9.4) | 47.9 (37.7 to 59.5) | 0.4 (-6.6 to 8.4) | 1.9 (1.4 to 2.4) | -55.7 (-73.7 to -26.3) | 88.4 (69.3 to 110.6) | -51.3 (-66.9 to -29.9) |
| Israel | 39.1 (32.3 to 47.5) | -23.1 (-31.3 to -14.0) | 3.8 (3.3 to 4.3) | -24.1 (-29.6 to -18.5) | 1.5 (1.3 to 1.6) | -45.9 (-52.1 to -39.7) | 29.3 (26.6 to 31.9) | -52.0 (-56.8 to -47.3) |
| Jordan | 41.4 (34.6 to 48.9) | -19.1 (-25.2 to -12.8) | 2.7 (2.3 to 3.2) | -27.6 (-32.7 to -22.6) | 0.5 (0.4 to 0.6) | -62.7 (-77.8 to -41.3) | 16.7 (13.2 to 20.6) | -64.2 (-77.2 to -47.3) |
| Kuwait | 50.5 (42.2 to 58.8) | -23.0 (-30.3 to -14.6) | 3.2 (2.7 to 3.7) | -29.9 (-36.1 to -23.5) | 0.4 (0.3 to 0.5) | -82.3 (-86.4 to -78.5) | 15.3 (12.4 to 18.4) | -82.2 (-85.4 to -78.7) |
| Lebanon | 49.6 (41.3 to 58.1) | -16.3 (-22.3 to -10.6) | 3.5 (2.9 to 4.1) | -22.7 (-27.5 to -17.8) | 1.1 (0.9 to 1.3) | -79.2 (-86.6 to -67.4) | 32.6 (27.6 to 39.0) | -79.1 (-86.1 to -68.5) |
| Oman | 58.1 (47.9 to 69.7) | 7.9 (0.6 to 16.1) | 4.4 (3.7 to 5.1) | 8.4 (1.5 to 16.6) | 1.1 (0.6 to 1.4) | -70.3 (-82.7 to -50.8) | 30.9 (20.3 to 39.8) | -73.6 (-83.9 to -56.3) |
| Palestine | 734.7 (582.3 to 903.2) | -0.2 (-6.5 to 6.4) | 45.0 (35.0 to 56.3) | -1.7 (-8.6 to 6.6) | 1.3 (1.1 to 1.5) | -61.6 (-76.7 to -35.7) | 71.2 (56.4 to 91.1) | -50.7 (-65.9 to -30.4) |
| Qatar | 65.9 (54.8 to 78.4) | -23.9 (-30.1 to -17.6) | 5.1 (4.4 to 5.9) | -20.4 (-26.7 to -14.3) | 0.9 (0.7 to 1.1) | -72.4 (-84.7 to -58.7) | 24.9 (19.5 to 31.4) | -72.1 (-82.7 to -59.9) |
| Saudi Arabia | 57.1 (47.3 to 67.6) | -22.1 (-27.9 to -16.4) | 3.9 (3.3 to 4.6) | -26.2 (-31.1 to -20.8) | 0.7 (0.5 to 1.0) | -71.7 (-84.5 to -50.0) | 26.1 (18.5 to 34.3) | -72.8 (-84.9 to -52.8) |
| Syrian Arab Republic | 718.3 (564.8 to 887.8) | 1.4 (-5.7 to 7.7) | 46.1 (36.2 to 57.1) | 0.5 (-6.5 to 7.0) | 1.9 (1.4 to 2.7) | -65.5 (-81.4 to -40.0) | 91.7 (67.6 to 121.3) | -63.0 (-77.1 to -41.8) |
| Turkey | 49.5 (40.7 to 58.5) | -11.7 (-18.7 to -2.9) | 3.2 (2.7 to 3.8) | -16.2 (-23.6 to -7.3) | 0.9 (0.7 to 1.1) | -55.3 (-74.4 to -30.6) | 25.4 (21.1 to 30.2) | -62.3 (-76.0 to -44.2) |
| United Arab Emirates | 699.4 (543.2 to 875.7) | -2.3 (-7.4 to 4.3) | 40.2 (31.2 to 50.7) | 0.7 (-4.9 to 7.4) | 1.9 (1.1 to 2.6) | -58.7 (-80.3 to -34.9) | 81.3 (60.5 to 106.0) | -53.7 (-70.3 to -34.2) |
| Yemen | 850.3 (668.1 to 1062.8) | 5.5 (-4.1 to 13.9) | 60.2 (46.9 to 76.0) | 5.5 (-4.3 to 14.2) | 3.2 (2.1 to 5.0) | -53.8 (-70.9 to -27.2) | 138.7 (99.5 to 194.8) | -53.2 (-68.7 to -31.4) |

ASR: Age-standardized rate; UI: Uncertainty interval; DALYs: Disability-adjusted life years

| **Table S10. Non-rheumatic valvular heart disease burden by country.** | | | | | | | | |
| --- | --- | --- | --- | --- | --- | --- | --- | --- |
|  | **Prevalence (95%UI)** | | **Incidence (95%UI)** | | **Death (95%UI)** | | **DALYs (95%UI)** | |
| **Location** | **ASR (per 100,000) 2021** | **Percent change, 1990-2021** | **ASR (per 100,000) 2021** | **Percent change, 1990-2021** | **ASR (per 100,000) 2021** | **Percent change, 1990-2021** | **ASR (per 100,000) 2021** | **Percent change, 1990-2021** |
| **Central Asia** | **637.6 (555.6 to 731.1)** | **39.6 (28.0 to 52.4)** | **46.9 (40.7 to 53.9)** | **37.3 (24.5 to 51.8)** | **0.7 (0.6 to 0.8)** | **305.2 (231.7 to 399.1)** | **23.9 (19.4 to 30.0)** | **113.4 (84.1 to 159.1)** |
| Kazakhstan | 550.4 (468.2 to 649.8) | 23.1 (13.0 to 35.6) | 41.4 (34.8 to 49.0) | 22.4 (10.6 to 37.0) | 0.6 (0.5 to 0.7) | 248.4 (164.8 to 364.8) | 20.6 (16.3 to 26.1) | 88.5 (57.5 to 139.3) |
| Kyrgyzstan | 475.5 (399.9 to 563.1) | 7.2 (-2.9 to 18.2) | 35.2 (29.7 to 41.7) | 4.6 (-6.2 to 17.1) | 0.3 (0.2 to 0.3) | 153.3 (98.0 to 221.1) | 13.7 (10.2 to 18.8) | 40.3 (20.6 to 70.8) |
| Tajikistan | 472.2 (394.2 to 572.2) | 8.4 (-2.1 to 20.4) | 33.7 (27.9 to 40.9) | 4.8 (-6.3 to 17.5) | 0.0 (0.0 to 0.0) | -11.6 (-44.6 to 39.2) | 7.8 (4.5 to 13.1) | 6.3 (-6.9 to 22.1) |
| Turkmenistan | 536.5 (455.0 to 642.8) | 19.1 (10.1 to 28.5) | 39.5 (33.2 to 47.5) | 17.3 (7.1 to 28.8) | 0.3 (0.2 to 0.4) | 550.6 (384.8 to 800.6) | 15.4 (11.3 to 21.7) | 92.1 (54.8 to 160.1) |
| Uzbekistan | 647.5 (550.8 to 775.3) | 50.8 (30.0 to 77.6) | 48.7 (41.4 to 58.7) | 53.5 (28.8 to 83.4) | 0.6 (0.5 to 0.7) | 199.9 (99.5 to 350.0) | 24.0 (18.7 to 31.2) | 100.3 (61.8 to 152.2) |
| **East Asia** | **157.1 (148.3 to 166.9)** | **21.8 (18.1 to 26.4)** | **12.8 (12.1 to 13.7)** | **23.6 (19.9 to 28.0)** | **0.2 (0.1 to 0.2)** | **-40.9 (-54.2 to -20.4)** | **5.4 (4.3 to 7.0)** | **-32.4 (-46.1 to -15.2)** |
| China | 156.2 (147.7 to 165.6) | 22.2 (18.7 to 26.8) | 12.7 (12.0 to 13.5) | 24.1 (20.5 to 28.3) | 0.1 (0.1 to 0.2) | -42.0 (-57.7 to -15.4) | 4.9 (3.8 to 6.5) | -31.2 (-46.6 to -10.9) |
| Japan | 709.4 (661.1 to 763.9) | 9.1 (5.4 to 13.0) | 56.3 (52.0 to 61.1) | 11.6 (7.7 to 15.7) | 3.3 (2.5 to 3.7) | -43.0 (-50.8 to -38.7) | 49.3 (41.2 to 56.3) | -50.5 (-55.2 to -46.8) |
| Mongolia | 704.0 (588.8 to 832.4) | 15.6 (0.3 to 35.3) | 51.3 (42.5 to 61.7) | 12.2 (-4.2 to 32.6) | 0.8 (0.6 to 1.0) | 22.9 (-22.6 to 91.4) | 28.9 (22.5 to 38.0) | 17.3 (-12.0 to 52.5) |
| North Korea | 108.2 (89.0 to 130.4) | -3.2 (-12.5 to 7.8) | 9.0 (7.4 to 10.9) | -2.3 (-12.6 to 9.5) | 0.2 (0.1 to 0.5) | -29.2 (-50.5 to 1.0) | 6.5 (4.3 to 13.1) | -24.3 (-43.0 to 1.6) |
| South Korea | 492.3 (430.7 to 558.3) | 20.8 (9.0 to 33.0) | 36.2 (31.6 to 41.5) | 18.0 (5.6 to 31.2) | 1.5 (1.0 to 1.9) | -13.7 (-50.8 to 30.0) | 24.6 (18.5 to 30.3) | -25.2 (-50.4 to 2.4) |
| Taiwan [Province of China] | 227.1 (194.1 to 268.2) | 14.4 (-2.5 to 33.3) | 18.8 (15.9 to 22.1) | 15.6 (-2.5 to 36.1) | 1.2 (1.1 to 1.3) | -45.9 (-51.2 to -41.0) | 25.1 (22.6 to 27.7) | -47.3 (-52.0 to -43.3) |
| **Southeast Asia** | **109.2 (97.5 to 123.8)** | **23.1 (17.9 to 28.4)** | **8.7 (7.8 to 9.8)** | **22.7 (17.9 to 28.1)** | **0.4 (0.3 to 0.6)** | **22.0 (-4.8 to 60.3)** | **8.5 (6.7 to 13.0)** | **13.0 (-7.4 to 40.9)** |
| Brunei Darussalam | 652.5 (570.1 to 743.5) | 16.0 (4.2 to 28.5) | 49.7 (43.6 to 56.8) | 18.1 (5.4 to 31.6) | 3.7 (2.6 to 4.6) | -1.1 (-24.2 to 30.3) | 65.8 (49.3 to 78.0) | -5.7 (-23.5 to 18.9) |
| Cambodia | 89.7 (74.7 to 108.5) | 16.1 (5.1 to 28.5) | 7.2 (6.0 to 8.7) | 16.0 (4.5 to 29.0) | 0.2 (0.1 to 0.5) | -7.5 (-34.5 to 24.7) | 4.8 (3.1 to 10.8) | -9.2 (-30.8 to 17.6) |
| Indonesia | 103.1 (95.6 to 111.9) | 20.5 (17.4 to 23.5) | 8.3 (7.7 to 9.0) | 21.6 (18.3 to 24.9) | 0.2 (0.1 to 0.7) | 12.1 (-16.8 to 44.8) | 6.1 (3.9 to 13.5) | 4.2 (-16.3 to 29.8) |
| Lao PDR | 99.1 (81.6 to 118.3) | 20.0 (8.2 to 32.8) | 7.9 (6.5 to 9.4) | 18.2 (5.8 to 31.7) | 0.2 (0.1 to 0.6) | -19.9 (-47.5 to 14.6) | 5.7 (3.6 to 13.0) | -16.5 (-40.3 to 17.1) |
| Malaysia | 134.3 (114.8 to 161.8) | 28.0 (14.9 to 41.5) | 10.6 (9.0 to 12.8) | 27.7 (14.2 to 42.9) | 0.6 (0.5 to 0.8) | -8.0 (-27.9 to 25.3) | 14.4 (12.3 to 17.4) | -10.8 (-27.2 to 16.0) |
| Myanmar | 97.7 (80.9 to 118.4) | 21.4 (11.3 to 34.7) | 7.9 (6.6 to 9.5) | 20.9 (9.4 to 34.4) | 0.2 (0.1 to 0.6) | -16.9 (-44.0 to 16.0) | 5.9 (4.0 to 12.9) | -15.7 (-39.2 to 13.8) |
| Philippines | 88.2 (81.7 to 95.9) | 6.7 (4.5 to 9.4) | 7.1 (6.6 to 7.7) | 9.3 (6.9 to 12.2) | 0.2 (0.2 to 0.3) | 12.8 (-6.8 to 32.6) | 6.0 (4.6 to 7.5) | 19.0 (2.7 to 36.7) |
| Singapore | 506.1 (434.4 to 582.0) | -2.7 (-11.2 to 5.9) | 36.0 (30.7 to 41.8) | -4.6 (-13.4 to 5.0) | 0.8 (0.7 to 0.9) | -57.3 (-61.6 to -53.6) | 17.9 (15.2 to 21.9) | -56.9 (-60.8 to -52.5) |
| Thailand | 124.7 (104.3 to 148.6) | 27.0 (13.5 to 43.5) | 10.0 (8.4 to 11.8) | 27.1 (13.0 to 44.9) | 0.7 (0.5 to 1.0) | 78.9 (-0.6 to 203.5) | 17.6 (13.0 to 23.2) | 75.3 (10.3 to 171.8) |
| Timor-Leste | 96.9 (80.5 to 117.5) | 14.0 (3.9 to 27.9) | 7.6 (6.3 to 9.2) | 13.6 (2.7 to 29.3) | 0.2 (0.1 to 0.5) | -3.0 (-32.8 to 45.7) | 5.2 (3.3 to 11.6) | 0.0 (-25.0 to 32.4) |
| Viet Nam | 101.7 (84.8 to 122.2) | 22.9 (12.6 to 35.1) | 8.2 (6.9 to 9.9) | 22.1 (11.2 to 35.3) | 0.2 (0.1 to 0.6) | 3.2 (-26.3 to 35.8) | 5.4 (3.5 to 11.7) | 2.2 (-19.5 to 31.6) |
| **South Asia** | **60.5 (54.9 to 66.9)** | **16.4 (13.6 to 19.7)** | **5.4 (4.9 to 5.9)** | **12.7 (9.5 to 15.6)** | **1.3 (1.0 to 1.5)** | **20.2 (-3.2 to 56.5)** | **26.9 (21.9 to 31.7)** | **13.7 (-10.0 to 45.9)** |
| Afghanistan | 78.8 (67.4 to 91.6) | -3.1 (-10.6 to 5.5) | 6.9 (6.1 to 7.9) | -4.5 (-11.5 to 3.1) | 1.9 (1.1 to 2.8) | -5.0 (-29.1 to 36.6) | 51.7 (26.9 to 83.2) | -7.1 (-32.0 to 37.0) |
| Bangladesh | 61.5 (52.4 to 72.1) | 17.2 (7.8 to 28.7) | 5.5 (4.8 to 6.2) | 14.7 (5.8 to 24.7) | 1.4 (1.0 to 2.0) | 16.3 (-22.9 to 69.3) | 26.8 (18.5 to 37.2) | 3.4 (-31.9 to 56.2) |
| Bhutan | 70.5 (60.3 to 82.5) | 32.0 (20.4 to 44.4) | 6.1 (5.4 to 7.0) | 29.3 (19.0 to 40.5) | 1.3 (1.0 to 1.7) | 27.4 (-13.8 to 96.0) | 25.5 (18.9 to 34.8) | 15.9 (-22.5 to 80.9) |
| India | 59.9 (54.5 to 66.2) | 16.6 (13.8 to 19.9) | 5.3 (4.9 to 5.8) | 12.2 (9.3 to 15.3) | 1.3 (1.0 to 1.5) | 20.0 (-5.0 to 61.0) | 26.4 (21.7 to 31.3) | 12.9 (-11.3 to 48.8) |
| Iran | 112.4 (101.8 to 124.1) | 16.1 (12.7 to 19.9) | 9.0 (8.2 to 9.9) | 13.4 (9.8 to 17.1) | 1.2 (1.0 to 1.5) | -21.0 (-37.3 to -4.1) | 30.6 (26.7 to 36.9) | -24.2 (-40.8 to -5.9) |
| Maldives | 157.5 (134.9 to 185.4) | 35.8 (20.1 to 53.1) | 12.9 (11.1 to 15.1) | 41.3 (25.1 to 59.7) | 0.8 (0.6 to 1.0) | 1.2 (-35.3 to 63.0) | 16.4 (12.7 to 20.7) | -15.3 (-46.7 to 44.1) |
| Nepal | 63.2 (53.8 to 74.0) | 26.4 (16.2 to 38.8) | 5.7 (5.0 to 6.5) | 25.0 (15.9 to 36.0) | 1.3 (0.9 to 1.8) | 27.9 (-7.2 to 87.2) | 25.8 (18.6 to 34.7) | 18.1 (-17.0 to 69.7) |
| Pakistan | 65.3 (59.1 to 72.5) | 16.1 (12.5 to 19.9) | 6.0 (5.5 to 6.5) | 15.4 (11.6 to 19.0) | 1.5 (1.1 to 2.0) | 35.2 (4.8 to 77.6) | 30.9 (23.2 to 42.9) | 34.9 (3.8 to 78.0) |
| Sri Lanka | 166.0 (141.1 to 196.2) | 25.6 (11.4 to 42.6) | 13.7 (11.7 to 16.2) | 27.1 (11.7 to 45.1) | 0.5 (0.3 to 0.7) | -48.6 (-67.3 to -12.0) | 12.3 (8.6 to 16.9) | -43.1 (-61.6 to -13.1) |
| Armenia | 546.6 (475.2 to 625.7) | 36.2 (23.1 to 53.5) | 40.7 (34.9 to 47.3) | 37.7 (22.9 to 57.1) | 0.6 (0.5 to 0.7) | 133.5 (80.0 to 204.8) | 19.0 (15.1 to 24.5) | 60.9 (36.8 to 92.5) |
| Azerbaijan | 563.6 (483.6 to 658.0) | 25.2 (14.7 to 38.7) | 41.8 (35.6 to 48.9) | 21.8 (9.4 to 37.2) | 0.1 (0.1 to 0.1) | -5.9 (-40.2 to 44.8) | 10.5 (6.8 to 16.5) | 12.4 (-4.7 to 31.9) |
| Bahrain | 138.3 (117.9 to 160.0) | 17.4 (7.9 to 29.1) | 11.5 (10.0 to 13.0) | 17.0 (7.8 to 28.0) | 1.6 (1.2 to 2.0) | -24.7 (-42.2 to -1.4) | 32.3 (25.1 to 40.6) | -29.7 (-45.8 to -7.8) |
| Cyprus | 469.1 (417.4 to 538.3) | 27.1 (9.7 to 45.6) | 45.9 (41.7 to 51.0) | 17.7 (3.7 to 33.9) | 9.3 (6.9 to 11.7) | -52.4 (-65.5 to -32.5) | 124.5 (99.9 to 153.8) | -52.4 (-63.9 to -35.6) |
| Georgia | 1206.0 (1048.2 to 1383.9) | 123.3 (88.7 to 162.8) | 94.6 (80.7 to 109.3) | 133.0 (94.2 to 179.0) | 2.8 (2.4 to 3.2) | 1477.2 (1164.4 to 1848.8) | 79.1 (67.7 to 93.8) | 537.7 (388.2 to 746.1) |
| Iraq | 87.2 (73.7 to 102.5) | 3.0 (-5.0 to 12.5) | 6.7 (5.8 to 7.8) | 0.7 (-7.5 to 10.0) | 1.2 (0.9 to 1.5) | -0.4 (-30.1 to 40.5) | 31.1 (22.4 to 40.4) | -11.0 (-39.0 to 31.1) |
| Israel | 377.8 (333.3 to 428.3) | 12.8 (0.7 to 24.6) | 34.2 (30.3 to 38.2) | 13.8 (2.3 to 25.4) | 4.6 (3.8 to 5.1) | -17.8 (-26.6 to -9.1) | 69.4 (60.8 to 76.1) | -25.5 (-32.3 to -18.3) |
| Jordan | 99.3 (84.8 to 116.3) | 10.5 (3.0 to 19.4) | 7.5 (6.5 to 8.6) | 6.2 (-1.4 to 14.4) | 0.7 (0.5 to 0.9) | -41.9 (-57.0 to -21.0) | 17.0 (13.6 to 21.5) | -45.2 (-59.4 to -25.6) |
| Kuwait | 143.6 (123.5 to 164.8) | 21.1 (11.4 to 30.9) | 11.2 (9.8 to 12.6) | 22.4 (13.3 to 33.1) | 0.8 (0.7 to 1.0) | -18.9 (-33.0 to -2.7) | 20.0 (17.1 to 23.8) | -29.2 (-40.9 to -15.8) |
| Lebanon | 124.5 (109.0 to 144.2) | 12.9 (5.5 to 21.5) | 9.7 (8.6 to 11.0) | 8.9 (2.1 to 17.3) | 2.0 (1.6 to 2.5) | -57.9 (-72.2 to -32.9) | 46.0 (37.2 to 56.4) | -60.8 (-74.8 to -34.5) |
| Oman | 116.2 (99.6 to 134.4) | 41.8 (27.5 to 57.3) | 9.3 (8.1 to 10.7) | 42.3 (28.2 to 59.0) | 0.6 (0.4 to 0.7) | -28.3 (-54.5 to 7.3) | 14.2 (11.2 to 17.3) | -32.8 (-56.2 to 1.9) |
| Palestine | 79.9 (68.1 to 93.6) | 3.3 (-2.6 to 9.6) | 6.2 (5.3 to 7.1) | 1.2 (-5.2 to 7.6) | 0.6 (0.5 to 0.8) | -25.3 (-46.5 to 1.6) | 14.7 (11.8 to 17.7) | -27.3 (-48.8 to 0.7) |
| Qatar | 160.7 (137.6 to 186.0) | -3.1 (-11.3 to 6.3) | 13.0 (11.3 to 15.0) | -7.2 (-14.9 to 1.4) | 1.3 (1.0 to 1.7) | -50.1 (-65.4 to -32.3) | 27.7 (20.7 to 36.0) | -50.0 (-64.7 to -32.2) |
| Saudi Arabia | 141.9 (123.5 to 163.1) | 32.3 (21.0 to 45.2) | 11.2 (9.8 to 12.7) | 28.2 (17.2 to 41.0) | 1.1 (0.9 to 1.5) | -5.9 (-34.7 to 40.7) | 31.7 (24.1 to 41.0) | -3.8 (-34.8 to 46.8) |
| Syrian Arab Republic | 164.9 (141.5 to 190.2) | 10.9 (0.3 to 23.1) | 13.8 (12.1 to 15.8) | 8.4 (-1.7 to 21.1) | 4.5 (3.3 to 6.1) | -18.6 (-43.3 to 16.9) | 105.3 (76.4 to 147.0) | -25.4 (-49.6 to 9.2) |
| Turkey | 141.4 (122.3 to 162.4) | 5.9 (-4.6 to 16.5) | 11.4 (10.1 to 12.8) | 3.4 (-6.2 to 13.4) | 2.1 (1.6 to 2.7) | -36.0 (-56.0 to -9.7) | 48.2 (38.1 to 60.8) | -42.4 (-59.9 to -19.0) |
| United Arab Emirates | 156.8 (134.9 to 182.5) | 8.9 (-0.5 to 19.7) | 11.9 (10.4 to 13.8) | 2.8 (-6.4 to 12.8) | 3.0 (2.3 to 3.8) | -17.8 (-42.4 to 13.3) | 61.6 (48.4 to 77.1) | -30.9 (-52.2 to -5.5) |
| Yemen | 83.1 (70.1 to 96.1) | 1.7 (-6.4 to 11.7) | 7.1 (6.2 to 8.1) | -0.1 (-7.4 to 9.6) | 1.9 (1.3 to 2.7) | -0.9 (-33.0 to 53.2) | 47.9 (32.3 to 70.6) | -4.5 (-36.1 to 55.4) |

ASR: Age-standardized rate; UI: Uncertainty interval; DALYs: Disability-adjusted life years

| **Table S11. Cardiomyopathy and myocarditis burden by country.** | | | | | | | | |
| --- | --- | --- | --- | --- | --- | --- | --- | --- |
|  | **Prevalence (95%UI)** | | **Incidence (95%UI)** | | **Death (95%UI)** | | **DALYs (95%UI)** | |
| **Location** | **ASR (per 100,000) 2021** | **Percent change, 1990-2021** | **ASR (per 100,000) 2021** | **Percent change, 1990-2021** | **ASR (per 100,000) 2021** | **Percent change, 1990-2021** | **ASR (per 100,000) 2021** | **Percent change, 1990-2021** |
| **Central Asia** | **69.2 (57.8 to 81.4)** | **84.8 (67.4 to 101.3)** | **16.5 (13.3 to 20.4)** | **1.3 (0.9 to 1.7)** | **13.9 (11.9 to 16.0)** | **180.1 (124.3 to 243.3)** | **413.5 (348.4 to 479.8)** | **163.9 (111.6 to 227.6)** |
| Kazakhstan | 146.5 (116.3 to 175.2) | 363.3 (268.6 to 467.8) | 16.4 (13.1 to 20.2) | 0.5 (0.3 to 0.8) | 41.8 (34.1 to 49.2) | 2050.3 (1562.4 to 2675.5) | 1202.9 (979.7 to 1442.3) | 1698.4 (1272.9 to 2219.3) |
| Kyrgyzstan | 65.8 (54.5 to 78.3) | 112.7 (95.3 to 130.5) | 16.4 (13.2 to 20.4) | 0.8 (0.5 to 1.1) | 9.9 (8.2 to 11.9) | 112.1 (63.6 to 173.0) | 338.9 (282.5 to 406.2) | 94.6 (53.1 to 147.7) |
| Tajikistan | 23.5 (19.2 to 28.2) | 29.0 (16.5 to 40.6) | 16.7 (13.4 to 20.8) | 1.4 (1.0 to 1.8) | 1.2 (0.9 to 1.6) | -27.0 (-52.0 to 7.0) | 40.4 (29.1 to 55.6) | -19.2 (-48.6 to 20.5) |
| Turkmenistan | 92.9 (77.8 to 110.1) | -0.5 (-9.2 to 8.8) | 16.7 (13.4 to 20.6) | 1.7 (1.3 to 2.0) | 15.8 (12.5 to 20.0) | -19.1 (-36.5 to 5.6) | 541.8 (434.9 to 685.7) | -12.5 (-31.0 to 15.7) |
| Uzbekistan | 24.6 (20.7 to 28.8) | 50.9 (35.2 to 65.3) | 16.6 (13.3 to 20.5) | 0.9 (0.5 to 1.3) | 2.3 (1.9 to 2.7) | 89.9 (45.3 to 159.0) | 71.7 (59.2 to 84.9) | 66.4 (26.8 to 122.7) |
| **East Asia** | **28.4 (23.2 to 34.2)** | **67.1 (53.6 to 80.1)** | **16.3 (13.2 to 19.9)** | **-6.1 (-8.9 to -3.3)** | **1.9 (1.5 to 2.3)** | **-4.1 (-47.5 to 32.0)** | **53.2 (43.8 to 63.8)** | **-30.5 (-54.9 to -8.9)** |
| China | 28.2 (23.0 to 34.0) | 72.0 (58.0 to 85.5) | 16.3 (13.3 to 20.0) | -6.3 (-9.0 to -3.5) | 1.9 (1.5 to 2.3) | -1.0 (-47.7 to 38.3) | 53.0 (43.2 to 63.9) | -30.4 (-55.5 to -7.9) |
| Japan | 102.4 (86.6 to 121.0) | -1.7 (-8.3 to 5.0) | 19.8 (16.4 to 24.2) | -3.7 (-6.2 to -1.1) | 2.2 (1.9 to 2.3) | -68.3 (-69.7 to -66.7) | 62.7 (58.7 to 67.7) | -62.6 (-64.3 to -60.6) |
| Mongolia | 92.9 (76.0 to 112.2) | 40.5 (25.6 to 55.1) | 16.4 (13.2 to 20.4) | -0.5 (-0.7 to -0.3) | 15.8 (11.9 to 20.6) | 2.6 (-32.3 to 61.5) | 463.4 (351.8 to 607.6) | 3.8 (-32.4 to 64.7) |
| North Korea | 24.2 (19.8 to 28.8) | 20.3 (11.5 to 29.7) | 14.7 (11.9 to 18.3) | 1.7 (1.3 to 2.0) | 2.7 (1.8 to 4.1) | -5.2 (-35.1 to 41.4) | 71.4 (50.7 to 102.2) | -13.8 (-40.8 to 23.3) |
| South Korea | 91.9 (76.7 to 108.8) | 70.3 (53.8 to 89.1) | 18.2 (14.9 to 22.2) | 1.6 (1.3 to 2.0) | 1.5 (1.2 to 1.8) | -45.1 (-59.7 to -17.9) | 44.7 (37.3 to 52.1) | -43.0 (-60.0 to -19.5) |
| Taiwan [Province of China] | 44.6 (36.0 to 53.1) | -2.5 (-11.7 to 7.6) | 14.0 (12.0 to 16.1) | -4.5 (-15.4 to 8.1) | 1.5 (1.4 to 1.7) | -49.9 (-54.0 to -45.5) | 51.7 (47.4 to 56.1) | -36.7 (-41.7 to -31.1) |
| **Southeast Asia** | **32.6 (27.1 to 38.8)** | **11.2 (3.9 to 18.2)** | **17.9 (14.5 to 21.9)** | **0.2 (-0.2 to 0.7)** | **3.4 (3.0 to 3.9)** | **-21.4 (-38.6 to 1.7)** | **81.9 (70.9 to 96.1)** | **-16.5 (-32.1 to 7.0)** |
| Brunei Darussalam | 86.6 (70.8 to 103.9) | 6.4 (-2.2 to 15.9) | 18.4 (15.0 to 22.4) | -0.6 (-0.8 to -0.3) | 7.1 (5.9 to 8.4) | -36.5 (-50.3 to -20.3) | 197.6 (169.9 to 232.7) | -35.9 (-48.7 to -20.1) |
| Cambodia | 22.1 (18.5 to 26.6) | 20.6 (6.1 to 33.2) | 16.7 (13.6 to 20.5) | 0.7 (0.4 to 1.1) | 3.5 (2.5 to 4.5) | -6.6 (-31.4 to 55.4) | 74.5 (53.0 to 103.5) | -9.0 (-36.1 to 50.1) |
| Indonesia | 32.1 (26.1 to 38.6) | 13.4 (4.6 to 22.5) | 18.8 (15.3 to 23.0) | 0.3 (0.2 to 0.5) | 4.6 (3.6 to 5.7) | 23.7 (-11.5 to 81.3) | 95.7 (74.7 to 121.4) | 10.8 (-20.7 to 57.2) |
| Lao PDR | 22.4 (18.7 to 27.4) | 25.2 (13.6 to 39.3) | 17.0 (13.8 to 20.7) | 0.7 (0.5 to 0.9) | 4.3 (3.1 to 5.9) | -18.9 (-44.7 to 38.5) | 96.4 (70.2 to 133.8) | -17.8 (-47.1 to 39.3) |
| Malaysia | 47.7 (40.1 to 56.6) | -7.2 (-16.3 to 3.1) | 17.1 (13.9 to 20.9) | 0.8 (0.6 to 1.0) | 3.4 (2.8 to 4.0) | -38.3 (-55.2 to -7.4) | 83.7 (71.5 to 96.7) | -38.2 (-52.2 to -17.6) |
| Myanmar | 26.0 (21.5 to 31.7) | 27.0 (12.2 to 41.8) | 16.8 (13.6 to 20.5) | -0.6 (-0.8 to -0.4) | 4.4 (3.4 to 5.8) | -18.2 (-43.6 to 26.1) | 102.7 (79.5 to 136.5) | -17.1 (-41.3 to 26.5) |
| Philippines | 34.9 (28.4 to 42.2) | 0.9 (-6.2 to 8.7) | 18.7 (15.1 to 22.8) | -0.8 (-4.0 to 2.6) | 4.5 (2.9 to 5.3) | -36.5 (-48.1 to -18.1) | 103.8 (76.4 to 120.3) | -21.9 (-34.8 to -5.1) |
| Singapore | 66.5 (54.5 to 79.8) | -5.8 (-14.6 to 2.9) | 18.2 (14.9 to 22.2) | 0.4 (0.1 to 0.8) | 1.8 (1.6 to 2.0) | -76.6 (-78.8 to -74.4) | 55.9 (51.2 to 61.1) | -77.6 (-79.6 to -75.4) |
| Thailand | 21.1 (17.6 to 25.2) | 74.9 (56.0 to 94.1) | 16.9 (13.7 to 20.6) | -0.0 (-0.1 to 0.1) | 0.9 (0.7 to 1.1) | 26.6 (-28.5 to 105.1) | 28.8 (19.8 to 37.0) | 45.5 (-21.3 to 126.8) |
| Timor-Leste | 27.5 (22.7 to 33.1) | 14.3 (3.1 to 26.5) | 17.0 (13.8 to 20.8) | -0.4 (-0.6 to -0.2) | 4.6 (3.1 to 6.3) | 0.8 (-31.3 to 53.9) | 101.8 (68.7 to 140.1) | 1.7 (-30.5 to 50.8) |
| Viet Nam | 43.0 (35.6 to 51.1) | 26.3 (14.2 to 39.6) | 16.8 (13.7 to 20.6) | 0.9 (0.7 to 1.1) | 4.2 (3.1 to 5.3) | -6.9 (-40.8 to 45.1) | 85.1 (64.4 to 107.5) | -5.5 (-36.7 to 44.4) |
| **South Asia** | **50.0 (41.1 to 58.5)** | **27.0 (18.4 to 37.1)** | **16.3 (13.2 to 20.0)** | **-0.6 (-0.8 to -0.4)** | **5.3 (4.3 to 6.6)** | **3.4 (-18.0 to 56.8)** | **141.9 (115.2 to 173.6)** | **-3.8 (-22.4 to 40.6)** |
| Afghanistan | 19.3 (15.6 to 23.2) | 28.8 (16.7 to 42.3) | 11.9 (9.5 to 14.7) | 0.2 (-0.4 to 1.0) | 3.2 (1.6 to 6.2) | -0.7 (-35.6 to 52.4) | 96.0 (52.9 to 169.0) | -10.7 (-41.0 to 35.5) |
| Bangladesh | 55.3 (45.9 to 66.0) | 32.5 (18.2 to 48.4) | 14.9 (12.1 to 18.2) | -1.0 (-1.3 to -0.8) | 6.7 (4.5 to 9.7) | -9.5 (-37.5 to 56.0) | 160.9 (109.1 to 232.2) | -13.4 (-42.8 to 45.3) |
| Bhutan | 58.3 (49.0 to 67.9) | 49.3 (35.0 to 64.2) | 14.9 (12.1 to 18.3) | 0.2 (-0.0 to 0.5) | 6.0 (4.1 to 8.7) | -0.9 (-34.1 to 86.8) | 148.1 (101.2 to 220.3) | -1.8 (-36.3 to 79.7) |
| India | 49.6 (40.4 to 58.9) | 28.1 (18.8 to 39.6) | 16.5 (13.3 to 20.2) | -0.5 (-0.7 to -0.4) | 5.0 (4.1 to 6.2) | 6.2 (-16.5 to 61.5) | 133.4 (108.4 to 163.5) | -4.7 (-25.5 to 40.2) |
| Iran | 41.1 (32.8 to 50.8) | 29.2 (20.8 to 39.0) | 12.7 (10.2 to 15.8) | -0.1 (-0.3 to 0.0) | 1.5 (1.3 to 2.1) | -33.9 (-49.4 to -13.3) | 42.4 (37.5 to 50.1) | -46.5 (-60.1 to -26.1) |
| Maldives | 39.2 (33.0 to 46.5) | 50.3 (34.9 to 64.4) | 17.6 (14.3 to 21.4) | 1.2 (0.3 to 2.2) | 2.9 (2.1 to 3.7) | -33.3 (-57.3 to 17.6) | 57.8 (44.4 to 70.8) | -39.6 (-65.6 to 3.2) |
| Nepal | 52.7 (43.9 to 61.7) | 35.8 (23.0 to 49.5) | 14.8 (12.0 to 18.0) | -0.9 (-1.2 to -0.7) | 5.8 (4.1 to 8.9) | -6.3 (-36.6 to 56.5) | 144.3 (101.2 to 218.7) | -11.4 (-37.7 to 40.6) |
| Pakistan | 48.0 (38.8 to 57.3) | 15.2 (5.1 to 27.1) | 16.6 (13.3 to 20.3) | -0.7 (-0.9 to -0.5) | 6.5 (4.4 to 8.7) | 15.5 (-14.1 to 83.2) | 175.7 (123.9 to 228.0) | 8.9 (-15.7 to 63.4) |
| Sri Lanka | 43.5 (35.6 to 51.2) | -35.0 (-42.5 to -26.3) | 16.8 (13.6 to 20.6) | -3.5 (-5.0 to -2.4) | 3.1 (2.0 to 5.6) | -75.1 (-84.5 to -34.0) | 85.5 (56.2 to 137.5) | -73.5 (-82.5 to -39.0) |
| Armenia | 56.5 (47.3 to 66.7) | -7.2 (-16.9 to 2.4) | 18.2 (14.8 to 22.2) | 1.3 (-1.9 to 4.1) | 4.6 (3.8 to 5.7) | -39.6 (-56.3 to -17.0) | 130.8 (107.3 to 161.4) | -35.4 (-53.6 to -8.2) |
| Azerbaijan | 101.6 (84.3 to 119.7) | 25.9 (14.1 to 37.3) | 16.6 (13.3 to 20.5) | 1.8 (1.5 to 2.2) | 14.2 (8.7 to 21.2) | 1.7 (-41.3 to 74.1) | 437.3 (252.3 to 650.3) | 3.3 (-44.3 to 74.6) |
| Bahrain | 97.0 (81.2 to 113.7) | 23.8 (13.8 to 33.9) | 12.3 (9.8 to 15.2) | 1.2 (0.9 to 1.5) | 5.1 (4.1 to 6.2) | -43.7 (-56.0 to -26.0) | 116.5 (93.6 to 140.2) | -46.8 (-58.9 to -30.7) |
| Cyprus | 53.5 (43.8 to 64.0) | 12.7 (1.4 to 24.7) | 17.1 (14.1 to 20.9) | 0.2 (-0.1 to 0.4) | 3.8 (2.7 to 4.8) | -56.9 (-70.1 to -35.0) | 67.7 (54.6 to 83.2) | -55.2 (-67.8 to -37.8) |
| Georgia | 46.0 (38.3 to 54.9) | 20.6 (8.7 to 34.1) | 15.1 (12.2 to 18.5) | 0.8 (-1.9 to 2.8) | 6.3 (5.0 to 7.7) | 16.8 (-16.8 to 62.8) | 183.6 (145.6 to 227.9) | 20.9 (-15.2 to 69.6) |
| Iraq | 67.3 (56.2 to 79.6) | 17.8 (7.7 to 28.7) | 11.9 (9.5 to 14.8) | -0.7 (-1.2 to -0.4) | 5.0 (3.8 to 7.3) | -24.9 (-48.8 to 6.4) | 147.8 (117.7 to 189.8) | -35.7 (-52.4 to -9.0) |
| Israel | 39.3 (32.0 to 46.4) | -17.8 (-25.7 to -9.2) | 16.4 (13.4 to 20.3) | 0.3 (0.1 to 0.5) | 1.1 (1.0 to 1.2) | -67.7 (-71.4 to -63.7) | 25.4 (22.8 to 28.3) | -69.0 (-72.3 to -65.3) |
| Jordan | 17.8 (14.5 to 21.2) | -0.5 (-9.5 to 9.6) | 12.0 (9.6 to 14.9) | 0.5 (0.4 to 0.7) | 0.5 (0.4 to 0.6) | -55.7 (-68.3 to -37.4) | 15.7 (12.6 to 19.1) | -56.8 (-69.4 to -37.7) |
| Kuwait | 32.8 (26.6 to 40.0) | -31.2 (-37.8 to -23.5) | 12.1 (9.6 to 14.9) | -1.3 (-2.0 to -0.8) | 0.9 (0.8 to 1.1) | -65.5 (-71.7 to -58.4) | 28.4 (23.8 to 33.4) | -69.8 (-74.3 to -64.5) |
| Lebanon | 38.7 (32.3 to 46.4) | 40.2 (28.4 to 53.2) | 11.8 (9.5 to 14.7) | 0.1 (-0.2 to 0.4) | 1.3 (1.0 to 1.7) | -40.6 (-68.9 to 11.0) | 33.2 (27.8 to 40.4) | -46.6 (-69.7 to -7.2) |
| Oman | 81.1 (65.7 to 97.0) | 6.9 (-3.4 to 17.6) | 12.2 (9.7 to 15.1) | -0.0 (-0.3 to 0.2) | 5.6 (4.0 to 7.2) | -40.5 (-64.1 to -0.5) | 150.4 (98.6 to 195.9) | -42.5 (-67.7 to -2.0) |
| Palestine | 62.1 (51.7 to 74.0) | 22.4 (12.8 to 33.3) | 11.9 (9.5 to 14.7) | 0.6 (0.4 to 0.9) | 3.8 (2.9 to 4.8) | -33.8 (-52.5 to -1.6) | 94.8 (74.5 to 123.0) | -37.6 (-56.2 to -11.9) |
| Qatar | 87.4 (71.9 to 103.1) | 7.7 (-2.5 to 18.3) | 12.5 (10.0 to 15.5) | -0.1 (-0.2 to -0.0) | 3.2 (2.0 to 4.4) | -63.4 (-76.3 to -39.6) | 76.8 (48.0 to 101.9) | -61.0 (-75.3 to -32.5) |
| Saudi Arabia | 69.5 (58.1 to 82.7) | 4.8 (-4.8 to 14.8) | 12.2 (9.8 to 15.1) | 0.1 (-0.1 to 0.3) | 5.2 (4.0 to 6.8) | -38.2 (-59.7 to 0.6) | 134.4 (103.1 to 172.9) | -44.3 (-62.2 to -10.9) |
| Syrian Arab Republic | 38.2 (30.1 to 47.0) | 31.0 (16.2 to 46.9) | 11.8 (9.4 to 14.7) | -1.1 (-1.6 to -0.7) | 2.4 (1.6 to 4.3) | -28.1 (-53.6 to 12.1) | 76.4 (48.0 to 121.3) | -37.8 (-62.0 to 1.5) |
| Turkey | 52.6 (42.3 to 64.9) | 30.7 (16.4 to 45.5) | 12.3 (9.8 to 15.2) | 0.0 (-0.0 to 0.1) | 1.3 (1.1 to 1.9) | -45.8 (-62.4 to -16.3) | 45.4 (37.8 to 55.3) | -51.8 (-69.6 to -22.7) |
| United Arab Emirates | 109.9 (91.8 to 128.6) | 12.4 (2.7 to 23.4) | 12.6 (10.1 to 15.7) | 1.6 (0.9 to 2.5) | 6.7 (5.1 to 8.4) | -34.5 (-54.9 to -9.8) | 157.1 (125.8 to 194.8) | -42.1 (-58.2 to -20.8) |
| Yemen | 26.0 (21.4 to 32.0) | 36.8 (22.1 to 51.3) | 11.9 (9.5 to 14.7) | 0.2 (-0.1 to 0.5) | 2.8 (1.6 to 5.2) | -13.1 (-43.2 to 45.5) | 85.2 (53.8 to 138.0) | -19.1 (-48.7 to 36.3) |

ASR: Age-standardized rate; UI: Uncertainty interval; DALYs: Disability-adjusted life years

| **Table S12. Pulmonary arterial hypertension burden by country.** | | | | | | | | |
| --- | --- | --- | --- | --- | --- | --- | --- | --- |
|  | **Prevalence (95%UI)** | | **Incidence (95%UI)** | | **Death (95%UI)** | | **DALYs (95%UI)** | |
| **Location** | **ASR (per 100,000) 2021** | **Percent change, 1990-2021** | **ASR (per 100,000) 2021** | **Percent change, 1990-2021** | **ASR (per 100,000) 2021** | **Percent change, 1990-2021** | **ASR (per 100,000) 2021** | **Percent change, 1990-2021** |
| **Central Asia** | **2.3 (1.9 to 2.9)** | **-5.2 (-7.3 to -2.7)** | **0.4 (0.4 to 0.5)** | **6.8 (4.7 to 8.8)** | **0.4 (0.3 to 0.5)** | **3.7 (-12.9 to 25.9)** | **12.9 (10.6 to 15.6)** | **-8.7 (-25.3 to 12.8)** |
| Kazakhstan | 2.3 (1.9 to 2.8) | -7.2 (-11.9 to -2.5) | 0.4 (0.4 to 0.5) | 8.6 (3.7 to 13.6) | 0.0 (0.0 to 0.1) | 5.8 (-24.6 to 57.3) | 1.5 (1.2 to 1.7) | -14.6 (-34.4 to 14.5) |
| Kyrgyzstan | 2.7 (2.2 to 3.3) | 12.6 (6.8 to 18.9) | 0.4 (0.3 to 0.5) | -8.3 (-12.4 to -3.4) | 0.1 (0.0 to 0.1) | -46.5 (-59.2 to -31.5) | 1.8 (1.5 to 2.2) | -49.3 (-61.8 to -33.1) |
| Tajikistan | 2.3 (1.8 to 2.8) | -3.2 (-7.9 to 1.4) | 0.4 (0.4 to 0.5) | 4.4 (-0.1 to 9.5) | 0.8 (0.5 to 1.1) | -33.6 (-55.6 to -6.5) | 27.4 (17.7 to 37.4) | -30.9 (-52.0 to 2.2) |
| Turkmenistan | 2.2 (1.8 to 2.6) | -3.6 (-9.4 to 1.2) | 0.5 (0.4 to 0.6) | 3.8 (-1.3 to 8.5) | 0.2 (0.2 to 0.3) | 27.0 (-8.6 to 86.5) | 7.9 (6.0 to 10.3) | 24.5 (-9.8 to 78.3) |
| Uzbekistan | 2.1 (1.7 to 2.6) | -15.2 (-20.4 to -9.3) | 0.5 (0.4 to 0.6) | 15.5 (10.0 to 21.2) | 0.4 (0.3 to 0.5) | -19.1 (-43.7 to 16.3) | 12.2 (9.8 to 14.8) | -28.8 (-46.2 to -6.9) |
| **East Asia** | **2.2 (1.8 to 2.7)** | **7.6 (6.3 to 9.1)** | **0.5 (0.4 to 0.6)** | **-2.0 (-3.1 to -0.9)** | **0.4 (0.3 to 0.5)** | **-30.8 (-55.2 to 1.2)** | **8.8 (6.0 to 11.0)** | **-44.0 (-65.0 to -20.1)** |
| China | 2.2 (1.8 to 2.8) | 8.1 (6.7 to 9.7) | 0.5 (0.4 to 0.6) | -2.3 (-3.4 to -1.1) | 0.4 (0.3 to 0.5) | -31.5 (-55.9 to 0.9) | 8.9 (6.0 to 11.1) | -44.7 (-65.5 to -20.7) |
| Japan | 3.1 (2.5 to 3.8) | -6.9 (-8.2 to -5.6) | 0.3 (0.3 to 0.4) | 7.4 (6.1 to 8.8) | 0.3 (0.3 to 0.3) | -1.9 (-10.1 to 5.6) | 10.7 (10.3 to 11.5) | -24.1 (-28.3 to -19.0) |
| Mongolia | 2.1 (1.7 to 2.6) | 16.0 (10.6 to 21.9) | 0.5 (0.4 to 0.6) | 24.7 (19.2 to 31.7) | 1.6 (0.9 to 2.0) | -8.5 (-41.1 to 47.3) | 43.9 (25.6 to 56.5) | -13.4 (-43.0 to 37.1) |
| North Korea | 1.9 (1.6 to 2.4) | -4.9 (-10.8 to 0.5) | 0.5 (0.4 to 0.6) | 4.5 (-0.4 to 10.4) | 0.3 (0.2 to 0.6) | 1.6 (-28.9 to 46.7) | 8.0 (5.7 to 13.4) | -5.0 (-34.2 to 36.0) |
| South Korea | 2.9 (2.4 to 3.6) | -3.5 (-8.8 to 0.8) | 0.3 (0.2 to 0.4) | 2.2 (-3.1 to 7.2) | 0.1 (0.0 to 0.2) | -57.3 (-74.8 to -27.4) | 2.4 (1.7 to 4.7) | -63.2 (-78.5 to -36.1) |
| Taiwan [Province of China] | 2.3 (1.8 to 2.8) | -4.6 (-10.3 to -0.3) | 0.5 (0.4 to 0.6) | 5.5 (0.2 to 10.2) | 0.2 (0.2 to 0.2) | 92.6 (71.4 to 114.2) | 4.2 (3.8 to 4.5) | 74.2 (58.1 to 89.7) |
| **Southeast Asia** | **1.9 (1.5 to 2.3)** | **7.3 (5.8 to 9.0)** | **0.6 (0.5 to 0.7)** | **5.4 (4.2 to 6.7)** | **0.1 (0.1 to 0.3)** | **-20.9 (-39.4 to 10.2)** | **4.7 (3.4 to 9.2)** | **-26.3 (-42.8 to 0.3)** |
| Brunei Darussalam | 2.0 (1.6 to 2.5) | -3.0 (-7.6 to 1.3) | 0.3 (0.3 to 0.4) | 2.9 (-2.2 to 7.9) | 0.3 (0.2 to 0.3) | -43.1 (-57.9 to -17.8) | 11.4 (7.2 to 14.9) | -44.5 (-58.8 to -17.7) |
| Cambodia | 1.6 (1.3 to 1.9) | -0.0 (-5.7 to 5.7) | 0.6 (0.5 to 0.7) | -1.0 (-5.5 to 3.5) | 0.1 (0.1 to 0.4) | -20.9 (-46.3 to 26.3) | 4.9 (3.1 to 10.1) | -26.1 (-54.2 to 22.1) |
| Indonesia | 1.7 (1.4 to 2.1) | 16.7 (14.4 to 19.1) | 0.6 (0.5 to 0.7) | 17.0 (15.1 to 19.1) | 0.1 (0.1 to 0.4) | -15.2 (-37.8 to 20.7) | 4.6 (3.0 to 10.4) | -23.0 (-42.8 to 10.4) |
| Lao PDR | 2.0 (1.6 to 2.4) | 16.4 (9.5 to 23.7) | 0.7 (0.6 to 0.8) | 4.5 (-0.7 to 9.2) | 0.2 (0.1 to 0.4) | -33.3 (-54.0 to 2.1) | 6.7 (3.7 to 14.0) | -33.2 (-55.9 to 13.8) |
| Malaysia | 1.9 (1.5 to 2.3) | -0.6 (-5.8 to 5.1) | 0.5 (0.4 to 0.6) | -6.1 (-10.8 to -1.7) | 0.1 (0.0 to 0.3) | -16.2 (-46.7 to 19.1) | 2.5 (1.7 to 6.6) | -23.1 (-46.5 to 4.0) |
| Myanmar | 1.6 (1.3 to 1.9) | 1.3 (-4.7 to 7.4) | 0.6 (0.5 to 0.7) | -1.1 (-5.6 to 4.0) | 0.2 (0.1 to 0.4) | -30.1 (-50.8 to 9.9) | 6.8 (4.4 to 13.8) | -33.3 (-54.0 to 9.4) |
| Philippines | 2.2 (1.8 to 2.7) | -4.7 (-5.8 to -3.7) | 0.6 (0.5 to 0.8) | 1.5 (0.7 to 2.2) | 0.1 (0.0 to 0.2) | -29.6 (-46.4 to -7.0) | 3.3 (2.4 to 7.5) | -33.6 (-47.0 to -14.3) |
| Singapore | 2.2 (1.8 to 2.8) | -10.4 (-15.7 to -5.2) | 0.4 (0.3 to 0.4) | 11.2 (-2.2 to 7.9) | 0.1 (0.1 to 0.1) | -61.6 (-65.8 to -57.1) | 3.8 (3.4 to 4.3) | -67.1 (-71.2 to -57.1) |
| Thailand | 2.1 (1.7 to 2.6) | 7.8 (3.0 to 13.7) | 0.5 (0.4 to 0.6) | -2.2 (-6.3 to 2.3) | 0.1 (0.0 to 0.3) | -24.2 (-48.6 to 14.1) | 3.1 (2.1 to 7.5) | -25.4 (-46.7 to 7.0) |
| Timor-Leste | 1.8 (1.5 to 2.2) | 4.6 (-1.3 to 10.4) | 0.6 (0.5 to 0.7) | -3.3 (-7.7 to 1.4) | 0.2 (0.1 to 0.4) | -18.4 (-41.5 to 20.9) | 6.3 (3.8 to 13.1) | -21.5 (-45.4 to 15.0) |
| Viet Nam | 1.9 (1.5 to 2.3) | 1.6 (-3.7 to 7.2) | 0.5 (0.4 to 0.6) | -2.3 (-6.5 to 2.5) | 0.1 (0.1 to 0.4) | -21.6 (-51.3 to 25.4) | 3.8 (2.1 to 9.6) | -29.5 (-53.9 to 9.9) |
| **South Asia** | **1.7 (1.4 to 2.1)** | **7.9 (6.3 to 9.5)** | **0.6 (0.5 to 0.7)** | **-0.3 (-1.4 to 0.7)** | **0.3 (0.2 to 0.4)** | **-19.3 (-38.5 to 3.7)** | **8.5 (6.0 to 12.5)** | **-30.2 (-44.3 to -9.7)** |
| Afghanistan | 1.5 (1.2 to 1.8) | 2.1 (-3.8 to 8.7) | 0.6 (0.5 to 0.7) | -1.2 (-5.8 to 4.0) | 0.7 (0.2 to 1.1) | 64.0 (-35.3 to 165.1) | 25.3 (11.0 to 39.0) | 39.0 (-43.3 to 125.7) |
| Bangladesh | 1.8 (1.5 to 2.2) | 42.1 (32.9 to 51.6) | 0.5 (0.4 to 0.6) | 15.0 (9.5 to 20.9) | 0.3 (0.1 to 0.6) | -28.7 (-50.6 to 11.1) | 8.7 (4.8 to 13.7) | -34.8 (-53.6 to -1.9) |
| Bhutan | 1.6 (1.3 to 2.0) | -13.6 (-18.7 to -8.1) | 0.7 (0.5 to 0.8) | 11.3 (6.4 to 16.4) | 0.3 (0.2 to 0.5) | -21.5 (-45.9 to 28.7) | 9.3 (5.1 to 14.2) | -35.1 (-58.9 to 7.1) |
| India | 1.7 (1.4 to 2.1) | 6.9 (5.4 to 8.7) | 0.6 (0.5 to 0.7) | -2.8 (-4.0 to -1.7) | 0.2 (0.2 to 0.4) | -19.6 (-40.9 to 8.4) | 7.7 (5.5 to 11.4) | -33.9 (-48.1 to -9.9) |
| Iran | 2.1 (1.7 to 2.6) | 2.9 (1.3 to 4.6) | 0.5 (0.4 to 0.5) | -1.8 (-2.9 to -0.7) | 0.6 (0.4 to 0.7) | -56.4 (-69.2 to -36.2) | 16.3 (12.9 to 18.7) | -68.1 (-76.1 to -50.9) |
| Maldives | 2.1 (1.7 to 2.6) | 18.2 (11.2 to 25.2) | 0.5 (0.4 to 0.6) | -13.5 (-17.8 to -9.5) | 0.2 (0.1 to 0.3) | -63.5 (-74.0 to -43.9) | 7.5 (6.0 to 10.3) | -66.1 (-77.6 to -42.3) |
| Nepal | 1.6 (1.3 to 1.9) | 6.8 (0.5 to 13.8) | 0.6 (0.5 to 0.7) | 8.2 (3.0 to 13.3) | 0.3 (0.1 to 0.6) | -18.8 (-43.3 to 18.3) | 9.4 (5.2 to 15.0) | -36.1 (-59.1 to -5.7) |
| Pakistan | 1.4 (1.1 to 1.7) | -8.4 (-12.2 to -4.4) | 0.6 (0.5 to 0.7) | 8.3 (5.3 to 11.7) | 0.3 (0.2 to 0.6) | -6.2 (-32.0 to 28.7) | 12.0 (7.9 to 18.3) | -17.0 (-38.0 to 12.0) |
| Sri Lanka | 2.5 (2.0 to 3.1) | 6.1 (0.2 to 12.0) | 0.6 (0.5 to 0.7) | -3.3 (-7.6 to 1.3) | 0.4 (0.3 to 0.6) | -0.5 (-49.8 to 68.0) | 15.1 (9.5 to 21.9) | -12.1 (-54.4 to 48.7) |
| Armenia | 2.8 (2.3 to 3.4) | -3.5 (-7.7 to 1.6) | 0.4 (0.3 to 0.5) | 6.4 (2.1 to 11.5) | 0.1 (0.1 to 0.1) | -69.1 (-77.4 to -58.9) | 2.2 (1.8 to 2.5) | -73.2 (-79.2 to -66.6) |
| Azerbaijan | 2.7 (2.2 to 3.2) | 21.0 (14.4 to 27.7) | 0.4 (0.3 to 0.5) | -13.9 (-17.6 to -9.7) | 0.6 (0.4 to 0.8) | -18.1 (-44.5 to 22.0) | 17.6 (10.7 to 27.7) | -21.9 (-51.8 to 28.7) |
| Bahrain | 1.8 (1.5 to 2.3) | -1.3 (-6.3 to 4.1) | 0.5 (0.4 to 0.6) | -0.8 (-5.6 to 4.7) | 0.2 (0.1 to 0.3) | -18.4 (-56.2 to 23.6) | 4.5 (2.3 to 8.0) | -31.6 (-65.3 to 2.4) |
| Cyprus | 4.2 (3.5 to 5.1) | 17.6 (11.1 to 24.2) | 0.4 (0.3 to 0.5) | -16.8 (-20.9 to -12.6) | 0.8 (0.4 to 1.0) | -61.5 (-71.0 to -42.3) | 17.4 (9.6 to 21.8) | -66.7 (-74.4 to -50.6) |
| Georgia | 2.6 (2.1 to 3.1) | -2.6 (-8.0 to 2.3) | 0.4 (0.3 to 0.5) | 4.3 (-0.5 to 9.6) | 1.0 (0.8 to 1.3) | 172.0 (94.2 to 280.6) | 27.8 (22.2 to 34.8) | 110.5 (51.0 to 198.1) |
| Iraq | 1.9 (1.5 to 2.3) | 9.4 (3.2 to 15.9) | 0.6 (0.5 to 0.7) | -7.0 (-11.2 to -2.5) | 0.1 (0.1 to 0.4) | -39.6 (-62.4 to -5.9) | 5.2 (2.8 to 10.1) | -53.3 (-69.0 to -25.2) |
| Israel | 4.4 (3.6 to 5.3) | -2.4 (-7.1 to 2.5) | 0.6 (0.5 to 0.8) | -1.6 (-6.4 to 2.9) | 0.3 (0.2 to 0.3) | -43.1 (-56.8 to -31.7) | 7.2 (6.5 to 7.8) | -55.8 (-67.2 to -48.4) |
| Jordan | 2.3 (1.8 to 2.8) | 2.0 (-3.0 to 7.5) | 0.5 (0.4 to 0.6) | -1.8 (-6.6 to 4.4) | 0.1 (0.0 to 0.2) | -2.3 (-61.7 to 109.5) | 2.5 (1.2 to 6.9) | -11.4 (-63.8 to 65.1) |
| Kuwait | 2.2 (1.7 to 2.6) | 5.3 (0.4 to 9.9) | 0.4 (0.3 to 0.5) | -0.1 (-4.8 to 4.9) | 0.1 (0.1 to 0.2) | 90.6 (58.6 to 128.2) | 6.5 (5.4 to 7.7) | 56.1 (27.3 to 91.3) |
| Lebanon | 2.1 (1.7 to 2.6) | 1.2 (-3.7 to 6.4) | 0.5 (0.4 to 0.6) | -0.7 (-6.3 to 4.6) | 0.4 (0.3 to 0.6) | -58.0 (-73.9 to -25.9) | 11.3 (8.9 to 14.5) | -64.8 (-78.1 to -36.7) |
| Oman | 2.0 (1.6 to 2.5) | 1.1 (-4.2 to 6.7) | 0.5 (0.4 to 0.7) | 1.0 (-3.3 to 5.3) | 0.1 (0.0 to 0.2) | 6.8 (-64.9 to 160.9) | 3.9 (1.5 to 7.2) | -6.7 (-70.3 to 107.6) |
| Palestine | 1.9 (1.5 to 2.3) | -13.2 (-18.4 to -7.4) | 0.5 (0.4 to 0.6) | 11.0 (5.6 to 16.6) | 0.1 (0.1 to 0.4) | -43.5 (-61.0 to -13.2) | 4.7 (2.0 to 11.9) | -54.1 (-69.8 to -29.3) |
| Qatar | 1.9 (1.5 to 2.3) | 1.7 (-3.7 to 7.3) | 0.5 (0.4 to 0.6) | -0.8 (-5.5 to 3.7) | 0.1 (0.1 to 0.2) | -21.8 (-68.7 to 33.1) | 3.7 (2.0 to 5.6) | -29.7 (-72.6 to 15.5) |
| Saudi Arabia | 1.9 (1.6 to 2.3) | 17.6 (11.0 to 25.0) | 0.5 (0.4 to 0.6) | -12.6 (-17.0 to -7.9) | 0.1 (0.0 to 0.2) | -42.0 (-71.2 to 1.8) | 2.6 (1.4 to 5.4) | -53.6 (-73.6 to -21.9) |
| Syrian Arab Republic | 2.0 (1.6 to 2.4) | 4.9 (-0.8 to 10.9) | 0.6 (0.5 to 0.7) | -0.4 (-4.9 to 4.3) | 0.1 (0.0 to 0.3) | -41.0 (-64.9 to -4.3) | 2.3 (1.1 to 11.6) | -48.9 (-76.0 to -13.9) |
| Turkey | 2.6 (2.1 to 3.2) | 39.8 (31.7 to 47.9) | 0.5 (0.4 to 0.6) | 8.4 (3.5 to 13.5) | 0.5 (0.4 to 0.7) | -58.2 (-72.4 to -31.8) | 16.6 (12.9 to 20.6) | -69.3 (-78.9 to -43.5) |
| United Arab Emirates | 1.7 (1.4 to 2.0) | -5.1 (-10.5 to 0.8) | 0.5 (0.4 to 0.6) | 1.2 (-4.4 to 6.7) | 0.4 (0.2 to 0.5) | -48.1 (-66.5 to -16.1) | 10.3 (5.6 to 13.0) | -61.5 (-73.5 to -39.4) |
| Yemen | 2.1 (1.7 to 2.6) | -14.0 (-19.1 to -8.7) | 0.7 (0.5 to 0.8) | -12.1 (-17.0 to -7.4) | 0.6 (0.2 to 0.9) | 37.5 (-45.1 to 142.7) | 19.3 (9.1 to 28.9) | 15.3 (-51.9 to 82.0) |

ASR: Age-standardized rate; UI: Uncertainty interval; DALYs: Disability-adjusted life years

| **Table S13. Aortic aneurysm burden by country** | | | | |
| --- | --- | --- | --- | --- |
|  | **Death (95%UI)** | | **DALYs (95%UI)** | |
| **Location** | **ASR (per 100,000) 2021** | **Percent change, 1990-2021** | **ASR (per 100,000) 2021** | **Percent change, 1990-2021** |
| **Central Asia** | **2.0 (1.8 to 2.2)** | **110.1 (71.5 to 150.9)** | **42.7 (37.8 to 47.9)** | **80.9 (47.1 to 115.6)** |
| Kazakhstan | 2.2 (1.8 to 2.7) | 52.8 (8.1 to 112.2) | 52.8 (42.1 to 65.0) | 39.4 (-1.2 to 89.2) |
| Kyrgyzstan | 0.8 (0.6 to 1.0) | 185.5 (114.0 to 272.3) | 19.4 (15.4 to 23.7) | 176.0 (108.9 to 255.7) |
| Tajikistan | 0.3 (0.2 to 0.3) | -10.0 (-39.0 to 38.3) | 5.7 (4.1 to 7.8) | -11.2 (-45.1 to 37.8) |
| Turkmenistan | 1.9 (1.4 to 2.7) | 83.0 (21.2 to 165.9) | 46.9 (34.6 to 67.4) | 86.5 (21.6 to 177.7) |
| Uzbekistan | 1.0 (0.8 to 1.3) | 385.9 (225.1 to 595.0) | 22.2 (18.0 to 27.4) | 320.6 (199.2 to 472.1) |
| **East Asia** | **0.5 (0.4 to 0.6)** | **40.0 (-1.6 to 92.0)** | **13.3 (10.6 to 17.0)** | **40.1 (-4.6 to 99.6)** |
| China | 0.5 (0.4 to 0.6) | 37.2 (-7.5 to 95.7) | 12.4 (9.5 to 16.1) | 38.8 (-8.9 to 105.7) |
| Japan | 5.1 (4.3 to 5.5) | 73.6 (60.4 to 81.6) | 96.8 (87.8 to 101.6) | 74.4 (66.1 to 80.2) |
| Mongolia | 0.7 (0.5 to 0.9) | 124.9 (50.0 to 251.8) | 16.0 (12.2 to 20.5) | 119.7 (42.9 to 248.5) |
| North Korea | 0.5 (0.4 to 0.7) | -1.3 (-29.0 to 42.0) | 16.1 (11.4 to 22.5) | 2.2 (-29.7 to 53.2) |
| South Korea | 1.9 (1.6 to 2.3) | 4.0 (-34.2 to 68.9) | 33.1 (27.1 to 39.5) | -5.7 (-41.8 to 61.7) |
| Taiwan [Province of China] | 2.4 (2.1 to 2.6) | 76.3 (60.4 to 91.9) | 56.5 (51.8 to 60.5) | 71.2 (55.6 to 85.8) |
| **Southeast Asia** | **1.4 (1.2 to 1.6)** | **34.6 (5.6 to 70.2)** | **25.9 (22.6 to 29.8)** | **32.2 (1.6 to 68.4)** |
| Brunei Darussalam | 4.8 (4.0 to 5.7) | -14.6 (-35.6 to 15.0) | 93.4 (78.8 to 110.2) | -13.0 (-36.0 to 22.0) |
| Cambodia | 0.7 (0.5 to 1.2) | 34.8 (-7.5 to 107.2) | 14.5 (9.2 to 23.2) | 28.7 (-13.9 to 102.6) |
| Indonesia | 1.0 (0.7 to 1.4) | 62.2 (4.4 to 130.4) | 19.4 (13.3 to 26.2) | 50.3 (-3.7 to 118.2) |
| Lao PDR | 0.9 (0.7 to 1.3) | 18.2 (-18.3 to 79.1) | 18.2 (13.2 to 25.3) | 12.7 (-26.0 to 79.7) |
| Malaysia | 4.0 (3.4 to 4.9) | 22.4 (-9.2 to 65.7) | 74.1 (62.2 to 87.8) | 14.8 (-16.2 to 54.4) |
| Myanmar | 1.0 (0.7 to 1.3) | 20.8 (-18.4 to 84.7) | 19.0 (14.5 to 25.1) | 15.3 (-23.3 to 87.6) |
| Philippines | 1.4 (1.1 to 1.6) | 10.6 (-11.1 to 36.1) | 28.4 (23.2 to 33.5) | 18.9 (-5.3 to 47.8) |
| Singapore | 2.2 (1.9 to 2.3) | -18.9 (-26.7 to -11.6) | 39.8 (36.2 to 42.6) | -28.0 (-34.1 to -22.2) |
| Thailand | 2.0 (1.5 to 2.6) | -0.4 (-32.8 to 44.4) | 38.8 (30.1 to 49.3) | 8.9 (-25.5 to 62.3) |
| Timor-Leste | 0.7 (0.5 to 1.2) | 37.0 (-6.3 to 100.3) | 14.2 (9.4 to 21.6) | 34.6 (-8.5 to 98.2) |
| Viet Nam | 1.1 (0.8 to 1.4) | 60.4 (12.1 to 146.4) | 20.1 (14.8 to 27.6) | 58.1 (7.0 to 146.4) |
| **South Asia** | **1.2 (0.9 to 1.8)** | **71.6 (27.9 to 136.1)** | **25.0 (17.8 to 36.6)** | **63.6 (22.3 to 125.7)** |
| Afghanistan | 0.3 (0.2 to 0.4) | 222.8 (127.8 to 371.1) | 7.5 (4.8 to 11.0) | 208.9 (120.8 to 356.8) |
| Bangladesh | 1.2 (0.8 to 2.0) | 48.6 (2.2 to 131.6) | 22.8 (14.8 to 38.4) | 39.3 (-8.6 to 126.7) |
| Bhutan | 1.5 (0.9 to 2.2) | 91.6 (5.3 to 201.2) | 28.2 (17.4 to 42.7) | 83.1 (-2.1 to 200.5) |
| India | 1.2 (0.9 to 1.7) | 77.4 (30.0 to 149.6) | 24.8 (17.5 to 36.2) | 67.0 (24.0 to 134.9) |
| Iran | 0.5 (0.5 to 0.6) | 85.1 (37.6 to 137.5) | 12.1 (10.6 to 13.6) | 79.8 (31.0 to 131.3) |
| Maldives | 0.6 (0.3 to 1.0) | -9.5 (-47.8 to 53.2) | 11.3 (4.8 to 19.5) | -17.3 (-58.9 to 48.0) |
| Nepal | 1.2 (0.8 to 1.8) | 84.6 (24.4 to 177.4) | 23.1 (15.9 to 35.5) | 77.5 (14.8 to 178.1) |
| Pakistan | 1.5 (1.1 to 2.0) | 75.8 (21.9 to 148.4) | 29.7 (21.8 to 41.8) | 71.7 (19.5 to 139.2) |
| Sri Lanka | 0.4 (0.3 to 0.5) | 5.8 (-32.9 to 66.8) | 6.7 (4.6 to 9.4) | 3.1 (-35.6 to 65.7) |
| Armenia | 9.2 (7.6 to 10.8) | 101.4 (51.4 to 170.2) | 192.8 (159.7 to 228.2) | 79.4 (35.1 to 140.4) |
| Azerbaijan | 1.4 (0.9 to 2.1) | 87.7 (15.1 to 189.9) | 32.3 (19.3 to 53.4) | 81.5 (1.2 to 207.9) |
| Bahrain | 0.7 (0.6 to 0.9) | 11.9 (-24.4 to 52.1) | 15.0 (11.5 to 19.5) | 7.1 (-30.6 to 51.4) |
| Cyprus | 4.0 (3.0 to 4.9) | -49.3 (-64.9 to -25.7) | 69.4 (52.4 to 86.3) | -46.5 (-62.9 to -20.7) |
| Georgia | 2.9 (2.4 to 3.3) | 374.2 (260.2 to 510.0) | 69.5 (58.4 to 82.6) | 357.4 (242.4 to 498.6) |
| Iraq | 0.4 (0.3 to 0.5) | 30.5 (-17.8 to 112.0) | 9.5 (6.9 to 12.7) | 19.7 (-27.6 to 104.5) |
| Israel | 1.3 (1.1 to 1.4) | -45.1 (-50.9 to -38.7) | 22.9 (20.7 to 24.8) | -48.6 (-53.7 to -42.7) |
| Jordan | 1.0 (0.8 to 1.2) | -13.7 (-44.1 to 28.0) | 23.3 (17.7 to 30.2) | -17.4 (-48.0 to 25.6) |
| Kuwait | 0.9 (0.7 to 1.1) | -11.2 (-28.2 to 9.9) | 19.5 (16.0 to 24.0) | -22.1 (-37.6 to -1.9) |
| Lebanon | 2.2 (1.8 to 2.8) | -22.6 (-61.0 to 67.4) | 48.7 (40.2 to 60.3) | -32.3 (-64.5 to 39.0) |
| Oman | 0.6 (0.4 to 0.9) | 222.4 (42.3 to 560.7) | 13.5 (7.9 to 23.1) | 206.6 (21.3 to 585.5) |
| Palestine | 0.8 (0.6 to 1.0) | 23.2 (-18.2 to 85.0) | 17.0 (13.3 to 21.2) | 21.2 (-21.7 to 87.6) |
| Qatar | 0.9 (0.6 to 1.5) | -35.5 (-62.7 to 7.1) | 20.3 (12.2 to 32.6) | -34.1 (-64.2 to 18.3) |
| Saudi Arabia | 0.2 (0.2 to 0.3) | 168.5 (74.1 to 330.3) | 5.1 (3.7 to 6.7) | 181.2 (74.8 to 363.0) |
| Syrian Arab Republic | 0.4 (0.3 to 0.6) | 22.5 (-22.6 to 90.1) | 10.3 (7.2 to 14.1) | 20.6 (-32.2 to 101.8) |
| Turkey | 2.3 (1.8 to 2.8) | 10.6 (-28.2 to 67.8) | 54.1 (42.9 to 68.8) | 2.5 (-36.0 to 58.3) |
| United Arab Emirates | 1.5 (1.2 to 1.8) | 14.1 (-19.0 to 74.1) | 31.0 (25.2 to 38.4) | -0.1 (-31.0 to 58.6) |
| Yemen | 0.3 (0.2 to 0.5) | 225.0 (113.7 to 419.0) | 8.1 (4.6 to 12.7) | 206.0 (95.0 to 405.2) |

ASR: Age-standardized rate; UI: Uncertainty interval; DALYs: Disability-adjusted life years

| **Table S14. Lower extremity peripheral arterial disease burden by country.** | | | | | | | | |
| --- | --- | --- | --- | --- | --- | --- | --- | --- |
|  | **Prevalence (95%UI)** | | **Incidence (95%UI)** | | **Death (95%UI)** | | **DALYs (95%UI)** | |
| **Location** | **ASR (per 100,000) 2021** | **Percent change, 1990-2021** | **ASR (per 100,000) 2021** | **Percent change, 1990-2021** | **ASR (per 100,000) 2021** | **Percent change, 1990-2021** | **ASR (per 100,000) 2021** | **Percent change, 1990-2021** |
| **Central Asia** | **975.9 (848.3 to 1136.8)** | **9.2 (7.2 to 11.5)** | **91.6 (78.7 to 106.6)** | **7.5 (5.4 to 9.5)** | **0.5 (0.4 to 0.5)** | **104.5 (61.8 to 155.4)** | **12.5 (9.6 to 16.9)** | **42.6 (24.8 to 68.6)** |
| Kazakhstan | 1102.2 (953.8 to 1275.9) | 10.9 (5.7 to 15.5) | 99.8 (85.1 to 116.0) | 8.1 (2.8 to 13.2) | 0.2 (0.2 to 0.3) | 193.2 (104.2 to 322.2) | 9.0 (6.3 to 13.8) | 38.6 (18.0 to 76.2) |
| Kyrgyzstan | 892.1 (763.0 to 1042.5) | -1.7 (-5.2 to 2.5) | 87.3 (75.0 to 101.9) | -0.2 (-4.8 to 4.2) | 0.6 (0.5 to 0.7) | 85.8 (41.0 to 162.5) | 15.5 (12.2 to 20.0) | 44.8 (17.4 to 88.5) |
| Tajikistan | 845.7 (728.4 to 981.6) | 0.9 (-3.6 to 5.5) | 84.3 (72.8 to 98.3) | 2.9 (-1.6 to 7.5) | 0.2 (0.1 to 0.3) | 3.9 (-44.7 to 90.8) | 7.6 (4.8 to 12.1) | -2.5 (-24.1 to 24.4) |
| Turkmenistan | 956.5 (830.3 to 1114.9) | 5.3 (0.2 to 10.5) | 88.8 (76.5 to 103.4) | 2.4 (-2.5 to 7.6) | 0.3 (0.2 to 0.5) | 39.2 (-7.5 to 95.4) | 11.2 (7.9 to 16.0) | 19.3 (-4.0 to 55.8) |
| Uzbekistan | 878.8 (766.4 to 1029.7) | 17.3 (11.3 to 23.1) | 84.8 (73.2 to 99.7) | 14.4 (8.8 to 20.2) | 0.1 (0.1 to 0.1) | 226.4 (108.0 to 449.6) | 6.3 (3.9 to 10.5) | 38.6 (22.2 to 70.3) |
| **East Asia** | **1336.8 (1156.0 to 1548.9)** | **6.4 (5.3 to 7.8)** | **113.0 (98.0 to 131.1)** | **2.9 (1.6 to 4.1)** | **0.1 (0.1 to 0.2)** | **25.8 (-12.7 to 86.3)** | **8.3 (4.9 to 14.3)** | **-6.9 (-15.2 to 6.0)** |
| China | 1331.1 (1147.5 to 1544.2) | 6.4 (5.2 to 7.8) | 112.7 (97.7 to 130.7) | 2.8 (1.6 to 4.2) | 0.1 (0.1 to 0.2) | 25.5 (-13.2 to 86.7) | 8.4 (4.9 to 14.3) | -7.1 (-15.7 to 6.2) |
| Japan | 1039.4 (1135.6 to 1516.0) | -33.9 (-34.8 to -33.2) | 113.0 (97.5 to 130.2) | -31.7 (-32.7 to -31.0) | 0.4 (0.3 to 0.4） | -40.8 (-43.0 to -38.4) | 9.7 (7.0 to 14.1) | -26.6 (-31.2 to -20.6) |
| Mongolia | 900.3 (780.8 to 1042.7) | 10.1 (5.1 to 15.5) | 86.1 (74.4 to 100.5) | 6.5 (1.8 to 11.4) | 1.2 (0.8 to 1.9) | 293.6 (116.5 to 640.8) | 24.2 (16.4 to 33.5) | 136.9 (59.9 to 261.9) |
| North Korea | 1315.2 (1130.3 to 1522.0) | 5.2 (0.1 to 10.3) | 119.5 (103.7 to 138.8) | 7.2 (2.5 to 12.0) | 0.1 (0.1 to 0.2) | 27.2 (-6.4 to 83.0) | 8.8 (4.9 to 16.0) | 2.1 (-7.0 to 14.3) |
| South Korea | 1132.3 (989.9 to 1314.3) | -28.7 (-32.1 to -25.2) | 100.9 (86.6 to 116.1) | -29.3 (-32.8 to -25.8) | 0.3 (0.2 to 0.3) | -20.1 (-43.2 to 11.1) | 7.9 (5.5 to 11.9) | -36.8 (-46.7 to -23.6) |
| Taiwan [Province of China] | 1656.3 (1455.1 to 1865.1) | 7.7 (-0.7 to 19.6) | 127.5 (113.9 to 144.3) | 2.7 (-3.8 to 11.1) | 0.0 (0.0 to 0.0) | 109.1 (82.5 to 137.4) | 7.9 (4.1 to 14.6) | -2.2 (-10.5 to 9.6) |
| **Southeast Asia** | **1387.5 (1195.2 to 1606.8)** | **7.5 (5.7 to 8.9)** | **122.8 (105.9 to 142.9)** | **4.0 (2.6 to 5.4)** | **0.1 (0.1 to 0.2)** | **58.8 (16.7 to 117.1)** | **9.8 (5.7 to 16.9)** | **6.9 (-1.2 to 21.6)** |
| Brunei Darussalam | 1348.8 (1167.9 to 1561.9) | -28.6 (-31.7 to -25.7) | 116.6 (100.1 to 136.1) | -25.7 (-29.0 to -22.4) | 0.6 (0.5 to 0.7) | -12.8 (-43.3 to 49.6) | 13.9 (10.3 to 19.3) | -25.7 (-40.5 to 1.5) |
| Cambodia | 1378.5 (1181.3 to 1608.7) | 15.0 (9.2 to 21.1) | 127.1 (109.9 to 147.9) | 11.0 (5.4 to 17.0) | 0.1 (0.0 to 0.2) | 37.8 (-35.2 to 158.3) | 9.3 (5.0 to 16.9) | 7.0 (-6.4 to 24.9) |
| Indonesia | 1459.7 (1254.6 to 1691.8) | 13.1 (11.1 to 15.0) | 128.6 (111.1 to 149.3) | 9.1 (7.4 to 10.8) | 0.2 (0.1 to 0.2) | 104.5 (19.9 to 254.3) | 10.9 (6.4 to 18.7) | 17.7 (4.0 to 41.7) |
| Lao PDR | 1352.4 (1166.3 to 1584.8) | 5.6 (0.9 to 10.1) | 122.7 (106.0 to 143.1) | 1.9 (-2.5 to 7.1) | 0.2 (0.1 to 0.2) | 77.3 (10.7 to 264.8) | 10.7 (6.2 to 18.5) | 7.5 (-4.5 to 29.3) |
| Malaysia | 1427.2 (1220.3 to 1643.1) | 8.6 (3.8 to 13.3) | 121.8 (105.0 to 142.0) | 5.7 (1.1 to 10.2) | 0.1 (0.1 to 0.2) | 59.6 (17.0 to 139.2) | 9.6 (5.7 to 16.4) | 5.6 (-4.5 to 21.2) |
| Myanmar | 1353.4 (1173.4 to 1581.4) | -6.6 (-10.7 to -2.5) | 122.8 (105.8 to 143.8) | -9.8 (-13.9 to -5.8) | 0.1 (0.1 to 0.2) | 21.8 (-23.6 to 119.7) | 10.3 (6.2 to 17.7) | -10.2 (-21.9 to 5.2) |
| Philippines | 1398.1 (1209.3 to 1619.8) | 2.8 (2.0 to 3.8) | 123.8 (107.0 to 144.2) | 0.6 (-0.3 to 1.6) | 0.2 (0.2 to 0.2) | 22.1 (-3.1 to 64.9) | 11.8 (7.5 to 19.4) | 7.2 (-1.4 to 23.5) |
| Singapore | 1065.4 (916.3 to 1226.1) | -28.9 (-32.3 to -25.8) | 93.5 (80.1 to 107.8) | -29.6 (-32.9 to -26.7) | 0.7 (0.6 to 0.8) | -10.6 (-21.3 to 0.5) | 13.5 (11.0 to 17.3) | -28.0 (-33.8 to -21.5) |
| Thailand | 1237.2 (1061.8 to 1433.8) | -1.6 (-5.7 to 2.5) | 108.0 (92.2 to 125.7) | -3.4 (-7.2 to 1.1) | 0.0 (0.0 to 0.1) | 79.6 (18.8 to 174.4) | 6.8 (3.6 to 12.4) | -8.0 (-15.1 to 2.1) |
| Timor-Leste | 1272.6 (1094.9 to 1482.8) | 13.3 (8.7 to 18.4) | 116.3 (100.1 to 135.7) | 10.8 (6.2 to 15.4) | 0.1 (0.1 to 0.2) | 74.2 (6.2 to 212.2) | 9.3 (5.2 to 16.9) | 14.3 (0.9 to 31.3) |
| Viet Nam | 1392.3 (1197.5 to 1618.3) | 15.3 (9.8 to 20.9) | 123.6 (106.6 to 143.8) | 9.9 (4.4 to 15.6) | 0.1 (0.1 to 0.2) | 84.4 (15.1 to 207.5) | 9.7 (5.8 to 16.4) | 15.5 (0.3 to 39.3) |
| **South Asia** | **873.4 (754.9 to 1014.1)** | **8.1 (7.0 to 9.3)** | **84.6 (72.9 to 98.2)** | **5.9 (4.8 to 7.0)** | **0.3 (0.2 to 0.4)** | **95.5 (42.1 to 189.4)** | **8.6 (5.6 to 13.4)** | **24.8 (8.6 to 56.8)** |
| Afghanistan | 892.4 (771.9 to 1039.8) | 32.5 (27.5 to 37.6) | 91.7 (79.0 to 106.5) | 31.7 (26.4 to 37.2) | 0.0 (0.0 to 0.0) | 1142.1 (723.4 to 2345.8) | 5.1 (2.6 to 9.8) | 28.0 (19.5 to 41.9) |
| Bangladesh | 779.9 (677.2 to 910.1) | 5.8 (1.2 to 10.3) | 77.5 (66.8 to 90.6) | 4.9 (0.7 to 9.6) | 0.3 (0.2 to 0.6) | 106.7 (26.5 to 346.4) | 8.9 (5.5 to 13.9) | 28.5 (2.2 to 76.3) |
| Bhutan | 762.4 (659.1 to 888.1) | 8.6 (4.2 to 13.3) | 73.8 (64.1 to 85.6) | 6.9 (2.5 to 11.0) | 0.3 (0.1 to 0.4) | 164.1 (73.3 to 449.0) | 7.6 (4.3 to 11.9) | 35.4 (12.9 to 71.8) |
| India | 881.2 (761.2 to 1023.7) | 8.7 (7.5 to 10.0) | 85.0 (73.2 to 98.6) | 6.0 (4.9 to 7.1) | 0.2 (0.2 to 0.4) | 101.7 (43.7 to 184.4) | 8.5 (5.5 to 13.1) | 25.3 (8.3 to 58.4) |
| Iran | 984.0 (850.6 to 1143.2) | 17.3 (15.8 to 19.0) | 92.1 (79.4 to 106.4) | 16.1 (14.6 to 17.4) | 0.1 (0.1 to 0.1) | 264.3 (125.8 to 548.7) | 6.4 (3.9 to 10.5) | 30.0 (15.1 to 60.5) |
| Maldives | 1309.3 (1131.1 to 1519.6) | 8.7 (4.3 to 13.1) | 113.7 (98.8 to 133.1) | 4.3 (-0.2 to 9.0) | 0.1 (0.1 to 0.1) | 171.1 (52.5 to 358.6) | 7.6 (4.1 to 14.0) | 0.7 (-8.5 to 15.1) |
| Nepal | 847.9 (726.5 to 990.0) | 6.9 (2.6 to 10.9) | 84.8 (72.6 to 98.9) | 6.4 (2.3 to 10.3) | 0.3 (0.1 to 0.5) | 112.5 (36.7 to 273.8) | 8.6 (5.0 to 13.4) | 27.2 (7.0 to 61.9) |
| Pakistan | 913.4 (796.1 to 1059.9) | 7.1 (4.3 to 10.5) | 89.1 (76.8 to 104.1) | 7.0 (4.0 to 10.2) | 0.3 (0.2 to 0.6) | 88.9 (29.1 to 181.6) | 9.9 (5.9 to 15.8) | 27.6 (7.3 to 65.3) |
| Sri Lanka | 1431.1 (1220.6 to 1656.2) | 17.6 (12.9 to 22.7) | 124.0 (106.5 to 144.7) | 12.0 (7.5 to 16.6) | 0.1 (0.1 to 0.1) | 28.8 (-18.5 to 106.0) | 8.6 (4.9 to 15.5) | 5.8 (-5.8 to 19.1) |
| Armenia | 1057.9 (911.4 to 1240.1) | 10.5 (5.4 to 15.4) | 99.2 (84.5 to 115.4) | 8.6 (3.9 to 13.5) | 2.4 (2.0 to 2.8) | 1.3 (-28.2 to 38.3) | 43.2 (35.5 to 52.0) | -4.5 (-28.9 to 26.0) |
| Azerbaijan | 1000.8 (867.1 to 1168.0) | 14.3 (9.8 to 19.0) | 93.1 (80.5 to 108.3) | 12.2 (7.2 to 17.1) | 0.4 (0.2 to 0.7) | 60.4 (-28.1 to 223.2) | 11.4 (6.3 to 19.3) | 30.2 (-13.8 to 111.5) |
| Bahrain | 1231.3 (1064.4 to 1436.0) | 17.7 (13.0 to 23.1) | 108.4 (93.6 to 126.5) | 16.3 (11.7 to 22.2) | 1.4 (1.0 to 1.8) | 219.6 (109.7 to 400.1) | 26.4 (19.4 to 35.8) | 103.6 (44.9 to 201.5) |
| Cyprus | 2052.4 (1768.4 to 2380.4) | -17.3 (-20.5 to -13.9) | 171.4 (147.6 to 198.0) | -17.7 (-21.0 to -13.7) | 1.7 (1.3 to 2.2) | -25.3 (-54.6 to 17.6) | 27.8 (21.1 to 36.6) | -30.1 (-51.5 to -6.5) |
| Georgia | 1091.0 (950.3 to 1272.1) | 12.0 (7.0 to 18.0) | 101.5 (86.8 to 117.1) | 12.0 (7.1 to 17.0) | 0.9 (0.7 to 1.0) | 5426.3 (4132.4 to 7388.8) | 21.1 (16.8 to 27.0) | 311.3 (168.3 to 608.5) |
| Iraq | 1225.6 (1055.8 to 1427.5) | 19.5 (14.5 to 24.7) | 113.3 (97.8 to 132.2) | 17.3 (11.9 to 23.0) | 0.1 (0.1 to 0.2) | 72.3 (3.2 to 251.6) | 8.2 (5.2 to 13.5) | 16.1 (-0.2 to 48.2) |
| Israel | 1893.1 (1639.7 to 2200.6) | -19.2 (-22.5 to -15.7) | 160.0 (137.5 to 187.3) | -19.4 (-22.8 to -15.5) | 1.4 (1.2 to 1.6) | -18.1 (-27.4 to -8.1) | 28.0 (22.8 to 35.2) | -22.9 (-29.6 to -15.7) |
| Jordan | 1123.9 (970.3 to 1304.3) | 11.0 (6.9 to 16.2) | 106.2 (91.6 to 123.4) | 10.2 (5.7 to 15.1) | 0.0 (0.0 to 0.1) | 277.8 (151.3 to 450.0) | 5.8 (3.1 to 10.7) | 7.0 (-1.8 to 22.6) |
| Kuwait | 1117.7 (962.5 to 1287.9) | 13.6 (8.9 to 18.5) | 98.6 (85.1 to 115.2) | 14.5 (9.5 to 19.7) | 0.2 (0.1 to 0.2) | 81.2 (38.9 to 121.7) | 7.3 (4.8 to 11.7) | 13.8 (2.9 to 30.0) |
| Lebanon | 1293.5 (1117.1 to 1498.4) | 34.6 (28.4 to 40.9) | 118.7 (101.9 to 137.4) | 31.8 (25.8 to 37.9) | 0.4 (0.3 to 0.5) | 5.0 (-47.1 to 172.8) | 12.1 (8.6 to 17.6) | 0.4 (-38.4 to 67.2) |
| Oman | 942.8 (811.2 to 1096.2) | 25.9 (21.1 to 32.0) | 86.1 (74.1 to 99.8) | 23.7 (19.0 to 29.7) | 0.1 (0.0 to 0.1) | 126.8 (-9.5 to 486.1) | 5.3 (2.8 to 9.1) | 20.3 (0.7 to 53.4) |
| Palestine | 1096.0 (952.2 to 1288.8) | 22.5 (17.6 to 27.9) | 105.9 (91.1 to 123.2) | 19.8 (15.0 to 25.1) | 0.0 (0.0 to 0.0) | 16.4 (-28.5 to 116.0) | 5.5 (2.9 to 10.6) | 10.1 (0.7 to 20.9) |
| Qatar | 1086.5 (934.2 to 1257.2) | 21.4 (16.2 to 26.5) | 92.7 (79.9 to 107.4) | 19.0 (13.5 to 24.6) | 0.5 (0.3 to 0.8) | 88.0 (8.4 to 246.1) | 12.8 (8.3 to 19.5) | 39.6 (-2.0 to 120.4) |
| Saudi Arabia | 1030.2 (891.3 to 1193.4) | 27.5 (22.5 to 32.9) | 93.0 (80.2 to 108.7) | 26.0 (21.1 to 30.9) | 0.1 (0.0 to 0.1) | 76.5 (0.6 to 190.5) | 5.6 (3.1 to 9.8) | 16.2 (5.7 to 30.6) |
| Syrian Arab Republic | 1023.5 (880.3 to 1197.6) | 12.7 (7.9 to 17.3) | 100.3 (86.5 to 117.7) | 12.9 (8.3 to 17.5) | 0.0 (0.0 to 0.1) | 580.6 (316.6 to 1200.8) | 5.4 (2.8 to 9.9) | 9.8 (-0.8 to 27.4) |
| Turkey | 1072.0 (927.5 to 1242.8) | 6.7 (2.4 to 11.9) | 97.8 (83.6 to 113.7) | 4.9 (0.6 to 9.9) | 0.8 (0.6 to 1.1) | 24.8 (-21.5 to 106.0) | 19.1 (14.7 to 24.7) | 3.6 (-26.2 to 48.6) |
| United Arab Emirates | 1042.7 (900.8 to 1211.1) | 8.4 (3.7 to 12.9) | 93.3 (80.4 to 107.8) | 14.4 (8.9 to 19.2) | 1.3 (0.9 to 1.8) | 32.1 (-17.4 to 134.7) | 27.1 (20.4 to 34.6) | 13.6 (-23.1 to 74.5) |
| Yemen | 934.4 (805.5 to 1087.8) | 15.4 (10.4 to 20.7) | 94.0 (81.1 to 109.7) | 16.4 (11.3 to 21.7) | 0.0 (0.0 to 0.0) | 950.7 (517.5 to 2691.3) | 5.3 (2.8 to 9.9) | 15.5 (6.1 to 31.2) |

ASR: Age-standardized rate; UI: Uncertainty interval; DALYs: Disability-adjusted life years

| **Table S15. Endocarditis burden by country.** | | | | | | | | |
| --- | --- | --- | --- | --- | --- | --- | --- | --- |
|  | **Prevalence (95%UI)** | | **Incidence (95%UI)** | | **Death (95%UI)** | | **DALYs (95%UI)** | |
| **Location** | **ASR (per 100,000) 2021** | **Percent change, 1990-2021** | **ASR (per 100,000) 2021** | **Percent change, 1990-2021** | **ASR (per 100,000) 2021** | **Percent change, 1990-2021** | **ASR (per 100,000) 2021** | **Percent change, 1990-2021** |
| **Central Asia** | **0.9 (0.8 to 1.1)** | **-6.9 (-12.0 to -1.2)** | **5.3 (4.4 to 6.4)** | **17.5 (10.8 to 25.9)** | **0.2 (0.2 to 0.2)** | **-11.0 (-24.5 to 5.2)** | **6.4 (5.6 to 7.3)** | **-26.5 (-39.4 to -11.8)** |
| Kazakhstan | 1.1 (1.0 to 1.3) | -13.4 (-21.2 to -3.8) | 6.4 (5.4 to 7.6) | 30.5 (21.4 to 41.8) | 0.3 (0.3 to 0.4) | 3.2 (-21.5 to 30.2) | 11.4 (8.9 to 13.8) | -16.4 (-37.2 to 5.8) |
| Kyrgyzstan | 0.9 (0.7 to 1.0) | 21.6 (13.2 to 31.1) | 4.7 (3.9 to 5.7) | 13.8 (6.8 to 22.4) | 0.1 (0.1 to 0.1) | 9.6 (-13.1 to 38.2) | 4.8 (3.9 to 5.7) | -9.3 (-27.5 to 13.3) |
| Tajikistan | 0.6 (0.5 to 0.7) | 26.5 (17.2 to 37.1) | 4.3 (3.5 to 5.3) | 3.6 (-3.0 to 9.9) | 0.0 (0.0 to 0.0) | -10.7 (-48.7 to 38.8) | 1.3 (0.9 to 1.9) | -3.4 (-44.7 to 51.3) |
| Turkmenistan | 0.9 (0.8 to 1.0) | 37.7 (27.6 to 48.6) | 4.8 (4.0 to 5.8) | 18.4 (9.8 to 29.5) | 0.3 (0.2 to 0.4) | 52.4 (10.9 to 108.6) | 10.3 (7.4 to 15.3) | 40.4 (0.1 to 95.4) |
| Uzbekistan | 1.0 (0.8 to 1.2) | -16.7 (-23.8 to -9.4) | 5.2 (4.3 to 6.2) | 27.7 (18.0 to 38.9) | 0.1 (0.1 to 0.2) | -32.0 (-49.8 to -6.8) | 5.7 (4.6 to 7.0) | -41.9 (-57.3 to -17.4) |
| **East Asia** | **2.2 (1.9 to 2.4)** | **34.8 (27.0 to 44.0)** | **14.5 (12.2 to 17.1)** | **18.6 (6.9 to 32.6)** | **0.2 (0.1 to 0.2)** | **-58.4 (-71.2 to -25.9)** | **4.6 (3.7 to 6.2)** | **-71.4 (-82.1 to -41.0)** |
| China | 1.9 (1.6 to 2.1) | 18.4 (10.8 to 27.2) | 14.4 (12.0 to 16.9) | 16.5 (4.8 to 30.6) | 0.1 (0.1 to 0.1) | -72.8 (-82.8 to -48.4) | 3.5 (2.6 to 5.0) | -78.4 (-87.5 to -52.2) |
| Japan | 6.3 (5.2 to 7.3) | 174.8 (150.6 to 201.1) | 12.6 (10.6 to 14.8) | 36.8 (25.8 to 50.5) | 0.8 (0.7 to 0.9) | 40.7 (26.9 to 49.5) | 15.1 (13.5 to 16.2) | 19.5 (11.8 to 25.6) |
| Mongolia | 1.0 (0.9 to 1.1) | 12.6 (4.6 to 21.8) | 4.7 (3.8 to 5.6) | 10.7 (4.4 to 18.1) | 0.2 (0.2 to 0.3) | -33.1 (-54.9 to 5.2) | 7.9 (5.9 to 10.5) | -35.2 (-58.1 to 1.2) |
| North Korea | 1.9 (1.6 to 2.1) | 22.5 (16.1 to 29.8) | 10.1 (8.6 to 11.8) | 23.9 (16.5 to 31.5) | 0.4 (0.2 to 0.7) | -22.1 (-51.6 to 19.4) | 12.3 (6.8 to 23.7) | -29.4 (-60.8 to 24.6) |
| South Korea | 8.2 (7.1 to 9.3) | 36.9 (22.2 to 53.8) | 13.1 (11.1 to 15.4) | -10.4 (-18.2 to -2.2) | 0.9 (0.6 to 1.3) | -66.9 (-80.0 to -33.5) | 16.1 (12.5 to 24.1) | -66.8 (-78.9 to -32.3) |
| Taiwan [Province of China] | 16.2 (13.7 to 18.9) | 600.3 (531.7 to 678.0) | 23.9 (20.6 to 27.5) | 119.4 (95.1 to 146.3) | 2.2 (1.9 to 2.4) | 416.7 (365.6 to 464.9) | 50.4 (45.9 to 54.1) | 325.4 (287.6 to 359.3) |
| **Southeast Asia** | **5.5 (4.8 to 6.2)** | **33.5 (27.0 to 41.6)** | **17.7 (15.5 to 20.3)** | **28.3 (22.6 to 34.4)** | **1.2 (1.0 to 1.8)** | **-20.6 (-32.4 to -6.6)** | **37.0 (29.3 to 52.8)** | **-24.9 (-34.8 to -13.4)** |
| Brunei Darussalam | 3.6 (3.1 to 4.2) | 45.7 (35.2 to 59.2) | 14.2 (12.3 to 16.3) | 33.5 (24.1 to 43.6) | 1.6 (1.2 to 2.0) | -20.7 (-40.7 to 6.0) | 34.1 (27.9 to 44.0) | -21.1 (-42.2 to 5.4) |
| Cambodia | 2.2 (1.9 to 2.5) | 32.8 (23.4 to 42.6) | 11.4 (9.9 to 13.1) | 38.6 (26.7 to 52.7) | 0.9 (0.5 to 1.8) | -21.7 (-44.0 to 4.3) | 28.7 (16.7 to 52.2) | -23.0 (-47.1 to 2.8) |
| Indonesia | 2.8 (2.4 to 3.2) | 25.5 (18.2 to 32.8) | 11.9 (10.1 to 13.8) | 19.4 (14.0 to 25.5) | 0.9 (0.5 to 1.6) | -17.9 (-34.9 to -0.1) | 28.1 (17.4 to 50.0) | -21.1 (-37.5 to -3.3) |
| Lao PDR | 2.4 (2.0 to 2.7) | 55.6 (42.9 to 70.1) | 10.2 (8.8 to 11.9) | 25.4 (15.9 to 35.3) | 1.2 (0.6 to 2.1) | -24.3 (-47.7 to 8.1) | 42.9 (23.4 to 74.6) | -16.6 (-43.9 to 18.5) |
| Malaysia | 7.7 (6.6 to 8.9) | 34.5 (26.2 to 44.4) | 20.0 (17.6 to 23.0) | 13.1 (5.3 to 21.2) | 1.5 (1.1 to 1.8) | -22.6 (-35.6 to -3.0) | 47.1 (35.7 to 57.0) | -26.9 (-38.5 to -10.7) |
| Myanmar | 2.3 (2.0 to 2.6) | 33.5 (25.2 to 43.0) | 11.9 (10.3 to 13.7) | 30.6 (22.2 to 40.6) | 1.0 (0.6 to 1.9) | -33.2 (-52.6 to -7.3) | 37.2 (22.7 to 64.9) | -33.4 (-54.1 to -4.2) |
| Philippines | 2.6 (2.3 to 3.0) | 34.5 (27.7 to 41.7) | 14.1 (12.2 to 16.3) | 40.4 (31.5 to 50.2) | 0.9 (0.7 to 1.1) | -11.1 (-26.4 to 8.1) | 32.1 (22.1 to 39.0) | -6.6 (-21.6 to 10.9) |
| Singapore | 5.1 (4.5 to 5.8) | 213.5 (191.9 to 236.4) | 17.5 (16.9 to 20.3) | 137.6 (116.9 to 159.8) | 0.5 (0.5 to 0.6) | 0.9 (-8.9 to 9.7) | 11.0 (10.0 to 11.8) | -14.1 (-21.4 to -7.1) |
| Thailand | 19.4 (16.6 to 22.2) | 28.8 (20.7 to 38.7) | 33.5 (29.8 to 37.9) | 10.0 (1.7 to 19.0) | 2.5 (1.9 to 3.4) | -32.5 (-48.6 to -10.8) | 77.8 (59.1 to 105.6) | -31.1 (-47.9 to -9.3) |
| Timor-Leste | 3.4 (2.8 to 4.0) | 43.3 (28.7 to 61.1) | 11.0 (9.4 to 12.8) | 20.3 (10.8 to 30.5) | 1.5 (0.8 to 2.9) | 13.6 (-21.2 to 62.6) | 58.0 (30.6 to 106.6) | 34.7 (-11.1 to 92.0) |
| Viet Nam | 4.0 (3.5 to 4.5) | 29.9 (23.0 to 38.4) | 18.9 (16.3 to 21.8) | 44.6 (34.3 to 56.7) | 0.8 (0.5 to 1.6) | -28.1 (-49.5 to -1.2) | 24.7 (14.3 to 45.8) | -31.1 (-51.8 to -4.9) |
| **South Asia** | **2.7 (2.3 to 3.1)** | **52.4 (45.1 to 60.7)** | **5.5 (4.6 to 6.6)** | **38.9 (33.3 to 44.6)** | **0.8 (0.6 to 1.0)** | **-14.7 (-28.3 to 4.6)** | **24.2 (18.9 to 28.9)** | **-17.6 (-30.0 to -3.5)** |
| Afghanistan | 1.5 (1.3 to 1.8) | 37.7 (27.0 to 50.1) | 5.3 (4.5 to 6.3) | 7.1 (1.3 to 13.8) | 0.8 (0.6 to 1.2) | -7.1 (-32.7 to 43.8) | 27.0 (19.7 to 37.2) | -9.1 (-32.2 to 28.4) |
| Bangladesh | 3.0 (2.6 to 3.5) | 50.9 (36.4 to 64.7) | 6.1 (5.1 to 7.2) | 48.6 (39.0 to 60.1) | 1.0 (0.7 to 1.4) | -22.8 (-44.9 to 6.9) | 28.1 (20.2 to 36.6) | -25.9 (-46.7 to 5.2) |
| Bhutan | 2.6 (2.2 to 2.9) | 30.3 (19.5 to 41.5) | 5.8 (4.9 to 6.9) | 57.2 (44.4 to 70.0) | 0.7 (0.5 to 1.0) | -30.7 (-50.8 to -3.7) | 20.3 (13.9 to 28.4) | -32.9 (-52.8 to -3.9) |
| India | 2.7 (2.3 to 3.1) | 59.5 (51.4 to 68.5) | 5.5 (4.6 to 6.5) | 39.8 (33.8 to 45.9) | 0.8 (0.6 to 0.9) | -14.1 (-29.0 to 7.4) | 22.8 (17.7 to 27.6) | -19.3 (-32.5 to -3.9) |
| Iran | 2.8 (2.4 to 3.2) | 46.6 (40.2 to 54.3) | 9.9 (8.4 to 11.7) | 23.4 (19.5 to 27.4) | 0.3 (0.3 to 0.4) | -29.5 (-47.1 to -2.5) | 9.3 (7.6 to 11.7) | -44.1 (-55.2 to -27.8) |
| Maldives | 3.3 (2.9 to 3.7) | 61.4 (50.2 to 73.7) | 14.5 (12.4 to 16.8) | 27.0 (19.0 to 35.7) | 0.4 (0.3 to 0.5) | -47.1 (-62.9 to -27.1) | 12.2 (8.9 to 17.9) | -44.7 (-62.8 to -17.5) |
| Nepal | 2.6 (2.2 to 3.0) | 53.9 (39.7 to 69.2) | 5.2 (4.4 to 6.1) | 46.1 (35.9 to 57.1) | 0.9 (0.7 to 1.2) | -9.4 (-33.9 to 25.1) | 26.5 (18.9 to 34.7) | -12.5 (-37.3 to 18.2) |
| Pakistan | 2.3 (2.0 to 2.7) | 15.8 (8.1 to 24.9) | 5.2 (4.4 to 6.1) | 23.3 (18.0 to 29.1) | 0.9 (0.7 to 1.2) | -5.5 (-28.0 to 24.3) | 29.0 (21.1 to 38.0) | -2.1 (-24.3 to 25.0) |
| Sri Lanka | 5.9 (5.1 to 6.6) | 70.0 (60.7 to 79.3) | 25.1 (21.8 to 28.9) | 49.9 (39.1 to 62.5) | 1.1 (0.7 to 1.5) | -19.4 (-47.8 to 20.7) | 32.9 (22.3 to 46.7) | -20.9 (-48.0 to 19.1) |
| Armenia | 1.0 (0.8 to 1.1) | 27.6 (16.7 to 40.1) | 5.2 (4.3 to 6.3) | 18.3 (10.2 to 27.0) | 0.1 (0.1 to 0.1) | 2.2 (-23.4 to 34.8) | 3.1 (2.6 to 3.7) | -18.3 (-38.4 to 7.8) |
| Azerbaijan | 0.5 (0.4 to 0.5) | 11.4 (5.5 to 17.1) | 4.7 (3.8 to 5.7) | 10.5 (4.8 to 17.5) | 0.0 (0.0 to 0.0) | -40.4 (-65.9 to -6.2) | 1.1 (0.7 to 1.6) | -44.4 (-68.7 to -10.9) |
| Bahrain | 3.5 (3.1 to 4.0) | 59.2 (48.1 to 71.4) | 8.7 (7.3 to 10.5) | 15.5 (9.4 to 21.6) | 0.6 (0.4 to 0.8) | -22.8 (-46.3 to 5.8) | 15.2 (10.7 to 18.9) | -32.0 (-52.1 to -5.1) |
| Cyprus | 6.9 (5.9 to 8.0) | 56.6 (45.0 to 70.3) | 17.3 (14.9 to 20.1) | 20.5 (11.2 to 30.0) | 1.6 (1.1 to 2.1) | -55.0 (-66.9 to -34.9) | 27.1 (20.1 to 33.4) | -53.7 (-64.4 to -37.6) |
| Georgia | 1.6 (1.4 to 1.9) | 27.2 (16.9 to 40.4) | 6.7 (5.6 to 7.8) | 15.7 (5.0 to 27.8) | 0.3 (0.2 to 0.3) | 56.6 (18.2 to 105.3) | 9.6 (8.0 to 11.3) | 24.3 (-3.6 to 62.1) |
| Iraq | 2.0 (1.7 to 2.2) | 39.0 (30.5 to 49.2) | 8.4 (7.2 to 10.0) | 31.8 (25.5 to 38.8) | 0.4 (0.3 to 0.6) | -18.8 (-41.9 to 20.9) | 12.1 (8.8 to 16.6) | -30.3 (-48.7 to -3.4) |
| Israel | 6.7 (5.8 to 7.8) | 295.5 (256.2 to 342.0) | 14.6 (12.5 to 17.0) | 31.5 (23.4 to 39.7) | 0.9 (0.8 to 0.9) | 114.0 (93.7 to 134.4) | 16.7 (15.2 to 18.1) | 104.5 (87.0 to 124.4) |
| Jordan | 7.0 (6.0 to 8.3) | 19.6 (11.6 to 29.3) | 11.6 (10.0 to 13.6) | 6.0 (-0.5 to 13.0) | 0.9 (0.6 to 1.2) | -50.2 (-63.0 to -33.2) | 27.7 (21.7 to 36.7) | -54.3 (-66.1 to -37.4) |
| Kuwait | 3.3 (2.9 to 3.8) | 109.9 (94.7 to 125.1) | 10.5 (8.8 to 12.5) | 32.1 (23.8 to 41.0) | 0.3 (0.2 to 0.3) | -5.9 (-22.8 to 15.2) | 8.5 (7.3 to 9.9) | -18.1 (-33.0 to 1.2) |
| Lebanon | 5.7 (5.0 to 6.6) | 46.0 (35.1 to 55.8) | 9.6 (8.2 to 11.4) | 24.3 (17.8 to 31.7) | 0.6 (0.4 to 0.8) | -60.3 (-72.2 to -45.3) | 15.7 (12.6 to 20.0) | -61.6 (-72.9 to -46.0) |
| Oman | 1.7 (1.5 to 2.0) | 45.7 (36.1 to 56.0) | 9.6 (8.2 to 11.3) | 26.2 (19.9 to 33.5) | 0.1 (0.1 to 0.2) | -53.2 (-69.6 to -27.7) | 4.2 (3.1 to 5.4) | -55.2 (-71.5 to -31.2) |
| Palestine | 1.2 (1.0 to 1.3) | 25.9 (18.8 to 33.3) | 6.6 (5.4 to 8.1) | 16.2 (10.0 to 21.7) | 0.1 (0.1 to 0.2) | -35.0 (-52.9 to -11.0) | 4.5 (3.6 to 6.0) | -40.3 (-57.4 to -21.7) |
| Qatar | 3.8 (3.3 to 4.3) | 89.6 (76.5 to 102.9) | 8.8 (7.4 to 10.5) | 30.4 (23.8 to 38.6) | 0.3 (0.2 to 0.4) | -51.0 (-72.5 to -16.5) | 7.8 (5.3 to 10.7) | -48.6 (-68.6 to -16.7) |
| Saudi Arabia | 2.3 (2.0 to 2.6) | 58.0 (45.5 to 72.0) | 9.6 (8.2 to 11.3) | 43.4 (32.5 to 54.7) | 0.4 (0.3 to 0.6) | -19.3 (-57.2 to 45.3) | 12.5 (7.8 to 16.8) | -27.6 (-60.0 to 21.5) |
| Syrian Arab Republic | 1.1 (0.9 to 1.2) | 28.5 (21.7 to 35.4) | 7.0 (5.8 to 8.4) | 22.2 (16.2 to 28.4) | 0.2 (0.1 to 0.3) | -30.6 (-56.1 to 1.8) | 5.4 (3.8 to 7.3) | -42.6 (-62.6 to -12.1) |
| Turkey | 5.9 (5.0 to 6.7) | 36.2 (26.2 to 47.1) | 11.2 (9.5 to 13.2) | 33.8 (24.2 to 43.2) | 0.8 (0.7 to 1.2) | -49.3 (-64.4 to -31.2) | 23.3 (18.5 to 30.8) | -57.6 (-71.0 to -40.0) |
| United Arab Emirates | 3.8 (3.3 to 4.3) | 34.2 (22.9 to 44.5) | 8.6 (7.2 to 10.3) | 16.3 (9.9 to 23.7) | 0.8 (0.6 to 1.0) | -26.8 (-48.7 to -1.5) | 19.1 (14.7 to 25.4) | -37.9 (-57.0 to -15.7) |
| Yemen | 2.1 (1.8 to 2.4) | 54.6 (43.1 to 67.2) | 6.1 (5.3 to 7.3) | 15.7 (9.7 to 22.0) | 0.8 (0.6 to 1.3) | -2.0 (-34.3 to 47.2) | 25.2 (17.6 to 35.9) | -11.3 (-36.8 to 27.4) |

ASR: Age-standardized rate; UI: Uncertainty interval; DALYs: Disability-adjusted life years

| **Table S16. Other cardiovascular and circulatory diseases by country.** | | | | | | |
| --- | --- | --- | --- | --- | --- | --- |
|  | **Prevalence (95%UI)** | | **Death (95%UI)** | | **DALYs (95%UI)** | |
| **Location** | **ASR (per 100,000) 2021** | **Percent change, 1990-2021** | **ASR (per 100,000) 2021** | **Percent change, 1990-2021** | **ASR (per 100,000) 2021** | **Percent change, 1990-2021** |
| **Central Asia** | **496.9 (375.2 to 645.6)** | **32.0 (18.2 to 46.1)** | **2.8 (2.5 to 3.1)** | **36.8 (14.6 to 60.1)** | **95.2 (81.8 to 111.3)** | **18.0 (3.5 to 32.0)** |
| Kazakhstan | 440.7 (323.7 to 596.7) | -1.7 (-16.6 to 15.2) | 3.0 (2.6 to 3.5) | 19.9 (-13.9 to 58.2) | 97.0 (81.4 to 113.2) | 0.9 (-24.3 to 25.3) |
| Kyrgyzstan | 462.9 (353.6 to 591.5) | -8.7 (-18.1 to 2.0) | 2.1 (1.8 to 2.5) | -12.0 (-29.8 to 8.3) | 82.9 (69.5 to 98.0) | -19.6 (-31.8 to -6.0) |
| Tajikistan | 151.8 (117.9 to 194.0) | 7.6 (-3.2 to 21.4) | 0.5 (0.4 to 0.7) | -28.2 (-53.5 to 9.0) | 23.7 (18.1 to 30.8) | -21.2 (-41.6 to 11.3) |
| Turkmenistan | 329.7 (250.9 to 430.0) | -16.7 (-25.7 to -6.7) | 2.4 (1.8 to 3.2) | -20.4 (-41.9 to 6.9) | 90.9 (71.8 to 115.4) | -22.2 (-39.1 to 0.1) |
| Uzbekistan | 575.8 (431.1 to 754.2) | 122.2 (86.0 to 162.5) | 3.2 (2.6 to 3.8) | 190.6 (114.4 to 318.8) | 110.0 (91.2 to 132.9) | 120.9 (78.7 to 169.6) |
| **East Asia** | **299.1 (225.5 to 395.1)** | **31.3 (18.7 to 44.4)** | **0.7 (0.5 to 0.8)** | **-44.7 (-60.3 to -24.6)** | **31.0 (24.3 to 39.3)** | **-38.3 (-49.8 to -19.1)** |
| China | 293.3 (220.5 to 388.3) | 33.2 (19.8 to 46.9) | 0.7 (0.5 to 0.8) | -44.8 (-60.9 to -24.0) | 30.4 (23.8 to 38.8) | -38.8 (-50.5 to -19.1) |
| Japan | 1010.7 (774.9 to 1284.4) | 64.4 (43.9 to 86.0) | 1.1 (0.9 to 1.2) | -40.8 (-43.0 to -38.4) | 77.3 (57.0 to 104.2) | 13.9 (0.4 to 27.9) |
| Mongolia | 399.0 (290.6 to 543.3) | 12.8 (-3.9 to 32.2) | 1.9 (1.4 to 2.6) | -28.7 (-51.1 to 3.4) | 72.0 (56.2 to 91.7) | -22.5 (-42.2 to 4.4) |
| North Korea | 219.1 (171.8 to 288.1) | 1.0 (-9.2 to 13.1) | 0.9 (0.6 to 1.4) | -18.4 (-40.2 to 17.7) | 37.0 (27.7 to 49.2) | -20.4 (-39.3 to 8.7) |
| South Korea | 845.5 (655.8 to 1081.5) | 21.8 (4.6 to 42.5) | 0.7 (0.6 to 0.9) | -69.8 (-76.0 to -46.8) | 58.0 (42.3 to 78.9) | -35.8 (-48.4 to -18.1) |
| Taiwan [Province of China] | 648.1 (507.3 to 837.2) | 3.1 (-6.5 to 14.4) | 0.8 (0.7 to 0.8) | -54.3 (-58.4 to -50.3) | 51.6 (39.5 to 67.6) | -32.0 (-39.2 to -23.4) |
| **Southeast Asia** | **388.4 (310.0 to 483.7)** | **26.6 (20.1 to 33.4)** | **1.3 (1.1 to 1.9)** | **-11.2 (-27.4 to 3.7)** | **59.9 (49.6 to 77.8)** | **-6.4 (-17.9 to 9.6)** |
| Brunei Darussalam | 459.5 (328.6 to 632.9) | 34.6 (16.7 to 56.4) | 2.2 (1.8 to 2.6) | -16.0 (-33.2 to 7.6) | 74.5 (62.3 to 89.3) | -10.6 (-25.6 to 8.4) |
| Cambodia | 247.2 (193.3 to 319.8) | 22.9 (11.4 to 35.8) | 1.2 (0.9 to 2.1) | -14.5 (-37.2 to 15.5) | 48.8 (36.7 to 70.6) | -15.0 (-34.1 to 17.7) |
| Indonesia | 303.6 (235.0 to 391.5) | 7.0 (-1.2 to 16.0) | 1.4 (1.0 to 2.4) | -2.5 (-25.0 to 19.9) | 57.6 (44.6 to 84.7) | -9.1 (-25.3 to 10.6) |
| Lao PDR | 238.8 (188.9 to 304.3) | 22.4 (10.0 to 35.9) | 1.5 (1.0 to 2.7) | -29.1 (-49.3 to 5.1) | 63.2 (44.7 to 99.3) | -26.2 (-45.9 to 13.3) |
| Malaysia | 887.5 (706.9 to 1108.4) | 42.0 (28.8 to 55.1) | 2.5 (2.1 to 2.8) | -2.8 (-27.1 to 20.3) | 110.6 (92.5 to 132.6) | 4.3 (-11.6 to 19.6) |
| Myanmar | 284.3 (224.6 to 359.3) | 31.5 (18.5 to 47.2) | 1.6 (1.1 to 2.8) | -24.3 (-43.8 to 4.4) | 70.1 (51.8 to 108.3) | -21.5 (-40.7 to 12.2) |
| Philippines | 225.8 (173.2 to 296.0) | 11.8 (2.4 to 20.7) | 1.1 (1.0 to 1.3) | -4.3 (-19.6 to 12.0) | 51.9 (44.1 to 61.5) | -5.7 (-17.9 to 8.5) |
| Singapore | 494.7 (379.8 to 638.3) | 40.7 (20.2 to 68.1) | 0.6 (0.5 to 0.6) | -66.2 (-69.5 to -63.2) | 38.3 (28.8 to 50.9) | -37.0 (-47.7 to -23.9) |
| Thailand | 624.6 (486.7 to 786.7) | 37.9 (26.5 to 50.2) | 0.8 (0.6 to 1.0) | -21.4 (-40.3 to 6.0) | 58.0 (45.5 to 74.1) | 1.4 (-14.7 to 20.9) |
| Timor-Leste | 244.5 (192.9 to 309.7) | -2.9 (-12.5 to 7.9) | 1.3 (0.9 to 2.2) | -15.9 (-41.7 to 19.8) | 56.2 (41.6 to 81.4) | -16.5 (-35.2 to 18.7) |
| Viet Nam | 388.3 (310.6 to 496.1) | 24.9 (12.2 to 37.9) | 1.1 (0.7 to 2.0) | -8.8 (-41.1 to 20.0) | 49.4 (36.4 to 70.7) | -2.9 (-23.3 to 17.6) |
| **South Asia** | **684.0 (512.9 to 908.1)** | **13.8 (5.5 to 22.8)** | **2.9 (2.3 to 3.7)** | **-6.9 (-35.0 to 17.9)** | **105.0 (85.7 to 129.0)** | **-5.8 (-22.8 to 11.5)** |
| Afghanistan | 1281.6 (996.1 to 1609.2) | 33.4 (18.7 to 50.3) | 10.9 (6.0 to 16.5) | 1.7 (-33.4 to 41.3) | 356.2 (227.1 to 480.7) | -2.0 (-30.3 to 32.7) |
| Bangladesh | 905.3 (700.0 to 1163.7) | 26.2 (9.3 to 44.7) | 3.4 (2.1 to 5.6) | -15.2 (-42.0 to 22.7) | 116.9 (84.1 to 166.8) | -11.2 (-32.2 to 18.4) |
| Bhutan | 850.4 (674.0 to 1072.1) | 34.8 (21.2 to 49.4) | 3.4 (2.5 to 5.0) | -0.5 (-30.7 to 45.1) | 120.5 (91.8 to 161.5) | -0.5 (-25.6 to 33.0) |
| India | 674.6 (501.1 to 905.2) | 14.4 (4.9 to 23.8) | 2.7 (2.2 to 3.5) | -7.3 (-36.6 to 20.6) | 100.2 (81.1 to 124.9) | -7.2 (-25.3 to 10.6) |
| Iran | 2297.1 (1759.6 to 2976.4) | 52.8 (39.2 to 67.9) | 6.6 (5.8 to 8.0) | -8.5 (-34.7 to 15.4) | 262.4 (216.8 to 324.1) | -12.8 (-30.7 to 3.6) |
| Maldives | 1077.9 (850.4 to 1362.8) | 38.0 (24.6 to 53.6) | 2.2 (1.8 to 2.7) | -48.7 (-62.5 to -20.0) | 111.2 (88.5 to 139.3) | -35.4 (-51.1 to -5.0) |
| Nepal | 495.5 (383.0 to 634.7) | 8.4 (-3.7 to 22.9) | 3.2 (2.3 to 4.5) | 5.1 (-24.7 to 50.8) | 97.7 (75.4 to 128.4) | -5.4 (-26.8 to 25.8) |
| Pakistan | 567.9 (421.6 to 753.9) | -1.4 (-11.6 to 8.8) | 3.7 (2.8 to 4.9) | 10.9 (-19.0 to 45.4) | 124.4 (100.9 to 158.2) | 6.4 (-14.6 to 29.7) |
| Sri Lanka | 470.0 (372.6 to 594.1) | 67.3 (50.7 to 84.0) | 1.3 (0.9 to 1.8) | -14.0 (-42.7 to 21.8) | 59.0 (44.4 to 77.1) | -0.4 (-24.4 to 28.6) |
| Armenia | 967.0 (741.2 to 1227.5) | 102.8 (77.1 to 130.4) | 3.4 (2.8 to 3.9) | 52.1 (19.5 to 95.0) | 122.3 (100.5 to 148.0) | 52.3 (27.4 to 79.6) |
| Azerbaijan | 366.1 (271.9 to 485.5) | 15.2 (-0.2 to 35.9) | 2.0 (1.3 to 2.7) | -15.7 (-47.7 to 23.8) | 69.2 (52.0 to 91.2) | -18.4 (-40.9 to 8.4) |
| Bahrain | 1290.7 (1020.9 to 1628.2) | 60.2 (44.6 to 76.8) | 5.1 (3.6 to 6.2) | -14.7 (-40.2 to 9.6) | 157.0 (123.5 to 190.8) | -7.3 (-25.4 to 11.5) |
| Cyprus | 789.0 (594.5 to 1042.9) | 9.3 (-6.4 to 27.8) | 2.7 (2.1 to 3.3) | -65.2 (-77.4 to -51.9) | 83.6 (66.7 to 106.2) | -47.2 (-62.4 to -33.0) |
| Georgia | 740.7 (509.3 to 1021.3) | 43.1 (12.7 to 69.3) | 4.1 (3.5 to 4.7) | 59.1 (21.2 to 99.4) | 141.5 (120.7 to 167.3) | 33.7 (10.2 to 55.6) |
| Iraq | 783.3 (613.4 to 983.8) | -6.7 (-16.3 to 3.5) | 4.7 (3.3 to 6.7) | -19.3 (-50.0 to 14.8) | 150.5 (119.6 to 194.0) | -30.8 (-47.4 to -6.3) |
| Israel | 1183.2 (897.4 to 1512.6) | 106.1 (78.7 to 141.3) | 1.9 (1.6 to 2.1) | -28.0 (-34.9 to -21.9) | 92.6 (70.2 to 121.4) | 12.5 (-4.6 to 29.9) |
| Jordan | 860.7 (688.6 to 1071.3) | 5.8 (-5.3 to 18.6) | 2.3 (1.8 to 2.9) | -40.5 (-56.2 to -19.0) | 98.5 (78.9 to 120.6) | -34.6 (-47.5 to -20.7) |
| Kuwait | 651.9 (518.0 to 808.7) | 49.3 (34.1 to 65.3) | 1.2 (1.0 to 1.4) | -25.0 (-36.6 to -11.7) | 61.9 (49.0 to 78.3) | -10.2 (-22.4 to 2.3) |
| Lebanon | 2305.6 (1870.6 to 2823.8) | 2.9 (-6.3 to 12.0) | 4.2 (3.4 to 5.0) | -59.6 (-69.5 to -39.0) | 200.8 (156.8 to 255.3) | -42.5 (-54.5 to -22.5) |
| Oman | 3267.7 (2610.6 to 4016.3) | -20.1 (-28.6 to -11.3) | 16.3 (12.9 to 20.5) | -42.5 (-63.0 to -13.1) | 502.2 (408.9 to 613.6) | -46.6 (-60.1 to -28.8) |
| Palestine | 741.8 (588.5 to 916.8) | 0.9 (-9.2 to 10.8) | 3.0 (2.4 to 3.5) | -38.0 (-60.4 to -15.6) | 101.4 (85.1 to 121.1) | -35.9 (-51.1 to -21.2) |
| Qatar | 1589.7 (1244.9 to 2017.5) | 38.9 (24.6 to 55.7) | 3.4 (2.5 to 4.4) | -50.4 (-64.7 to -30.1) | 145.4 (111.3 to 188.3) | -26.2 (-42.0 to -8.2) |
| Saudi Arabia | 1239.3 (984.8 to 1533.4) | 33.3 (21.2 to 47.8) | 6.3 (4.7 to 8.0) | 6.4 (-31.8 to 65.0) | 203.2 (161.5 to 256.5) | 1.4 (-27.8 to 43.5) |
| Syrian Arab Republic | 1660.3 (1319.6 to 2089.2) | 3.0 (-7.6 to 14.8) | 9.3 (7.2 to 11.9) | -22.3 (-48.5 to 9.5) | 294.5 (234.0 to 368.8) | -32.5 (-49.0 to -8.7) |
| Turkey | 1645.9 (1301.7 to 2034.6) | 18.0 (6.5 to 31.2) | 3.8 (3.0 to 4.9) | -38.0 (-56.2 to -15.1) | 165.1 (131.5 to 209.6) | -36.2 (-48.4 to -17.2) |
| United Arab Emirates | 1345.8 (1051.7 to 1722.1) | 11.3 (-0.8 to 25.0) | 5.4 (3.5 to 7.2) | -11.9 (-33.5 to 23.9) | 167.4 (126.5 to 213.2) | -20.9 (-36.2 to -2.2) |
| Yemen | 1463.8 (1158.4 to 1803.2) | 45.2 (29.3 to 62.0) | 9.5 (4.5 to 14.2) | 10.4 (-29.7 to 62.5) | 317.2 (191.3 to 428.9) | 0.1 (-27.6 to 45.8) |

ASR: Age-standardized rate; UI: Uncertainty interval; DALYs: Disability-adjusted life years

| **Table S17. Percentage of DALYS attributable to risk factors of cardiovascular diseases in Asia, 2021** | | | | |
| --- | --- | --- | --- | --- |
| **Cause** | **Risk Factor** | **Male** | **Female** | **Both** |
| Aortic aneurysm | Dietary risks | 7.9 (5.8 to 10.5) | 7.6 (5.5 to 9.9) | 7.8 (5.7 to 10.2) |
|  | High body-mass index | 4.9 (2.7 to 7.9) | 5.3 (2.9 to 8.7) | 5 (2.7 to 8.2) |
|  | High systolic blood pressure | 16.2 (12.2 to 20.7) | 16.8 (12.4 to 21.3) | 16.4 (12.4 to 20.8) |
|  | Lead exposure | 0.9 (-0.1 to 2.3) | 0.7 (-0.1 to 1.8) | 0.8 (-0.1 to 2.2) |
|  | Tobacco | 49 (42.8 to 54.2) | 10.9 (8.6 to 13.5) | 35.5 (31 to 39.9) |
| Atrial fibrillation and flutter | Alcohol use | 5.6 (4.4 to 6.9) | 0.7 (0.5 to 0.9) | 3 (2.4 to 3.8) |
|  | Dietary risks | 5.5 (1 to 13.8) | 3.8 (0.4 to 10.8) | 4.6 (0.7 to 12.3) |
|  | High body-mass index | 3.2 (1.4 to 5.3) | 5.3 (2.3 to 8.7) | 4.3 (1.9 to 7.1) |
|  | High systolic blood pressure | 29.1 (10.3 to 46.3) | 30.5 (10.5 to 48.5) | 29.8 (10.6 to 47.7) |
|  | Lead exposure | 3.8 (-0.5 to 9.5) | 3 (-0.4 to 7.8) | 3.4 (-0.5 to 8.6) |
|  | Tobacco | 10.2 (6.3 to 13.9) | 1.5 (0.8 to 2.1) | 5.6 (3.5 to 7.8) |
| Cardiomyopathy and myocarditis | Alcohol use | 6.2 (2.5 to 10.6) | 1.3 (0.5 to 1.7) | 4.4 (2 to 7.1) |
|  | Suboptimal temperature | 7.6 (2.7 to 12.7) | 7.8 (2.7 to 12.8) | 7.7 (2.7 to 12.7) |
| Cardiovascular diseases | Air pollution | 28.8 (23.9 to 33.6) | 27.6 (23.2 to 32.3) | 28.3 (23.7 to 33) |
|  | Alcohol use | 2.9 (0.3 to 5.6) | 0.4 (0.1 to 0.7) | 1.8 (0.2 to 3.6) |
|  | Dietary risks | 35.2 (8.5 to 49.9) | 30 (7.4 to 43.9) | 33 (8 to 47.5) |
|  | High LDL cholesterol | 21 (13.7 to 28.7) | 18.4 (11.2 to 26.2) | 19.9 (12.6 to 27.7) |
|  | High body-mass index | 7.3 (3.6 to 11) | 9 (4.9 to 13.4) | 8 (4.2 to 12) |
|  | High fasting plasma glucose | 10.1 (8.8 to 11.4) | 9.7 (8.4 to 11) | 9.9 (8.7 to 11.2) |
|  | High systolic blood pressure | 50 (41.2 to 57.6) | 51.8 (42.9 to 59) | 50.7 (41.7 to 58) |
|  | Impaired kidney function | 9.7 (7.9 to 11.6) | 9.9 (8 to 11.8) | 9.8 (7.9 to 11.7) |
|  | Lead exposure | 8.6 (-1.1 to 17.9) | 7.4 (-0.9 to 15.4) | 8.1 (-1 to 16.8) |
|  | Low physical activity | 1.3 (0.6 to 2.2) | 2.4 (1 to 3.9) | 1.8 (0.8 to 2.9) |
|  | Suboptimal temperature | 6 (5 to 7.5) | 5.6 (4.7 to 7) | 5.9 (4.9 to 7.3) |
|  | Tobacco | 26.8 (23.2 to 30.4) | 8.5 (6.5 to 10.5) | 19.2 (16.1 to 22.1) |
| Hypertensive heart disease | Alcohol use | 9.6 (6.2 to 13.6) | 1 (0.5 to 1.6) | 5.1 (3.2 to 7.4) |
|  | Dietary risks | 71.2 (60.9 to 80.5) | 64.1 (54.4 to 74.4) | 67.5 (58.5 to 76.7) |
|  | High body-mass index | 41 (32.8 to 49.4) | 43 (33.3 to 53.7) | 42 (33.8 to 51.2) |
|  | Lead exposure | 29 (0 to 50.4) | 23.8 (0 to 42.9) | 26.3 (0 to 46.7) |
|  | Suboptimal temperature | 6.8 (4.9 to 8.9) | 6.4 (4.1 to 9) | 6.6 (4.4 to 8.9) |
| Ischemic heart disease | Air pollution | 35.3 (27.6 to 42.7) | 35.6 (27.8 to 43) | 35.4 (27.7 to 42.8) |
|  | Alcohol use | -2.2 (-3.4 to -0.5) | -0.3 (-0.5 to -0.1) | -1.5 (-2.3 to -0.3) |
|  | Dietary risks | 51 (-1.8 to 77.6) | 46.8 (-3.4 to 74) | 49.4 (-2.4 to 76.1) |
|  | High LDL cholesterol | 35.6 (25.4 to 46.1) | 33.6 (23.5 to 44) | 34.9 (24.7 to 45.1) |
|  | High body-mass index | 9.1 (3.6 to 14.4) | 10.6 (4.2 to 16.5) | 9.6 (3.8 to 15.1) |
|  | High fasting plasma glucose | 13.1 (11.4 to 15) | 13.6 (11.7 to 15.4) | 13.3 (11.5 to 15.1) |
|  | High systolic blood pressure | 47.6 (38.2 to 56.2) | 50.2 (40.6 to 58.8) | 48.6 (39.4 to 57.2) |
|  | Impaired kidney function | 12.9 (9.2 to 16.8) | 14.3 (10.2 to 18.4) | 13.4 (9.6 to 17.4) |
|  | Lead exposure | 7.7 (-1.1 to 16.2) | 6.6 (-0.9 to 14) | 7.3 (-1 to 15.4) |
|  | Low physical activity | 1.4 (0.6 to 2.2) | 3.4 (1.5 to 5.4) | 2.1 (1 to 3.3) |
|  | Suboptimal temperature | 6.9 (4.6 to 9.8) | 6.9 (4.7 to 9.6) | 6.9 (4.7 to 9.7) |
|  | Tobacco | 32.5 (28.3 to 36.6) | 11.4 (8.9 to 13.8) | 24.5 (20.9 to 28) |
| Lower extremity peripheral arterial disease | Dietary risks | 5.7 (2.1 to 10.8) | 6.1 (1.9 to 11.7) | 6 (2 to 11.3) |
|  | High body-mass index | 10.6 (3.1 to 25.1) | 14.1 (4 to 33.9) | 12.7 (3.6 to 30.6) |
|  | High fasting plasma glucose | 34.1 (27.7 to 40.9) | 30.5 (24.2 to 37.4) | 32 (25.6 to 38.9) |
|  | High systolic blood pressure | 13 (2.6 to 23.5) | 13.1 (2.6 to 23.9) | 13 (2.6 to 24) |
|  | Impaired kidney function | 27.4 (18.8 to 36.8) | 28.5 (18.6 to 38.3) | 28 (18.7 to 37.7) |
|  | Lead exposure | 1 (-0.2 to 2.6) | 0.7 (-0.1 to 2) | 0.9 (-0.1 to 2.2) |
|  | Low physical activity | 2.2 (0.6 to 4.4) | 3.2 (0.9 to 6.4) | 2.8 (0.8 to 5.5) |
|  | Tobacco | 46.3 (37.6 to 54.4) | 10.3 (7.2 to 13.8) | 24.8 (19.4 to 30.4) |
| Stroke | Air pollution | 30.8 (24.6 to 38.1) | 32 (25.5 to 39.2) | 31.3 (25.1 to 38.7) |
|  | Alcohol use | 8.1 (2.1 to 14.9) | 0.9 (0.2 to 1.8) | 5 (1.3 to 9.4) |
|  | Dietary risks | 20.8 (10.6 to 33.9) | 16.9 (7.6 to 28.6) | 19.1 (9.5 to 31.3) |
|  | High LDL cholesterol | 11.3 (4 to 18.7) | 12.3 (4.3 to 20.1) | 11.8 (4.1 to 19.3) |
|  | High body-mass index | 3 (0.3 to 6.3) | 4.2 (0.3 to 8.8) | 3.5 (0.3 to 7.4) |
|  | High fasting plasma glucose | 9.7 (7.5 to 12) | 10 (7.8 to 12.3) | 9.8 (7.6 to 12.1) |
|  | High systolic blood pressure | 56.9 (42.6 to 68.8) | 57.7 (42.6 to 69.5) | 57.3 (42.5 to 68.7) |
|  | Impaired kidney function | 9.1 (6.8 to 11.3) | 9.8 (7.1 to 12.5) | 9.4 (6.9 to 11.9) |
|  | Lead exposure | 9 (-1.2 to 20.1) | 7.3 (-1 to 16.3) | 8.3 (-1.1 to 18.4) |
|  | Low physical activity | 1.7 (0.5 to 3) | 2.5 (0.7 to 4.6) | 2 (0.6 to 3.7) |
|  | Suboptimal temperature | 6 (4.5 to 8.3) | 5.6 (4.2 to 7.9) | 5.8 (4.4 to 8.2) |
|  | Tobacco | 27.9 (24 to 31.6) | 9.3 (6.8 to 11.7) | 19.9 (16.5 to 23.2) |

**Figure S1.** Restricted cubic spline curves showing temporal trends in age-standardized rates of prevalence, incidence, deaths, and DALYs of cardiovascular diseases in Asia (1990-2021). All non-linear P value < 0.05. DALYs=disability adjusted life years.

**Figure S2.** Age-standardised prevalence rate of cardiovascular diseases per 100 000 population in 2021, by country.

**Figure S3.** Age-standardised incidence rate of cardiovascular diseases per 100 000 population in 2021, by country.

**Figure S4.** Age-standardised death rate of cardiovascular diseases per 100 000 population in 2021, by country.

**Figure S5.** Age-standardised DALYs rate of cardiovascular diseases per 100 000 population in 2021, by country. DALYs=disability adjusted life years

**Figure S6.** Age-standardised DALYs rate of rheumatic heart disease per 100 000 population in 2021, by country. DALYs=disability adjusted life years

**Figure S7.** Age-standardised DALYs rate of ischemic heart disease per 100 000 population in 2021, by country. DALYs=disability adjusted life years

**Figure S8.** Age-standardised DALYs rate of stroke per 100 000 population in 2021, by country. DALYs=disability adjusted life years

**Figure S9.** Age-standardised DALYs rate of hypertensive heart disease per 100 000 population in 2021, by country. DALYs=disability adjusted life years

**Figure S10.** Age-standardised DALYs rate of non-rheumatic valvular heart disease per 100 000 population in 2021, by country. DALYs=disability adjusted life years

**Figure S11.** Age-standardised DALYs rate of cardiomyopathy and myocarditis per 100 000 population in 2021, by country. DALYs=disability adjusted life years

**Figure S12.** Age-standardised DALYs rate of pulmonary artery hypertension per 100 000 population in 2021, by country. DALYs=disability adjusted life years

**Figure S13.** Age-standardised DALYs rate of atrial fibrillation and flutter per 100 000 population in 2021, by country. DALYs=disability adjusted life years

**Figure S14.** Age-standardised DALYs rate of aortic aneurysm per 100 000 population in 2021, by country. DALYs=disability adjusted life years

**Figure S15.** Age-standardised DALYs rate of lower extremity peripheral arterial disease per 100 000 population in 2021, by country. DALYs=disability adjusted life years

**Figure S16.** Age-standardised DALYs rate of endocarditis per 100 000 population in 2021, by country. DALYs=disability adjusted life years

**Figure S17.** Age-standardised DALYs rate of other cardiovascular and circulatory diseases per 100 000 population in 2021, by country. DALYs=disability adjusted life years

**Figure S18.** The age-standardised prevalence rate of cardiovascular diseases in 2021 for the Asia countries, by sex.

**Figure S19.** The age-standardised incidence rate of cardiovascular diseases in 2021 for the Asia countries, by sex.

**Figure S20.** The age-standardised death rate of cardiovascular diseases in 2021 for the Asia countries, by sex.

**Figure S21.** The age-standardised DALY rate of cardiovascular diseases in 2021 for the Asia countries, by sex. DALY=disability adjusted life year.

**Figure S22**. Temporal patterns of ischemic heart disease burden in Asia, 1990–2021. (**A**) all-age counts; (**B**) age-standardized rates (per 100,000).

**Figure S23.** The age-standardised DALY rate of ischemic heart disease in 2021 for the Asia countries, by sex. DALY=disability adjusted life year.

**Figure S24.** Age-wise distribution of DALYs attributable to ischemic heart diseases in Asia, 2021. DALY=disability adjusted life years.

**Figure S25**. Temporal patterns of stroke burden in Asia, 1990–2021. (**A**) all-age counts; (**B**) age-standardized rates (per 100,000).

**Figure S26.** The age-standardised DALY rate of stroke in 2021 for the Asia countries, by sex. DALY=disability adjusted life year.

**Figure S27.** Age-wise distribution of DALYs attributable to stroke in Asia, 2021. DALY=disability adjusted life years.

**Figure S28**. Temporal patterns of atrial fibrillation and flutter burden in Asia, 1990–2021. (**A**) all-age counts; (**B**) age-standardized rates (per 100,000).

**Figure S29.** The age-standardised DALY rate of atrial fibrillation and flutter in 2021 for the Asia countries, by sex. DALY=disability adjusted life year.

**Figure S30.** Age-wise distribution of DALYs attributable to atrial fibrillation and flutter in Asia, 2021. DALY=disability adjusted life years.

**Figure S31**. Temporal patterns of hypertensive heart disease burden in Asia, 1990–2021. (**A**) all-age counts; (**B**) age-standardized rates (per 100,000).

**Figure S32.** The age-standardised DALY rate of hypertensive heart disease in 2021 for the Asia countries, by sex. DALY=disability adjusted life year.

**Figure S33.** Age-wise distribution of DALYs attributable to hypertensive heart disease in Asia, 2021. DALY=disability adjusted life years.

**Figure S34.** Temporal patterns of rheumatic heart disease burden in Asia, 1990–2021. (**A**) all-age counts; (**B**) age-standardized rates (per 100,000).

**Figure S35.** The age-standardised DALY rate of rheumatic heart disease in 2021 for the Asia countries, by sex. DALY=disability adjusted life years.

**Figure S36.** Age-wise distribution of DALYs attributable to rheumatic heart diseases in Asia, 2021. DALY=disability adjusted life years.

**Figure S37**. Temporal patterns of non-rheumatic valvular heart disease burden in Asia, 1990–2021. (**A**) all-age counts; (**B**) age-standardized rates (per 100,000).

**Figure S38.** The age-standardised DALY rate of non-rheumatic valvular heart disease in 2021 for the Asia countries, by sex. DALY=disability adjusted life year.

**Figure S39.** Age-wise distribution of DALYs attributable to non-rheumatic valvular disease in Asia, 2021. DALY=disability adjusted life years.

**Figure S40**. Temporal patterns of cardiomyopathy and myocarditis burden in Asia, 1990–2021. (**A**) all-age counts; (**B**) age-standardized rates (per 100,000).

**Figure S41.** The age-standardised DALY rate of cardiomyopathy and myocarditis in 2021 for the Asia countries, by sex. DALY=disability adjusted life year.

**Figure S42.** Age-wise distribution of DALYs attributable to cardiomyopathy and myocarditis in Asia, 2021. DALY=disability adjusted life years.

**Figure S43**. Temporal patterns of pulmonary arterial hypertension burden in Asia, 1990–2021. (**A**) all-age counts; (**B**) age-standardized rates (per 100,000).

**Figure S44.** The age-standardised DALY rate of pulmonary arterial hypertension in 2021 for the Asia countries, by sex. DALY=disability adjusted life year.

**Figure S45.** Age-wise distribution of DALYs attributable to pulmonary artery hypertension in Asia, 2021. DALY=disability adjusted life years.

**Figure S46**. Temporal patterns of aortic aneurysm burden in Asia, 1990–2021. (**A**) all-age counts; (**B**) age-standardized rates (per 100,000).

**Figure S47.** The age-standardised DALY rate of aortic aneurysm in 2021 for the Asia countries, by sex. DALY=disability adjusted life year.

**Figure S48.** Age-wise distribution of DALYs attributable to aortic aneurysm in Asia, 2021. DALY=disability adjusted life years.

**Figure S49**. Temporal patterns of lower extremity peripheral arterial disease burden in Asia, 1990–2021. (**A**) all-age counts; (**B**) age-standardized rates (per 100,000).

**Figure S50.** The age-standardised DALY rate of lower extremity peripheral arterial disease in 2021 for the Asia countries, by sex. DALY=disability adjusted life year.

**Figure S51.** Age-wise distribution of DALYs attributable to lower extremity peripheral arterial disease in Asia, 2021. DALY=disability adjusted life years.

**Figure S52**. Temporal patterns of endocarditis burden in Asia, 1990–2021. (**A**) all-age counts; (**B**) age-standardized rates (per 100,000).

**Figure S53.** The age-standardised DALY rate of endocarditis in 2021 for the Asia countries, by sex. DALY=disability adjusted life year.

**Figure S54.** Age-wise distribution of DALYs attributable to endocarditis in Asia, 2021. DALY=disability adjusted life years.

**Figure S55**. Temporal patterns of other cardiovascular and circulatory diseases burden in Asia, 1990–2021. (**A**) all-age counts; (**B**) age-standardized rates (per 100,000).

**Figure S56.** The age-standardised DALY rate of other cardiovascular and circulatory diseases in 2021 for the Asia countries, by sex. DALY=disability adjusted life year.

**Figure S57.** Age-wise distribution of DALYs attributable to other cardiovascular and circulatory diseases in Asia, 2021. DALY=disability adjusted life years.

**Figure S58.** Percentage of DALYs due to cardiovascular diseases attributable to risk factors among males in Asia countries in 2021. DALY=disability adjusted life years.

**Figure S59.** Percentage of DALYs due to cardiovascular diseases attributable to risk factors among females in Asia countries in 2021. DALY=disability adjusted life years.

**Figure S60.** Percentage of DALYs due to ischemic heart disease attributable to risk factors among males in Asia countries in 2021. DALY=disability adjusted life years.

**Figure S61.** Percentage of DALYs due to ischemic heart disease attributable to risk factors among females in Asia countries in 2021. DALY=disability adjusted life years.

**Figure S62.** Percentage of DALYs due to stroke attributable to risk factors among males in Asia countries in 2021. DALY=disability adjusted life years.

**Figure S63.** Percentage of DALYs due to stroke attributable to risk factors among females in Asia countries in 2021. DALY=disability adjusted life years.

**Figure S64.** Percentage of DALYs due to hypertensive heart disease attributable to risk factors among males in Asia countries in 2021. DALY=disability adjusted life years.

**Figure S65.** Percentage of DALYs due to hypertensive heart disease attributable to risk factors among females in Asia countries in 2021. DALY=disability adjusted life years.

**Figure S66.** Percentage of DALYs due to atrial fibrillation and flutter attributable to risk factors among males in Asia countries in 2021. DALY=disability adjusted life years.

**Figure S67.** Percentage of DALYs due to atrial fibrillation and flutter attributable to risk factors among females in Asia countries in 2021. DALY=disability adjusted life years.

**Figure S68.** Percentage of DALYs due to aortic aneurysm attributable to risk factors among males in Asia countries in 2021. DALY=disability adjusted life years.

**Figure S69.** Percentage of DALYs due to aortic aneurysm attributable to risk factors among females in Asia countries in 2021. DALY=disability adjusted life years.

**Figure S70.** Percentage of DALYs due to lower extremity peripheral arterial disease attributable to risk factors among males in Asia countries in 2021. DALY=disability adjusted life years.

**Figure S71.** Percentage of DALYs due to lower extremity peripheral arterial disease attributable to risk factors among females in Asia countries in 2021. DALY=disability adjusted life years.

**Figure S72.** DALYs of cardiovascular diseases due to high systolic blood pressure in 2021 by age. DALY=disability adjusted life year.

**Figure S73.** DALYs of cardiovascular diseases due to high low density lipoprotein cholesterol in 2021 by age. DALY=disability adjusted life year.

**Figure S74.** DALYs of cardiovascular diseases due to high body-mass index in 2021 by age. DALY=disability adjusted life year.

**Figure S75.** DALYs of cardiovascular diseases due to high fasting plasma glucose in 2021 by age. DALY=disability adjusted life year.

**Figure S76.** DALYs of cardiovascular diseases due to air pollution in 2021 by age. DALY=disability adjusted life year.

**Figure S77.** DALYs of cardiovascular diseases due to suboptimal temperature in 2021 by age. DALY=disability adjusted life year.

**Figure S78.** DALYs of cardiovascular diseases due to lead exposure in 2021 by age. DALY=disability adjusted life year.

**Figure S79.** DALYs of cardiovascular diseases due to tobacco in 2021 by age. DALY=disability adjusted life year.

**Figure S80.** DALYs of cardiovascular diseases due to alcohol use in 2021 by age. DALY=disability adjusted life year.

**Figure S81.** DALYs of cardiovascular diseases due to dietary risks in 2021 by age. DALY=disability adjusted life year.

**Figure S82.** DALYs of cardiovascular diseases due to low physical activity in 2021 by age. DALY=disability adjusted life year.

**Figure S83.** DALYs of cardiovascular diseases due to impaired kidney function in 2021 by age. DALY=disability adjusted life year.

**Figure S84.** Age-standardised DALY rates of cardiovascular diseases for Asia countries, by SDI, in 2021; Expected values based on the SDI and disease rates in all locations are shown as the black line. Each point shows the observed age-standardised DALY rate for each country in 2021. DALY=disability adjusted life year; SDI=socio-demographic index.

**Figure S85.** Age-standardised DALY rates of rheumatic heart disease for Asia countries, by SDI, in 2021; Expected values based on the SDI and disease rates in all locations are shown as the black line. Each point shows the observed age-standardised DALY rate for each country in 2021. DALY=disability adjusted life year; SDI=socio-demographic index.

**Figure S86.** Age-standardised disability adjusted life year (DALY) rates of rheumatic heart disease for Asian regions by sociodemographic index, 1990–2021.

**Figure S87.** Age-standardised DALY rates of ischemic heart disease for Asia countries, by SDI, in 2021; Expected values based on the SDI and disease rates in all locations are shown as the black line. Each point shows the observed age-standardised DALY rate for each country in 2021. DALY=disability adjusted life year; SDI=socio-demographic index.

**Figure S88.** Age-standardised disability adjusted life year (DALY) rates of ischemic heart disease for Asian regions by sociodemographic index, 1990–2021.

**Figure S89.** Age-standardised DALY rates of stroke for Asia countries, by SDI, in 2021; Expected values based on the SDI and disease rates in all locations are shown as the black line. Each point shows the observed age-standardised DALY rate for each country in 2021. DALY=disability adjusted life year; SDI=socio-demographic index.

**Figure S90.** Age-standardised disability adjusted life year (DALY) rates of stroke for Asian regions by sociodemographic index, 1990–2021.

**Figure S91.** Age-standardised DALY rates of hypertensive heart disease for Asia countries, by SDI, in 2021; Expected values based on the SDI and disease rates in all locations are shown as the black line. Each point shows the observed age-standardised DALY rate for each country in 2021. DALY=disability adjusted life year; SDI=socio-demographic index.

**Figure S92.** Age-standardised disability adjusted life year (DALY) rates of hypertensive heart disease for Asian regions by sociodemographic index, 1990–2021.

**Figure S93.** Age-standardised DALY rates of non-rheumatic valvular heart disease for Asia countries, by SDI, in 2021; Expected values based on the SDI and disease rates in all locations are shown as the black line. Each point shows the observed age-standardised DALY rate for each country in 2021. DALY=disability adjusted life year; SDI=socio-demographic index.

**Figure S94.** Age-standardised disability adjusted life year (DALY) rates of non-rheumatic valvular heart disease for Asian regions by sociodemographic index, 1990–2021.

**Figure S95.** Age-standardised DALY rates of cardiomyopathy and myocarditis for Asia countries, by SDI, in 2021; Expected values based on the SDI and disease rates in all locations are shown as the black line. Each point shows the observed age-standardised DALY rate for each country in 2021. DALY=disability adjusted life year; SDI=socio-demographic index.

**Figure S96.** Age-standardised disability adjusted life year (DALY) rates of cardiomyopathy and myocarditis for Asian regions by sociodemographic index, 1990–2021.

**Figure S97.** Age-standardised DALY rates of pulmonary artery hypertension for Asia countries, by SDI, in 2021; Expected values based on the SDI and disease rates in all locations are shown as the black line. Each point shows the observed age-standardised DALY rate for each country in 2021. DALY=disability adjusted life year; SDI=socio-demographic index.

**Figure S98.** Age-standardised disability adjusted life year (DALY) rates of pulmonary artery hypertension for Asian regions by sociodemographic index, 1990–2021.

**Figure S99.** Age-standardised DALY rates of atrial fibrillation and flutter for Asia countries, by SDI, in 2021; Expected values based on the SDI and disease rates in all locations are shown as the black line. Each point shows the observed age-standardised DALY rate for each country in 2021. DALY=disability adjusted life year; SDI=socio-demographic index.

**Figure S100.** Age-standardised disability adjusted life year (DALY) rates of atrial fibrillation and flutter for Asian regions by sociodemographic index, 1990–2021.

**Figure S101.** Age-standardised DALY rates of aortic aneurysm for Asia countries, by SDI, in 2021; Expected values based on the SDI and disease rates in all locations are shown as the black line. Each point shows the observed age-standardised DALY rate for each country in 2021. DALY=disability adjusted life year; SDI=socio-demographic index.

**Figure S102.** Age-standardised disability adjusted life year (DALY) rates of aortic aneurysm for Asian regions by sociodemographic index, 1990–2021.

**Figure S103.** Age-standardised DALY rates of lower extremity peripheral arterial disease for Asia countries, by SDI, in 2021; Expected values based on the SDI and disease rates in all locations are shown as the black line. Each point shows the observed age-standardised DALY rate for each country in 2021. DALY=disability adjusted life year; SDI=socio-demographic index.

**Figure S104.** Age-standardised disability adjusted life year (DALY) rates of lower extremity peripheral arterial disease for Asian regions by sociodemographic index, 1990–2021.

**Figure S105.** Age-standardised DALY rates of endocarditis for Asia countries, by SDI, in 2021; Expected values based on the SDI and disease rates in all locations are shown as the black line. Each point shows the observed age-standardised DALY rate for each country in 2021. DALY=disability adjusted life year; SDI=socio-demographic index.

**Figure S106.** Age-standardised disability adjusted life year (DALY) rates of endocarditis for Asian regions by sociodemographic index, 1990–2021.

**Figure S107.** Age-standardised DALY rates of other cardiovascular and circulatory diseases for Asia countries, by SDI, in 2021; Expected values based on the SDI and disease rates in all locations are shown as the black line. Each point shows the observed age-standardised DALY rate for each country in 2021. DALY=disability adjusted life year; SDI=socio-demographic index.

**Figure S108.** Age-standardised disability adjusted life year (DALY) rates of other cardiovascular and circulatory diseases for Asian regions by sociodemographic index, 1990–2021.
